# Antibacterial Polyketides Isolated from the Marine-Derived Fungus *Fusarium solani* 8388

**DOI:** 10.3390/jof9090875

**Published:** 2023-08-24

**Authors:** Cankai Lin, Rongchun Huang, Juntao Liu, Hong Li, Liping Zhu, Xin Huang, Bo Ding, Lan Liu, Hongbo Huang, Yiwen Tao

**Affiliations:** 1 Guangzhou Municipal and Guangdong Provincial Key Laboratory of Molecular Target & Clinical Pharmacology, and the NMPA Laboratory of Respiratory Disease, School of Pharmaceutical Sciences and the Fifth Affiliated Hospital, Guangzhou Medical University, Guangzhou 511436, China; 2 School of Marine Sciences, Sun Yat-Sen University, Zhuhai 519082, China

**Keywords:** polyketide, antibacterial, cytotoxicity, marine microorganism, fungus, *Fusarium solani*

## Abstract

Seven new polyketides named fusarisolins F-K (**1**–**6**) and fusarin I (**7**) were isolated from the marine-derived fungus *Fusarium solani* 8388, together with the known anhydrojavanicin (**8**), 5-deoxybostry coidin (**9**), and scytalol A (**10**). Their structures were established by comprehensive spectroscopic data analyses, and by comparison of the ^1^H and ^13^C NMR data with those reported in literature. Fusarisolin F (**1**) contained both a dichlorobenzene group and an ethylene oxide unit, which was rare in nature. In the bioassays, fusarisolin I (**4**), fusarisolin J (**5**), and 5-deoxybostry coidin (**9**) exhibited obvious antibacterial activities against methicillin-resistant *Staphylococcus aureus* n315 with MIC values of 3, 3, and 6 μg/mL, respectively. Fusarisolin H (**3**) and fusarisolin J (**5**) showed inhibitory effects against methicillin-resistant *Staphylococcus aureus* NCTC 10442 with the same MIC value of 6 μg/mL. With the exception of **5**, all other compounds did not show or showed weak cytotoxicities against HeLa, A549, and KB cells; while fusarisolin J (**5**) demonstrated moderate cytotoxicities against the three human cancer cell lines with CC_50_ values between 9.21 and 14.02 μM.

## 1. Introduction

The deep sea is a special environment with high pressure, high salt, low temperature, low oxygen concentration, darkness, and oligotrophic conditions. The microorganisms that inhabit the deep sea are usually obviously different with those living on the mainland. The severe growth environment allows microorganisms to produce and accumulate various secondary metabolites with novel chemical structures and potent physiological activities. Therefore, marine microorganisms have been considered as a reservoir of bioactive secondary metabolites [1,2]. *Fusarium* species are ubiquitous in both marine and terrestrial environments, including deserts and the Arctic [3], which produce mycotoxins as deoxynivalenol, zearalenone, fumonisin B1, and T-2 toxin which causes the risks of bakanae, foot rot, scab, and head blight [4,5]. In addition, *Fusarium* species possess the potential capability to produce structurally diverse secondary metabolites such as polyketides, alkaloids, terpenoids, peptides, and steroids with potent physiological activities [3,6]. Polyketides are a class of natural secondary metabolites synthesized by polyketide synthases, which have diverse frameworks and outstanding pharmacological activities. Some marine-derived polyketide compounds have already played important roles for the development of new drugs [7,8,9]. For example, the polyketide salinosporamide A, isolated from a marine actinomycete, is a potent proteasome inhibitor and is now in clinical trials for the treatment of brain cancer [10]. Abyssomicin C, a polyketide from marine actinomycetes, demonstrates significant antibacterial activity against the methicillin- and vancomycin-resistant *Staphylococcus aureus* (MRSA and VRSA) strains by inhibiting formation of *p*-aminobenzoate [11,12]. Therefore, it is of great significance to carry out the secondary metabolites of *Fusarium* species from marine environments.

**Figure 1:**
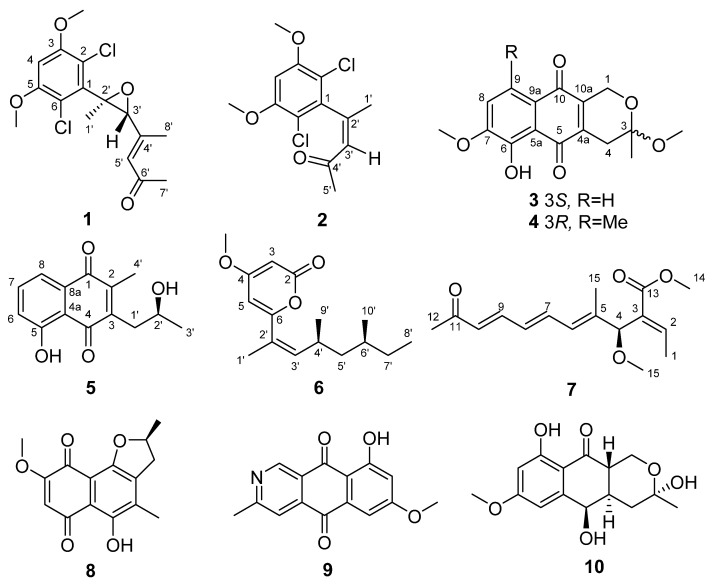
Chemical structures of **1**–**10** isolated from the *Fusarium solani* 8388.

In our ongoing research, a fungus strain identified as *Fusarium solani* 8388 obtained from the Shenhu area in the Northern South China Sea showed an abundant metabolite profile when analyzed by high-performance liquid chromatography (HPLC). This fungus was cultured by using rice medium. Subsequently, seven new polyketides (**1**–**7**) were identified, together with known compounds (**8**–**10**) (Figure 1). In the bioassays, these compounds showed antibacterial activities against methicillin-resistant *Staphylococcus aureus* (MRSA). We report herein the fermentation, isolation, structure elucidation, and biological activities of these marine-derived fungal polyketide compounds.

## 2. Results and Discussion

**Figure 2:**
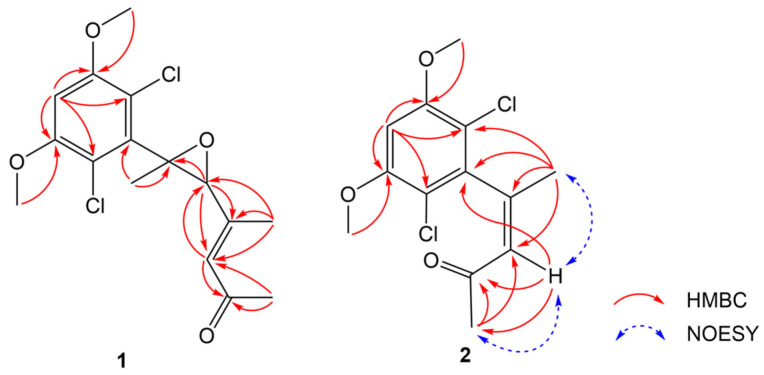
Selected HMBC correlations of **1** and **2**, and key NOE correlations of **2**.

**Figure 3:**
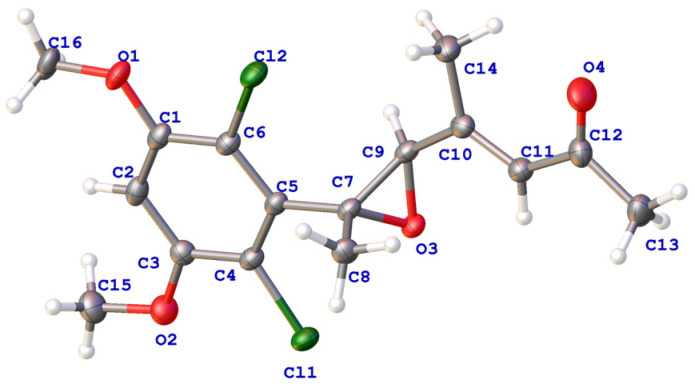
The ORTEP drawing of the crystal structure of compound **1**.

Compound **1** was isolated as a colorless crystal. The molecular formula of **1** was determined to be C_16_H_18_Cl_2_O_4_ on the basis the protonated molecular ion peak at *m*/*z* 345.0655 [M + H]^+^ (calcd. for C_16_H_19_Cl_2_O_4_^+^, 345.0655) and the sodium adducted ion peak at *m*/*z* 367.0474 [M + Na]^+^ (calcd. for C_16_H_18_Cl_2_NaO_4_^+^, 367.0474) in the (+)HRESIMS spectrum, inferring the presence of 7 degrees of unsaturation. The typical isotopic abundance ratio of peaks [M + H]^+^, [M + 2 + H]^+^, and [M + 4 + H]^+^approximated 9:6:1 (Figure S1), which indicated that compound **1** contained two chlorine atoms. The ^1^H NMR spectrum of **1** was characterized by resonances consistent with two methines at *δ*_H_ 6.55 (H-4) and 6.39 (s, H-5′), an aliphatic methane at *δ*_H_ 3.55 (H-3′), two methoxy groups at *δ*_H_ 3.93 (3-OMe and 5-OMe, 6H), and three methyl groups at *δ*_H_ 2.35, 2.25, and 1.50 (H_3_-8′, H_3_-7′, and H_3_-1′) (Table 1). The ^13^C NMR spectroscopic data revealed signals corresponding to a carbonyl (*δ*_C_ 198.9, C-6′), eight aromatic carbons (C-1~C-6, C-4′, and C-5′), two oxygen-bearing carbons (C-2′ and C-3′), two methoxy carbons (*δ*_C_ 56.7, 3-OMe; *δ*_C_ 56.8, 5-OMe), and three methyl carbons (*δ*_C_ 32.3, C-7′, *δ*_C_ 17.2, C-8′; *δ*_C_ 15.7, C-1′). In the HMBC spectrum, the correlations from H_3_-1′ to C-2′, from H-3′ to C-2′, C-4′, C-5′, from H_3_-8′ to C-3′, C-4′, C-5′, from H-5′ to C-3′, C-8′, C-6′, and from H_3_-7′ to C-6′, C-5′ established the keto chain of CH_3_-1′/C-2′/CH-′3/C-4′(CH_3_-8′)/CH-5′/C-6′/CH_3_-7′ (Figure 2). The remaining six aromatic carbons including a methine carbon (CH-4) suggested the presence of a penta-substituted benzene ring. The HMBC correlations of H-4 to C-3, C-5, C-2, and C-4 confirmed the phenyl group in **1**. The HMBC association of H_3_-1′/C-1 connected the keto chain with the phenyl group by the linkage of C-2′/C-1. The positions of 3-OMe and 5-OMe were confirmed by the HMBC correlations from the methoxy protons at *δ*_H_ 3.93 to C-3 and C-5. To meet the requirement of the molecular formula of C_16_H_18_Cl_2_O_4_, two chlorine substitutions at C-2 and C-5 and an epoxide group between C-2′ and C-4′ were presumed, which finally completed the establishment of the planar structure of **1**. After careful incubation in MeOH, single crystals of **1** were obtained. Analysis of the X-ray diffraction data confirmed the presence of two chlorine atoms and the epoxide group (Figure 3). In addition, the Me-1′ and H-3′ were placed to the opposite side of the epoxide ring. Furthermore, the double bond of C-4′/C-5′ was confirmed to be *E*-configuration on the basis of X-ray diffraction. Compound **1** was named fusarisolin F.

Compound **2** was isolated as a colorless solid. The (+)HRESIMS spectrum showed a protonated ion peak at *m*/*z* 289.0394 [M + H]^+^ (calcd. for C_13_H_15_Cl_2_O_3_^+^, 289.0393) and the sodium adducted ion peak at *m*/*z* 311.0212 [M + Na]^+^ (calcd. for C_13_H_14_Cl_2_NaO_3_^+^, 311.0212), establishing the molecular formula of C_13_H_14_Cl_2_O_3_ for **2**. The similar typical isotopic clusters with compound **1** in MS spectrum (Figure S2) inferred that compound **2** also contained two chlorine atoms. The ^1^H and ^13^C NMR spectroscopic data of **2** (Table 1) showed closely similar signals (C-1~C-6) to those of **1**, which revealed the presence of a penta-substituted benzene ring such as that in **1**. Furthermore, chemical resonances for two methyls (*δ*_H_ 2.11, *δ*_C_ 29.7, Me-1′; *δ*_H_ 1.96, *δ*_C_ 24.3, Me-5′), one aromatic methine (*δ*_H_ 6.32, *δ*_C_ 129.4, CH-3′), a non-protonated carbon at *δ*_C_ 148.2 (C-2′), and a carbonyl at *δ*_C_ 197.1 (C-4′) were observed. In the HMBC spectrum, the correlations from H_3_-5′ to C-3′, C-5, from H-3′ to C-4′, C-5′, and from H_3_-1′ to C-2′, C-3′ constructed the keto chain of CH_3_-1′/C-2′/CH-3′/C-4′/CH_3_-5′, in the same fashion as that in **1**. Additionally, the HMBC correlations from H_3_-5′ to C-1 and C-2, and from H-3′ to C-1 placed the keto chain to the phenyl group via the linkage of C-2′/C-1. Thus, the planar structure of **2** was established, which was the same as that of T5 in a Chinese patent application [13]. However, the observed NOE correlation of H_3_-1′/H-3′ in the NOESY spectrum suggested the *Z*-configuration of the double bond between C-2′ and C-3′, which was opposite with the *E*-configuration in T5. In addition, the ^1^H and ^13^C chemical data of the Me-1′ (*δ*_H_ 2.11, *δ*_C_ 29.7) and Me-5′ (*δ*_H_ 1.95, *δ*_C_ 24.3) in **2** were obviously different to that reported for Me-1′ at *δ*_H_ 2.29 and *δ*_C_ 18.3, and for Me-5′ at *δ*_H_ 2.25 and *δ*_C_ 30.7 in T5. Therefore, compound **2** was identified as (*Z*)-4-(2,6-dichloro-3,5-dimethoxyphenyl)pent-3-en-2-one, and given the name fusarisolin G.

**Figure 4:**
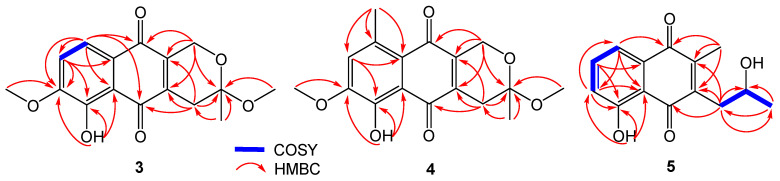
Selected COSY and HMBC correlations of compounds **3**–**5**.

**Figure 5:**
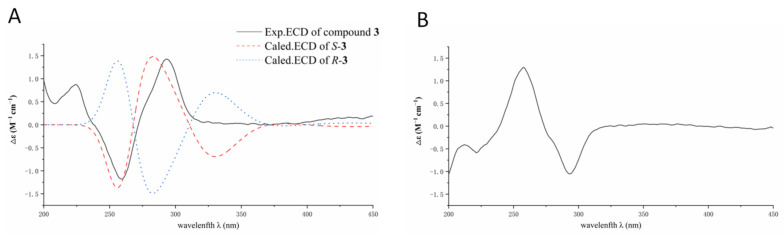
(**A**) Comparison of the experimental and the calculated ECD spectra of **3**; (**B**) the ECD spectrum of **4**.

Compound **3** was isolated as a red powder. The (−)HRESIMS spectrum showed signal at *m*/*z* 303.0877 [M − H]^−^ (calcd. for C_16_H_15_O_6_^−^, 303.0874), established the molecular formula of C_16_H_16_O_6_ for **3** with the aid of ^13^C NMR data, inferring 9 degrees of unsaturation. The ^1^H and ^13^C NMR spectroscopic data of **3** (Table 2) showed resonances consistent with a hydrogen-bonded phenol moiety at *δ*_H_ 12.45 (br s, OH-6), a methyl (*δ*_H_ 1.52, *δ*_C_ 22.9, Me-3), two methoxyls (*δ*_H_ 3.98, *δ*_C_ 49.0, OMe-3, *δ*_H_ 3.29, *δ*_C_ 56.5, OMe-7), two methylenes (CH_2_-1; CH_2_-4), two aromatic methines (*δ*_H_ 7.05, *δ*_C_ 115.0, CH-8; *δ*_H_ 7.63, *δ*_C_ 120.6, CH-9), six non-protonated aromatic carbons, two carbonyl carbon at *δ*_C_ 181.6 (C-10) and *δ*_C_ 189.4 (C-5), and a ketal or a hemiketal carbon at *δ*_C_ 97.2 (C-3). The comparison of these NMR data with those reported for 3-methyl ether fusarubin showed close similarity [14], inferring a pyranonaphthoquinone framework for **1**. The HMBC correlations originated from H_2_-1, H_2_-4, H-8, H-9, and the hydrogen atom of OH-6 confirmed the elucidation of pyranonaphthoquinone core (Figure 4). The location of OMe-3, Me-3, and OMe-7 were determined by the HMBC correlations of OMe-3/C-3, Me-3/C-3, and OMe-7/C-7, respectively. Thus, the planar structure of **3** was established. The absolute configuration of the stereogenic center at C-3 was assigned as *S* on the basis of the ECD curve of **3** showed good agreement of the calculated one for 3*S*-**3** (Figure 5A). Compound **3** was named fusarisolin H.

Compound **4** was obtained as a red powder. Its molecular formula C_17_H_18_O_6_ was determined by the quasimolecular ion peak at *m*/*z* 317.1034 [M − H]^−^ (calcd. for C_17_H_17_O_6_^−^, 317.1031) observed in the (−)HRESIMS spectrum, representing a 14-mass unit gain relative to **3**. The ^1^H and ^13^C NMR spectroscopic data of **4** were very similar to those of **3** (Table 2), except that the additional signals at *δ*_H_ 2.65 and *δ*_C_ 23.4 (Me-9) attributable to a methyl group were observed in **4**. Moreover, the aromatic proton at *δ*_H_ 7.63 (H-9) in **3** had disappeared in **4**. In addition, the ^13^C NMR signal of C-9 was shifted downfield from *δ*_C_ 120.6 in **3** to 136.6 in **4**. These changes indicated that H-9 in **3** was replaced a methyl group in **4**. In the HMBC spectrum, the correlation from the new appearing methyl protons to C-8, C-9, and C-9a confirmed the presence of the Me-9. The ECD spectrum of **4** showed a negative Cotton effect at 260 nm and positive Cotton effect at 293 nm (Figure 5B), which were contrary to those of **3**, establishing 3*R* configuration for **4**. Compound 4 was named fusarisolin I.

**Figure 6:**
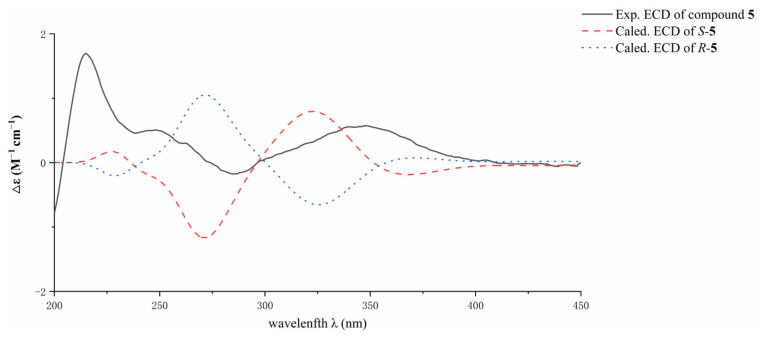
Comparison of the experimental and the calculated ECD spectra of **5**.

Compound **5** was isolated as a yellow powder. The (+)HRESIMS spectrum displayed a quasimolecular ion peak at *m*/*z* 247.0987 [M + H]^+^ (calcd. for C_14_H_15_O_4_^−^, 247.0965), which suggested the molecular formula of C_14_H_14_O_4_ for **5**. The ^1^H NMR spectrum of **5** (Table 3) was characterized by resonances consistent with a hydrogen-bonded phenol moiety at *δ*_H_ 12.10 (br s, OH-5), three aromatic methine protons at *δ*_H_ 7.20 (H-6), 7.57 (H-7), and 7.58 (H-8), an oxygen-bearing methine proton at *δ*_H_ 4.07(H-2′), a methylene protons at *δ*_H_ 2.82 (H_2_-1′), and two methyl protons at *δ*_H_ 2.22 (Me-2) and 1.32 (H_3_-3′). The ^13^C NMR spectrum revealed signals corresponding to two carbonyls at *δ*_C_ 190.8 (C-4) and 184.5 (C-1), three aromatic methine carbons, and five non-protonated aromatic carbons, which were attributable to a naphthoquinone scaffold [15]. In addition, three aliphatic carbon signals, including an oxygen-bearing methine carbon at *δ*_C_ 67.9 (C-2′), a methylene carbon at *δ*_C_ 36.2 (C-1′), and a methyl carbon at *δ*_C_ 24.4 (C-3′) were observed. The COSY correlations of H-6/H-7/H-8, together with the HMBC correlations from H-6 to C-8, from H-7 to C-5, C-8a, from H-8 to C-6, C-4a, C-1, from 5-OH to C-4a, C-5, C-6, and from Me-2 to C-1, C-2, C-3 established a 2,3-disubstituted-5-hydroxy naphthoquinone skeleton. In addition, the COSY correlations of H_2_-1′/H-2′/H_3_-3′ confirmed the presence of the side chain of CH*_2_*-1′/CH-2′/CH_3_-3′. The HMBC correlations of H_2_-1′ to C-2, C-3, C-4 revealed the location of the side chain at C-3. The absolute configuration of the stereogenic center at C-2′ was determined by calculation of ECD spectra. The experimental ECD spectrum of **5** was in good agreement with that of (2′*S*)-**5** (Figure 6), establishing *S* configuration for C-2′. Compound **5** was elucidated as (*S*)-5-hydroxy-3-(2-hydroxypropyl)-2-methyl naphthalene-1,4-dione and given the name fusarisolin J.

**Figure 7:**
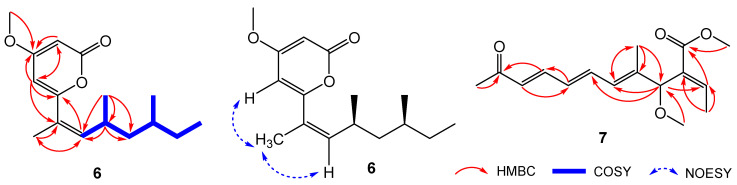
Selected COSY and HMBC correlations of compounds **6** and **7**, and NOESY correlations of compound **6**.

Compound **6** was isolated as orange oil. The (+)HRESIMS spectrum of **6** displayed a protonated ion peak at *m*/*z* 265.1790 [M + H]^+^ (calcd. for C_16_H_25_O_3_^+^, 265.1798) and the sodium adducted ion peak at *m*/*z* 287.1607 [M + Na]^+^ (calcd. for C_16_H_24_O_3_Na^+^, 287.1618), indicated the molecular formula of C_16_H_24_O_3_ for **6**, with 5 degrees of unsaturation. The ^1^H and ^13^C NMR data of **6** (Table 4) revealed the presence of four methyls (Me-1′, Me-8′, Me-9′, Me-10′), one methoxyl (OMe-4), two methylene (CH_2_-4′ and CH_2_-6′), three aromatic (CH-3, CH-5, CH-3′) and two aliphatic methines (CH-4′ and CH-6′), three non-protonated carbons (C-4, C-6, C-2′), and a carbonyl (C-2). The COSY spectrum established a long alkyl chain of C-3′~C-8′ with substitutions of two methyl groups at C-4′ and C-6′ (Figure 7). The HMBC correlations from the protons of Me-1′ to C-2′ and C-3′, as well as the ^1^H NMR chemical shift of Me-1′ at *δ*_H_ 1.85, confirmed the structure of the entire side chain (C-1′~C-8′). Further HMBC correlations from H-3 to C-4, and C-5, and from H-5 to C-3, C-4, and C-6 established a buta-1,3-diene fragment (C-3~C-6). The ^13^C NMR chemical shift values of C-4 at *δ*_C_ 171.7 and C-6 at *δ*_C_ 161.5 indicated C-4 and C-6 were oxygen-bearing carbons. The remaining one degree of unsaturation suggested the presence of a ring in **6**. Importantly, the HMBC correlation from H-3 to the carbonyl (C-2) was observed. The carbonyl was connected to C-6 through an ester bond, constructing a 2*H*-pyran-2-one scaffold. The HMBC correlation of OMe-4/C-4 validated the position of OMe-4. The HMBC correlations from H-5 to C-2′ and from H_3_-1′ and H-3′ to C-6 confirmed the linkage of C-6/C-2′, inferring the position of the side chain at C-6.

**Figure 8:**
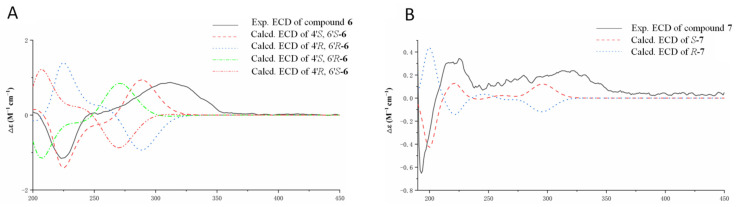
Comparison of the experimental and calculated ECD spectra of **6** (**A**) and **7** (**B**).

In the NOESY spectrum, the correlation between H-3′ and H_3_-1′ was observed, confirming the *E* configuration of the double bond between C-2′ and C-3′. The absolute configurations of the stereogenic centers at C-4′ and C-6′ in **6** were determined by ECD calculation. Based on the experimental ECD spectrum of **6** being consistent with that of 4′*S*,6′*S*-**6** (Figure 8A), the configurations of 6 were assigned as 4′S, 6′S. Compound **6** was designated as fusarisolin K.

Compound **7** was isolated as yellow oil. It showed a sodium adducted ion peak at *m*/*z* 301.1411 [M + Na]^+^ (calcd. for C_16_H_22_NaO_4_^+^, 301.1410) in the (+)HRESIMS spectrum. The molecular formula of **7** was established to be C_16_H_22_O_4_, indicating 6 degrees of unsaturation. The ^1^H and ^13^C NMR spectroscopic data of **7** (Table 4) were characterized by chemical resonances consistent with three methyls (Me-1, Me-12, Me-15), two methoxyls (OMe-14, OMe-16), six aromatic and one aliphatic methines, two non-protonated carbons, and one keto and one esteric carbonyls (C-11, C-13). These data were similar with those for fusarin J [16], except one more signal attributed for a methoxyl at *δ*_H_ 3.33 and *δ*_C_ 56.3 (OMe-16) was observed in **7**. Detailed analyses of the HMBC correlations for **7** (Figure 7) placed the new-appearing methoxyl at C-4 and confirmed the structure of **7**. Compound **7** showed a positive specific rotation value, which was the same as that of fusarin J, indicating the 4*S* configuration. In addition, the experimental ECD spectrum of **7** was coincidental with the calculated one of 4*S*-**7** (Figure 8B), confirmed the determination of 4*S* configuration. Compound **7** was named fusarin I.

The three known compounds were identified to be anhydrojavanicin (5-hydroxy-8-methoxy-2,4-dimethylnaphtho[1,2-*b*]furan-6,9-dione (**8**) [15], 5-deoxybostrycoidin (**9**) [17], and scytalol A (**10**) [18] by comparison of the ^1^H and ^13^C NMR data with those reported.

All these compounds were measured for their antibacterial activities against Gram-negative bacteria *Escherichia coli* ATCC 25922 and three Gram-positive bacteria including *Staphylococcus aureus* ATCC 29213, the methicillin-resistant *Staphylococcus aureus* NCTC 10442, and methicillin-resistant *Staphylococcus aureus* n315 using 2-fold serial dilution assays. Fusarisolin I (**4**) and fusarisolin J (**5**) exhibited strong inhibitory activity against methicillin-resistant *Staphylococcus aureus* n315 with the same MIC value of 3 µg/mL. Fusarisolin H (**3**) and fusarisolin J (**5**) displayed antibacterial activities against methicillin-resistant *Staphylococcus aureus* NCTC 10442, both with MIC value of 6 µg/mL. Furthermore, compound **9** showed antibacterial activity against methicillin-resistant *Staphylococcus aureus* n315 with a MIC value of 6 µg/mL (Table 5). However, all the isolates did not show antibacterial activity toward the Gram-negative bacteria *Escherichia coli* ATCC 25922 under the concentrations of 50 µg/mL.

In addition, these isolates were tested for cytotoxicities against human lung adenocarcinoma cell line A549, human cervical carcinoma cell line HeLa, and human nasopharyngeal carcinoma cell line KB using MTT colorimetric assays. Fusarisolin J (**5**) inhibited cell proliferation of HeLa, A549, and KB with CC_50_ values of 9.21, 14.02, and 12.07 µM, respectively. With the exception of **5**, other compounds did not show or showed weak cytotoxicities against the three human cancer cell lines (Table 6).

## 3. Materials and Methods

### 3.1. General Experimental Procedures

UV spectra were obtained using a UV-2600 UV-Vis spectrophotometer (Shimadzu, Kyoto, Japan). Optical rotations were obtained with a P850 automatic polarimeter (Haineng, Jinan, China). ECD data were recorded with a Chirascan V100 spectrometer (Chirascan, Surrey, UK). NMR spectra were recorded with a JNM-ECZ 400NB nuclear magnetic resonance spectrometer (JEOL, Tokyo, Japan) at 400 MHz for ^1^H nuclei and 100 MHz for ^13^C nuclei. Chemical shifts (*δ*) are given concerning the signal of solvent residue. Mass spectra were obtained using a Q Executive Focus mass spectrometer (Thermo Fisher, Waltham, MA, USA). Semi-preparative HPLC was operated with LC-20A (Shimadzu (China), Shanghai, China) instrument and an Ultimate XB-C18 column (10 × 250 mm, 5 μm, Welch, Shanghai, China). Column chromatography (CC) was performed using silica gel (100–200 or 300–400 mesh, Jiangpeng Silica Gel Company, Yantai, China). All chemicals and solvents were of analytical or chromatographic grade.

### 3.2. Fungal Identification, Fermentation, and Extract

The fungus *Fusarium solani* 8388 was isolated from sediments collected in the Shenhu area of the South China Sea at a depth of 100 m. First, 1 g of sediment was suspended in 100 mL of autoclaved sea water. Then 1 mL of suspension was diluted with 100 mL of autoclaved sea water. After that, 1 mL of dilution was added into 20 mL of PDA medium, which containing chloramphenicol at a concentration of 100 mg/L. The PDA medium plate was cultured at 28 °C for 5 days. The cultured mycelia were purified to obtain single colony of strain 8388 using PDA medium. The internal transcribed spacer (ITS) region was amplified and sequenced using the general primers ITS1 and ITS4. The ITS region of the fungus was a 572-bp DNA sequence (GenBank accession number: KT336512), which showed 99.07% identity to *Fusarium solani*. The cladogram is shown in Figure S8 in Supplementary Materials. The strain was deposited at the School of Pharmacy, Guangzhou Medical University.

The producing strain was incubated on a potato dextrose agar medium plate under 28 °C for 3 days. Then, the fresh mycelia were inoculated to a 250 mL Erlenmeyer flask containing 0.2 g of peptone, 0.1g of yeast extract, 1.0 g of glucose, and 100 mL of seawater. The flasks were incubated on a rotating shaker at 28 °C for 7 days to produce mycelia. The mycelia were inoculated to 1 L Erlenmeyer flasks containing 100 g of rice, 0.5 g of yeast extract, 0.5 g of glucose, 3 g of crude salt, and 200 mL of water. In total, 200 flasks were used. The flasks were incubated statically at 28 °C for 32 days. The fermented cultures were extracted with MeOH three times. After evaporation under reduced pressure, the extract was re-dissolved in water and extracted with EtOAc (1:1) three times. The EtOAc layer was evaporated under reduced pressure to obtain 320 g of extract.

### 3.3. Isolation and Purification

The extract was separated by a silica gel column chromatography (CC) eluting with a series of isocratic petroleum ether-EtOAc (10:90 → 0:100, *v*/*v*) to obtain nine fractions (Fr.1–Fr.9). Fr.1 (10.4 g) was suspended in petroleum ether and centrifuged to obtain compound **8** (120.7 mg, 0.038%). Fr.2 (1.4 g) was chromatographed over a silica gel CC eluting with petroleum ether-EtOAc (2:1, *v*/*v*) to obtain four subfractions (Fr.2-1–Fr.2-4). Fr.2-1 was suspended in MeOH and centrifuged to obtain compound **3** (10.3 mg, 0.0032%). Fr.2-3 was purified by semi-preparative HPLC with an ODS column (10 mm × 250 mm), eluting with a gradient of MeCN-H_2_O (80:30→100:0, *v*/*v*) over 40 min at a flow rate of 2 mL/min to obtain compound **6** (6.2 mg, 0.0019%). Fr.2-4 was further purified by semi-preparative HPLC eluting with a gradient of MeCN-H_2_O (70:30→100:0, *v*/*v*) over 40 min at a flow rate of 2 mL/min to obtain compounds **2** (11.0 mg, 0.0034%), **9** (6.8 mg, 0.0021%), and **4** (5.2 mg, 0.0016%). Fr.2-2 was purified by Sephadex LH-20 gel CC eluting with CH_2_Cl_2_-MeOH (50:50, *v*/*v*) to obtain compound **1** (5.7 mg, 0.0018%). Fr.3 (7.3 g) was chromatographed over silica gel CC using petroleum ether-EtOAc isocratic elution (3:1, *v*/*v*) to obtain three subfractions (Fr.3-1–Fr.3-3). Fr.3-2 was purified by semi-preparative HPLC, eluting with a gradient of MeCN-H_2_O (50:50→70:30, *v*/*v*) over 30 min at a flow rate of 2 mL/min to obtain compounds **7** (7.7 mg, 0.0024%), **5** (14.7 mg, 0.0045%), and **10** (6.8 mg, 0.0021%).

Fusarisolin F (**1**): colorless crystal; m.p. 155–156 °C; [α${]}_{D}^{26}$ +93 (c 0.03, MeOH); UV(MeOH) λmax (log ε) 297 (3.2), 239 (3.9), 205 (4.2) nm; ^1^H and ^13^C NMR spectroscopic data, see Table 1; (+)-HRESIMS *m*/*z* 345.0655 [M + H]^+^ (calcd. for C_16_H_19_Cl_2_O_4_^+^, 345.0655), 367.0474 [M + Na]^+^ (calcd. for C_16_H_18_Cl_2_NaO_4_^+^, 367.0474).

Fusarisolin G (**2**): colorless solid; UV(MeOH) λmax (log ε) 293 (3.4), 201 (4.6) nm; ^1^H and ^13^C NMR spectroscopic data, see Table 1; (+)-HRESIMS *m*/*z* 289.0394 [M + H]^+^ (calcd. for C_13_H_15_Cl_2_O_3_^+^, 289.0393), 311.0212 [M + Na]^+^ (calcd. for C_13_H_14_Cl_2_NaO_3_^+^, 311.0212).

Fusarisolin H (**3**): red powder; [α${]}_{D}^{26}$ +184 (c 0.03, MeOH); UV(MeOH) λmax (log ε) 451 (3.5), 272 (4.1), 198 (4.7) nm; ^1^H and ^13^C NMR spectroscopic data, see Table 2; ECD (MeOH) λmax (Δϵ) 293 (−21.07), 258 (+25.94), 221 (−11.76), 194 (−28.46) nm; (−)-HRESIMS *m*/*z* 303.0877 [M − H]^−^ (calcd. for C_16_H_15_O_6_^−^, 303.0874).

Fusarisolin I (**4**): red powder; [α${]}_{D}^{26}$ −180 (c 0.03, MeOH); UV(MeOH) λmax (log ε) 449 (3.3), 197 (4.7) nm; ^1^H and ^13^C NMR spectroscopic data, see Table 2; ECD (MeOH) λmax (Δϵ) 293 (+21.40), 260 (−17.67), 221 (+13.14), 194 (+19.74) nm; (−)-HRESIMS *m*/*z* 317.10342 [M − H]^−^ (calcd. for C_17_H_17_O_6_^−^, 317.1031).

Fusarisolin J (**5**): yellow powder; [α${]}_{D}^{26}$ +301 (c 0.06,MeOH); UV(MeOH) λmax (log ε) 417 (3.6), 276 (4.1), 245 (4.0), 199 (4.6) nm; ^1^H and ^13^C NMR spectroscopic data, see Table 3; ECD (MeOH) λmax (Δϵ) 348 (+11.49), 285 (−3.53), 248 (+10.14), 215 (+33.97), 197 (−19.32) nm; (+)-HRESIMS *m*/*z* 247.0987 [M + H]^+^ (calcd. for C_14_H_15_O_4_^−^, 247.0965).

Fusarisolin K (**6**): orange oil; [α${]}_{D}^{26}$ +297 (c 0.03,MeOH); UV(MeOH) λmax (log ε) 301 (3.6),198 (4.6) nm; ^1^H and ^13^C NMR spectroscopic data, see Table 4; ECD (MeOH) λmax (Δϵ) 312 (+43.65), 223 (−57.81) nm; (+)-HRESIMS *m*/*z* 265.1790 [M + H]^+^ (calcd. for C_16_H_25_O_3_^+^, 265.1798).

Fusarin I (**7**): yellow oil; [α${]}_{D}^{26}$ +200 (c 0.03, MeOH); UV(MeOH) λmax (log ε) 198 (4.6) nm; ^1^H and ^13^C NMR spectroscopic data, see Table 4; ECD (MeOH) λmax (Δϵ) 322 (+0.47), 280 (+0.39), 225 (+0.68), 193 (−1.30) nm; (+)-HRESIMS *m*/*z* 301.1411 [M + Na]^+^ (calcd. for C_16_H_22_NaO_4_^+^, 301.1410).

### 3.4. X-ray Diffraction

Colorless crystals of **1** were obtained from MeOH by slow evaporation. The crystal data were collected on an Agilent Gemini Ultra diffractometer with Cu Kα radiation (λ = 1.54184 Å) at 170.00(10) K. The crystal structure was solved with the SHELXT structure solution program using Intrinsic Phasing and refined with the SHELXL refinement package using Least Squares minimisation [19,20].

Crystal Data for **1**: C_16_H_18_Cl_2_O_4_ (*M* = 345.20 g/mol), triclinic, space group P-1 (no. 2), *a* = 11.5915(3) Å, *b* = 11.8707(4) Å, *c* = 12.5216(3) Å, *α* = 105.115(2)°, *β* = 95.309(2)°, *γ* = 98.058(2)°, *V* = 1631.85(8) Å^3^, *Z* = 4, *T* = 170.00(10) K, *μ*(Cu-Kα) = 3.713 mm^−1^, *D_calc_* = 1.405 g/cm^3^, 34,106 reflections measured (7.38° ≤ 2θ ≤ 148.062°), 6474 unique (*R*_int_ = 0.0860, R_sigma_ = 0.0443) which were used in all calculations. The final *R*_1_ was 0.0715 (I > 2σ(I)) and *wR*_2_ was 0.1802. Crystallographic data have been deposited with the Cambridge Crystallographic Data Centre with deposition numbers CCDC 2280258. Copies of the data can be obtained, free of charge, on application to the Director, CCDC, 12 Union Road, Cambridge CB2 1EZ, UK [fax: +44(0)-1233-336033 or e-mail: deposit@ccdc.cam.ac.uk]. Crystal data and structure refinements for **1** are listed in Tables S1-1–S1-7 in Supplementary Materials.

### 3.5. Calculation of ECD

MOE 2019 software was used for conformational search. The geometries of all conformers for ECD calculations were optimized sequentially using Gaussian 09W software at RHF/6-31G(d,p) level. The TDDFT method was employed for the ECD calculations of these compounds at the RB3LYP/6-31G (d,p) level in methanol [21].

### 3.6. Antibacterial Assays

The antibacterial activities of compounds **1**–**10** were assessed against *Escherichia coli* ATCC 25922, *Staphylococcus aureus* ATCC 29213*,* the methicillin-resistant strains *Staphylococcus aureus* NCTC 10442 and *Staphylococcus aureus* n315 using a sequential 2-fold serial dilution method, in which compounds were tested at final concentrations ranging from 100 to 0.7 µg/mL. Compounds were dissolved in DMSO, serially diluted in Mueller–Hinton (M-H) broth. The test was conducted in triplicate using 96-well plates; each well contained 200 μL of liquid. Amoxicillin and vancomycin were used as positive controls. DMSO in M-H broth was used as blank control [22].

### 3.7. Cytotoxic Assays

The cytotoxic activities of compounds **1**–**10** were evaluated using the MTT colorimetric assay against A549, HeLa, and KB human tumor cells using the previously reported MTT method [23]. Briefly, human tumor cells were seeded in 96-well plates at a density of 2.5 × 10^4^ cells/mL and incubated at 37 °C in a humidified incubator (5% CO_2_) for 24 h. After that, various concentrations of compounds were added and incubated for 48 h. Then, 20 μL of MTT solution (5 mg/mL) was added to each well, and the cells were further incubated for 4 h. The culture supernatant was removed, and 100 μL of DMSO was added to dissolve the MTT-formazan crystals. Cell growth inhibition was measured by recording the absorbance at λ = 540 nm using a microplate reader and calculated using the following equation: growth inhibition = (1 − OD of treated cells/OD of control cells) × 100%. The half maximal inhibitory concentration (CC_50_) values were obtained from the concentration-response curves, which were plotted for each tested compound using software GraphPad Prism 9.0. The results were expressed as the mean value of triplicate data points.

## 4. Conclusions

In this study, the secondary metabolites of fungus *Fusarium solani* 8388 isolated from the Shenhu area in the South China Sea were investigated. Seven new polyketide compounds named fusarisolins F-K (**1**–**6**) and fusarin I (**7**), as well as three known analogues (**8**–**10**) were isolated and identified. Fusarisolin I (**1**) comprised both a dichlorobenzene group and an ethylene oxide unit, which was rare in nature. In the in vitro antibacterial bioassays, fusarisolin I (**4**), fusarisolin J (**5**), and 5-deoxybostrycoidin (**9**) exhibited obvious antibacterial activities against methicillin-resistant *Staphylococcus aureus* n315. Fusarisolin H (**3**) and fusarisolin J (**5**) showed inhibitory effects against methicillin-resistant *Staphylococcus aureus* NCTC 10442. With the exception of **5**, all other compounds did not show or showed weak cytotoxicities against human HeLa, A549, and KB cells.

## Figures and Tables

**Table 1:** The ^1^H (400 MHz) and ^13^C (100 MHz) NMR data for compounds **1** and **2** in CDCl_3_.

Position	1		2	
	*δ*_C_, Type	*δ*_H_, mult.	*δ*_C_, Type	*δ*_H_, mult. (*J* in Hz)
1	138.7, C		140.9, C	
2	113.1, C		111.4, C	
3	154.4, C		154.8, C	
4	97.0, CH	6.55, s	96.3, CH	6.52, s
5	155.1, C		154.8, C	
6	115.2, C		111.4, C	
1′	15. 7, CH_3_	1.50, s	29.7, CH_3_	2.11, d (1.4)
2′	64.2, C		148.2, C	
3′	66.4, CH	3.55, s	129.4, CH	6.32, q (1.4)
4′	149.6, C		197.1, C	
5′	124.5, CH	6.39, s	24.3, CH_3_	1.96, s
6′	198.9, C			
7′	32.3, CH_3_	2.25, s		
8′	17.2, CH_3_	2.35, s		
3-OMe	56.7, CH_3_	3.93, s	56.6, CH_3_	3.92, s
5-OMe	56.8, CH_3_	3.93, s	56.6, CH_3_	3.92, s

**Table 2:** The ^1^H and ^13^C NMR data for compounds **3** and **4** in CDCl_3_ at 100 and 400 MHz.

Position	3		4	
	*δ*_C_, Type	*δ*_H_, mult. (*J* in Hz)	*δ*_C_, Type	*δ*_H_, mult. (*J* in Hz)
1	58.5, CH_2_	4.39, dt (19.0, 2.8); 4.71, dd (19.0, 2.8)	58.8, CH_2_	4.39, d (18.9); 4.72, d (18.9)
3	97.2, C		97.2, C	
4	32.5, CH_2_	2.51, dt (18.9, 3.1); 2.86, dd (18.9, 3.3)	32.3, CH_2_	2.51, d (18.8); 2.84, d (18.8)
4a	143.2, C		144.1, C	
5	189.4, C		189.7, C	
5a	114.9, C		115.4, C	
6	152.1, C		152.1, C	
7	154.2, C		153.3, C	
8	115.0, CH	7.05, d (8.3)	119.4, CH	6.83, s
9	120.6, CH	7.63, d (8.3)	136.6, C	
9a	123.8, C		120.6, C	
10	181.6, C		183.2, C	
10a	139.3, C		137.7, C	
3-Me	22.9, CH_3_	1.52, s	22.9, CH_3_	1.53, s
3-OMe	49,0, CH_3_	3.29, s	49.0, CH_3_	3.29, s
7-OMe	56.5, CH_3_	3.98, s	56.4, CH_3_	3.98, s
9-Me			23.4, CH_3_	2.65, s
6-OH		12.45, s		

**Table 3:** The ^1^H and ^13^C NMR data for compound **5** in CDCl_3_ at 100 and 400 MHz.

Position	*δ*_C_, Type	*δ*_H_, mult. (*J* in Hz)	Position	*δ*_C_, Type	*δ*_H_, mult. (*J* in Hz)
1	184.5, C		8	119,2, CH	7.58, overlapped
2	143.9, C		8a	132.2, C	
3	146.7, C		1′	36.2, CH_2_	2.82, d (6.6)
4	190.8, C		2′	67.9, CH	4.07, m
4a	114.9, C		3′	24.4, CH_3_	1.32, d (6.2)
5	161.4, C		2-Me	13.6, CH_3_	2.22, s
6	124.0, CH	7.20, dd (7.7, 1.9)	5-OH		12.10, s
7	136.3, CH	7.57, overlapped			

**Table 4:** Summary of ^13^C (100 MHz) and ^1^H (400 MHz) NMR spectroscopic data for compounds **6** and **7** in CDCl_3_.

Position	6		Position	7	
	*δ*_C_, Type	*δ*_H_, mult. (*J* in Hz)		*δ*_C_, Type	*δ*_H_, mult. (*J* in Hz)
2	164.7, C		1	15.9, CH_3_	1.73, d (7.2)
3	88.1, CH	5.45, d (2.2)	2	140.1, CH	6.96, q (7.2)
4	171.7, C		3	129.9, C	
5	97.7, CH	5.90, d (2.2)	4	85.9, CH	4.26, d (5.4)
6	161.5, C		5	138.9, C	
1′	12.5, CH_3_	1.85, s	6	122.3, CH	6.07, m
2′	123.8, C		7	129.2, CH	6.45, dd (15.4, 10.9)
3′	142.4, CH	6.42, d (9.9)	8	142.3, CH	6.12, d (15.4)
4′	30.9, CH	2.63, dq (9.9, 6.8)	9	142.8, CH	7.12, dd (15.8, 11.1)
5′	44.2, CH_2_	1.31, m	10	131.0, CH	6.16, d (10.2)
6′	32.1, CH	1.31, m	11	198.9, C	
7′	29.2, CH_2_	1.11, m	12	27.4, CH_3_	2.28, s
8′	11.3, CH_3_	0.83, m	13	167.7, C	
9′	20.4, CH_3_	0.97, d (6.5)	14	52.0, CH_3_	3.73, s
10′	19.6, CH_3_	0.85, d (2.9)	15	13.3, CH_3_	1.43, d (1.4)
4-OMe	56.0, CH_3_	3.81, s	16	56.3, CH_3_	3.33, s

**Table 5:** In vitro antibacterial activities (MIC, µg/mL) of **3**, **4**, **5**, and **9** ^a^.

Compounds	3	4	5	9	Amo ^c^	Van ^c^
*Escherichia coli* ATCC 25922	- ^b^	-	-	-	3	50
*Staphylococcus aureus* ATCC 29213	12	25	12	12	3	<0.75
*Staphylococcus aureus* NCTC 10442	6	25	6	12	-	<0.75
*Staphylococcus aureus* n315	12	3	3	6	50	<0.75

^a^ MIC values of compounds **1**, **2**, **6**, **7**, **8** were > 50 µg/mL. ^b^ “-” means MIC value > 50 µg/mL. ^c^ Amoxicillin and vancomycin were used as positive controls.

**Table 6:** In vitro cytotoxic activities (CC_50_, µM, *n* = 3) of **3**, **4**, **5**, and **9** ^a^.

Compounds	HeLa Cells	A549 Cells	Kb Cells
**3**	27.63	- ^b^	34.73
**4**	-	-	-
**5**	9.21	14.02	12.07
**9**	20.33	-	-
Adriamycin ^c^	0.25	0.52	0.11

^a^ CC_50_ values of compounds **1**, **2**, **6**, **7**, **8** were > 100 µM. ^b^ “-” means CC_50_ value > 50 µM. ^c^ Positive control.

## Data Availability

All data supporting the findings of this study are available from the corresponding author upon reasonable request.

## Supplementary Materials

The following supporting information can be downloaded at: https://www.mdpi.com/article/10.3390/jof9090875/s1, Tables S1-1–S1-7: Single crystal X-ray diffraction data of compound **1**; Tables S2-1–S5-2: ECD calculation data of compound **3**, **5**, **6**, **7**; Figures S1-1–S7-6: HRESIMS, UV and NMR spectra of compound **1**–**7**; Figure S8: Phylogenic tree of marine-derived fungus 8388 constructed by MEGA 5.10; Figures S9–S15: High performance liquid chromatography (HPLC) analyses of compounds **1**–**7**; Figure S16: The structure of T5 in the Chinese patent CN202010970367.1.
