# Supplementary material for: Antibacterial Polyketides Isolated from the Marine-Derived Fungus Fusarium solani 8388
Source: J Fungi (Basel). 2023 Aug 24;9(9):875. doi: 10.3390/jof9090875 (PMC10532693; doi:10.3390/jof9090875)
Supplement: Supplementary file 1 [file jof-09-00875-s001.zip › jof-2531213-supplementary.pdf]

# Supporting Information

# Antibacterial polyketides isolated from the marine-derived fungus *Fusarium solani* 8388

**Cankai Lin[1,†], Rongchun Huang[1,†], Juntao Liu[1], Hong Li[1], Liping Zhu[1], Xin Huang[1], Bo Ding[1], Lan Liu[2], Hongbo Huang[1,*] and Yiwen Tao[1,*]**

1 Guangzhou Municipal and Guangdong Provincial Key Laboratory of Molecular Target & Clinical Pharmacology, and the NMPA Laboratory of Respiratory Disease, School of Pharmaceutical Sciences and the Fifth Affiliated Hospital, Guangzhou Medical University, Guangzhou 511436, China; 2020118007@stu.gzhmu.edu.cn (C. L.); 2020118029@stu.gzhmu.edu.cn (R. H.); liujuntao151x@163.com (J.L.); 15211375977@163.com (H.L.); 18955179815 @163.com (L. Z.); 15581460416@163.com (X. H.); dingbo-dmy@gzhmu.edu.cn (B. D.); huanghb@gzhmu.edu.cn (H. H.); yywentao@aliyun.com (Y. T.).

2 School of Marine Sciences, Sun Yat-Sen University, Zhuhai 519082, China; cesllan@mail.sysu.edu.cn (L. L.)

* Correspondence: E-mail: yywentao@aliyun.com (Y. T.); huanghb@gzhmu. edu.cn (H. H.). Tel.: +86-20-37103631 (Y. T.).

† Those authors contributed equally to this work.

# Contents

**Table S1-1 ~ Table S1-7:** Single crystal X-ray diffraction data of compound **1**
**Table S2-1 ~ Table S2-2:** ECD calculation data of compound **3.**
**Table S3-1 ~ Table S3-2:** ECD calculation data of compound **5.**
**Table S4-1 ~ Table S4-4:** ECD calculation data of compound **6.**
**Table S5-1 ~ Table S5-2:** ECD calculation data of compound **7.**
**Figure S1-1 ~ Figure S1-6**: HRESIMS, UV and NMR spectra of compound **1**
**Figure S2-1 ~ Figure S2-8:** HRESIMS, UV and NMR spectra of compound **2**
**Figure S3-1 ~ Figure S3-7:** HRESIMS, UV and NMR spectra of compound **3**
**Figure S4-1 ~ Figure S4-6:** HRESIMS, UV and NMR spectra of compound **4**
**Figure S5-1 ~ Figure S5-7:** HRESIMS, UV and NMR spectra of compound **5**
**Figure S6-1 ~ Figure S6-8:** HRESIMS, UV and NMR spectra of compound **6**
**Figure S7-1 ~ Figure S7-6:** HRESIMS, UV and NMR spectra of compound **7**
**Figure S8.** Phylogenic tree of marine-derived fungus 8388 constructed by MEGA 5.10
**Figure S9-S15** High performance liquid chromatography (HPLC) analyses of compounds **1-7**
**Figure S16** The structure of T5 in the Chinese patent CN202010970367.1 (Dichlororesorcin compound, its preparation method and medical application)

**Table S1-1**. Crystal data and structure refinement for 1 (exp_4472).

| | |
|---|---|
| Identification code | exp_4472 (compound **1**) |
| Empirical formula | $C_{16}H_{18}Cl_2O_4$ |
| Formula weight | 345.20 |
| Temperature/K | 170.00(10) |
| Crystal system | triclinic |
| Space group | P-1 |
| a/Å | 11.5915(3) |
| b/Å | 11.8707(4) |
| c/Å | 12.5216(3) |
| α/° | 105.115(2) |
| β/° | 95.309(2) |
| γ/° | 98.058(2) |
| Volume/Å$^3$ | 1631.85(8) |
| Z | 4 |
| $\rho_{calc}$g/cm$^3$ | 1.405 |
| μ/mm$^{-1}$ | 3.713 |
| F(000) | 720.0 |
| Crystal size/mm$^3$ | 0.14 × 0.12 × 0.11 |
| Radiation | Cu Kα (λ = 1.54184) |
| 2Θ range for data collection/° | 7.38 to 148.062 |
| Index ranges | $-14 \leq h \leq 14$, $-14 \leq k \leq 14$, $-15 \leq l \leq 15$ |
| Reflections collected | 34106 |
| Independent reflections | 6474 [$R_{int}$ = 0.0860, $R_{sigma}$ = 0.0443] |
| Data/restraints/parameters | 6474/0/407 |
| Goodness-of-fit on $F^2$ | 1.086 |
| Final R indexes [I>=2σ (I)] | $R_1$ = 0.0715, $wR_2$ = 0.1766 |
| Final R indexes [all data] | $R_1$ = 0.0771, $wR_2$ = 0.1802 |
| Largest diff. peak/hole / e Å$^{-3}$ | 0.67/-0.38 |

**Table S1-2.** Fractional Atomic Coordinates (×$10^4$) and Equivalent Isotropic Displacement Parameters (Å$^2$×$10^3$) for exp_4472. $U_{eq}$ is defined as 1/3 of the trace of the orthogonalised $U_{IJ}$ tensor.

| **Atom** | ***x*** | ***y*** | ***z*** | **U(eq)** |
|---|---|---|---|---|
| Cl1 | 4402.6(8) | 4216.1(8) | 1144.6(7) | 41.8(2) |
| Cl2 | 5023.8(9) | 8215.3(8) | 4585.5(7) | 43.6(2) |
| O1 | 3890(3) | 6721(3) | 5734(2) | 46.3(7) |
| O2 | 3267(3) | 3141(2) | 2648(2) | 48.3(7) |
| O3 | 4588(2) | 6741(2) | 1117.4(19) | 36.6(5) |
| O4 | 6670(4) | 10918(3) | 1226(3) | 69.7(10) |
| C1 | 3941(3) | 6091(4) | 4671(3) | 37.9(8) |
| C2 | 3506(3) | 4892(4) | 4226(3) | 40.2(8) |
| C3 | 3632(3) | 4322(3) | 3134(3) | 36.6(8) |
| C4 | 4174(3) | 4966(3) | 2478(3) | 33.8(7) |
| C5 | 4589(3) | 6177(3) | 2892(3) | 31.5(7) |
| C6 | 4470(3) | 6717(3) | 4002(3) | 34.2(7) |
| C7 | 5196(3) | 6883(3) | 2211(3) | 31.5(7) |
| C8 | 6505(3) | 6909(3) | 2280(3) | 40.2(8) |
| C9 | 4630(3) | 7841(3) | 1947(3) | 34.2(7) |
| C10 | 5286(3) | 8937(3) | 1794(3) | 37.5(8) |
| C11 | 5773(4) | 8909(3) | 859(3) | 40.8(8) |
| C12 | 6456(4) | 9922(4) | 591(3) | 48.3(10) |
| C13 | 6879(4) | 9654(4) | -525(4) | 56.8(11) |
| C14 | 5313(4) | 10023(4) | 2750(3) | 49.7(10) |
| C15 | 2758(4) | 2452(4) | 3315(4) | 55.3(11) |
| C16 | 3292(4) | 6132(5) | 6439(3) | 54.2(11) |
| Cl3 | 1549.7(8) | 8532.0(7) | 2420.8(7) | 39.5(2) |
| Cl4 | 230.5(8) | 4308.0(7) | 3263.3(7) | 39.0(2) |
| O5 | 2032(2) | 7117(2) | 367(2) | 38.7(6) |
| O6 | 853(3) | 3344(2) | 1096(2) | 42.6(6) |
| O7 | 1241(2) | 6657(2) | 4829.9(19) | 34.5(5) |
| O8 | 1297(3) | 10710(2) | 7686(2) | 54.9(8) |
| C17 | 987(3) | 6373(3) | 2784(3) | 29.3(7) |
| C18 | 1390(3) | 6997(3) | 2052(3) | 29.9(7) |
| C19 | 1653(3) | 6421(3) | 1002(3) | 32.0(7) |
| C20 | 1495(3) | 5192(3) | 678(3) | 33.1(7) |
| C21 | 1066(3) | 4545(3) | 1376(3) | 33.2(7) |
| C22 | 812(3) | 5137(3) | 2419(3) | 30.9(7) |
| C23 | 2350(4) | 6550(4) | -701(3) | 44.8(9) |
| C24 | 1111(4) | 2714(3) | 32(3) | 44.0(9) |
| C25 | -558(3) | 7158(3) | 3936(3) | 39.3(8) |
| C26 | 701(3) | 7005(3) | 3909(3) | 29.3(7) |
| C27 | 1674(3) | 7810(3) | 4720(3) | 31.2(7) |

**Table S1-2.** Fractional Atomic Coordinates (×10$^4$) and Equivalent Isotropic Displacement Parameters (Å$^2$×10$^3$) for exp_4472. $U_{eq}$ is defined as 1/3 of the trace of the orthogonalised $U_{IJ}$ tensor.

| Atom | *x* | *y* | *z* | U(eq) |
|---|---|---|---|---|
| C28 | 1511(3) | 8860(3) | 5610(3) | 34.5(7) |
| C29 | 1147(4) | 8719(3) | 6551(3) | 40.4(8) |
| C30 | 964(4) | 9655(3) | 7543(3) | 41.9(8) |
| C31 | 367(4) | 9205(4) | 8396(3) | 55.1(11) |
| C32 | 1833(4) | 10015(3) | 5336(4) | 50.3(10) |

**Table S1-3.** Anisotropic Displacement Parameters (Å$^2$×10$^3$) for exp_4472. The Anisotropic displacement factor exponent takes the form: $-2\pi^2[h^2a^{*2}U_{11}+2hka^*b^*U_{12}+\ldots]$.

| Atom | $U_{11}$ | $U_{22}$ | $U_{33}$ | $U_{23}$ | $U_{13}$ | $U_{12}$ |
|---|---|---|---|---|---|---|
| Cl1 | 48.7(5) | 39.7(5) | 33.3(4) | 2.2(3) | 11.1(4) | 6.9(4) |
| Cl2 | 51.0(5) | 45.1(5) | 28.3(4) | 1.8(3) | 7.4(4) | 1.0(4) |
| O1 | 52.2(16) | 60.8(17) | 27.2(12) | 12.5(12) | 13.2(11) | 8.5(13) |
| O2 | 52.9(17) | 42.4(15) | 48.5(16) | 14.4(12) | 9.3(13) | -0.1(12) |
| O3 | 45.5(14) | 36.9(13) | 25.0(11) | 4.9(9) | 3.0(10) | 6.7(10) |
| O4 | 103(3) | 43.1(17) | 65(2) | 21.5(16) | 19.3(19) | 1.2(17) |
| C1 | 33.6(18) | 55(2) | 26.1(16) | 11.6(15) | 3.6(14) | 9.8(16) |
| C2 | 35.4(18) | 55(2) | 34.9(18) | 20.4(17) | 8.0(15) | 6.7(16) |
| C3 | 32.2(17) | 38.2(18) | 40.0(19) | 11.6(15) | 5.1(14) | 6.3(14) |
| C4 | 33.0(17) | 39.5(18) | 27.2(16) | 5.6(14) | 4.8(13) | 7.3(14) |
| C5 | 27.2(16) | 40.3(18) | 27.4(16) | 8.6(14) | 5.9(13) | 7.1(13) |
| C6 | 31.6(17) | 40.1(18) | 28.5(16) | 6.0(14) | 3.8(13) | 4.9(14) |
| C7 | 30.9(17) | 38.2(17) | 23.8(15) | 4.7(13) | 5.6(13) | 6.6(13) |
| C8 | 33.9(18) | 45(2) | 45(2) | 14.2(16) | 11.8(16) | 9.1(15) |
| C9 | 34.6(18) | 41.3(19) | 24.7(15) | 3.9(13) | 5.8(13) | 8.3(14) |
| C10 | 43(2) | 38.6(18) | 29.9(17) | 7.1(14) | 0.4(15) | 11.5(15) |
| C11 | 49(2) | 39.2(19) | 35.7(18) | 10.0(15) | 7.9(16) | 11.9(16) |
| C12 | 57(2) | 51(2) | 44(2) | 23.0(19) | 6.7(18) | 14.3(19) |
| C13 | 62(3) | 68(3) | 55(3) | 32(2) | 22(2) | 20(2) |
| C14 | 70(3) | 41(2) | 36(2) | 6.6(16) | 6.0(19) | 10.4(19) |
| C15 | 59(3) | 49(2) | 62(3) | 24(2) | 10(2) | 4(2) |
| C16 | 57(3) | 84(3) | 35(2) | 30(2) | 20.0(18) | 22(2) |
| Cl3 | 53.2(5) | 33.0(4) | 37.5(4) | 13.0(3) | 16.5(4) | 12.2(4) |
| Cl4 | 49.6(5) | 33.4(4) | 34.9(4) | 11.3(3) | 12.9(4) | 1.8(3) |
| O5 | 49.1(15) | 42.5(13) | 30.1(12) | 14.7(10) | 15.7(11) | 11.4(11) |
| O6 | 58.4(17) | 30.3(12) | 35.5(13) | 1.9(10) | 14.5(12) | 3.6(11) |
| O7 | 45.1(14) | 31.5(12) | 27.6(11) | 8.8(9) | 4.8(10) | 8.0(10) |
| O8 | 72(2) | 36.1(15) | 50.2(16) | -0.7(12) | 23.0(15) | 2.2(13) |
| C17 | 26.4(15) | 34.5(16) | 27.7(15) | 7.9(13) | 4.8(12) | 8.0(13) |

**Table S1-3.** Anisotropic Displacement Parameters (Å$^2$×10$^3$) for exp_4472. The Anisotropic displacement factor exponent takes the form: $-2\pi^2[h^2a^{*2}U_{11}+2hka^*b^*U_{12}+\ldots]$.

| Atom | $U_{11}$ | $U_{22}$ | $U_{33}$ | $U_{23}$ | $U_{13}$ | $U_{12}$ |
|---|---|---|---|---|---|---|
| C18 | 31.1(16) | 31.0(16) | 30.4(16) | 10.4(13) | 5.9(13) | 9.9(13) |
| C19 | 29.7(16) | 41.8(18) | 28.1(16) | 13.8(14) | 6.6(13) | 8.3(14) |
| C20 | 35.3(17) | 38.7(18) | 25.3(15) | 6.2(13) | 8.5(13) | 8.3(14) |
| C21 | 32.4(17) | 33.6(17) | 30.7(16) | 4.4(13) | 4.7(13) | 4.7(13) |
| C22 | 33.8(17) | 32.4(16) | 26.9(15) | 8.4(13) | 6.2(13) | 5.6(13) |
| C23 | 55(2) | 53(2) | 27.9(17) | 13.0(16) | 14.1(16) | 5.5(18) |
| C24 | 52(2) | 36.8(19) | 38.1(19) | -1.0(15) | 10.1(17) | 8.9(16) |
| C25 | 32.9(18) | 44(2) | 41.4(19) | 7.6(16) | 13.8(15) | 8.6(15) |
| C26 | 32.5(16) | 30.8(16) | 27.5(15) | 11.2(13) | 8.0(13) | 6.5(13) |
| C27 | 30.1(16) | 36.3(17) | 30.3(16) | 12.6(13) | 8.6(13) | 6.4(13) |
| C28 | 34.1(17) | 34.3(17) | 33.3(17) | 7.6(14) | 4.5(14) | 3.5(14) |
| C29 | 50(2) | 31.0(17) | 37.1(19) | 4.0(14) | 11.1(16) | 4.3(15) |
| C30 | 44(2) | 41(2) | 36.5(19) | 3.6(15) | 9.7(16) | 2.8(16) |
| C31 | 68(3) | 52(2) | 37(2) | -0.9(18) | 23(2) | -4(2) |
| C32 | 72(3) | 34.6(19) | 44(2) | 9.4(16) | 18(2) | 4.8(19) |

**Table S1-4.** Bond Lengths for exp_4472.

| Atom | Atom | Length/Å | Atom | Atom | Length/Å |
|---|---|---|---|---|---|
| Cl1 | C4 | 1.739(3) | Cl3 | C18 | 1.737(3) |
| Cl2 | C6 | 1.739(4) | Cl4 | C22 | 1.740(3) |
| O1 | C1 | 1.357(4) | O5 | C19 | 1.347(4) |
| O1 | C16 | 1.431(5) | O5 | C23 | 1.438(4) |
| O2 | C3 | 1.362(4) | O6 | C21 | 1.357(4) |
| O2 | C15 | 1.424(5) | O6 | C24 | 1.428(4) |
| O3 | C7 | 1.438(4) | O7 | C26 | 1.442(4) |
| O3 | C9 | 1.433(4) | O7 | C27 | 1.436(4) |
| O4 | C12 | 1.217(5) | O8 | C30 | 1.218(5) |
| C1 | C2 | 1.388(6) | C17 | C18 | 1.394(5) |
| C1 | C6 | 1.386(5) | C17 | C22 | 1.397(5) |
| C2 | C3 | 1.390(5) | C17 | C26 | 1.504(4) |
| C3 | C4 | 1.394(5) | C18 | C19 | 1.398(5) |
| C4 | C5 | 1.392(5) | C19 | C20 | 1.390(5) |
| C5 | C6 | 1.399(5) | C20 | C21 | 1.388(5) |
| C5 | C7 | 1.497(5) | C21 | C22 | 1.392(5) |
| C7 | C8 | 1.508(5) | C25 | C26 | 1.498(5) |
| C7 | C9 | 1.484(5) | C26 | C27 | 1.482(5) |
| C9 | C10 | 1.479(5) | C27 | C28 | 1.487(5) |
| C10 | C11 | 1.341(5) | C28 | C29 | 1.330(5) |

**Table S1-4.** Bond Lengths for exp_4472.

| Atom | Atom | Length/Å | Atom | Atom | Length/Å |
|---|---|---|---|---|---|
| C10 | C14 | 1.508(5) | C28 | C32 | 1.504(5) |
| C11 | C12 | 1.477(6) | C29 | C30 | 1.489(5) |
| C12 | C13 | 1.495(6) | C30 | C31 | 1.496(6) |

**Table S1-5.** Bond Angles for exp_4472.

| Atom | Atom | Atom | Angle/° | Atom | Atom | Atom | Angle/° |
|---|---|---|---|---|---|---|---|
| C1 | O1 | C16 | 118.3(3) | C19 | O5 | C23 | 117.6(3) |
| C3 | O2 | C15 | 117.6(3) | C21 | O6 | C24 | 118.0(3) |
| C9 | O3 | C7 | 62.2(2) | C27 | O7 | C26 | 62.0(2) |
| O1 | C1 | C2 | 124.7(3) | C18 | C17 | C22 | 117.7(3) |
| O1 | C1 | C6 | 116.1(3) | C18 | C17 | C26 | 121.2(3) |
| C6 | C1 | C2 | 119.1(3) | C22 | C17 | C26 | 121.0(3) |
| C1 | C2 | C3 | 120.2(3) | C17 | C18 | Cl3 | 120.2(3) |
| O2 | C3 | C2 | 123.9(3) | C17 | C18 | C19 | 121.8(3) |
| O2 | C3 | C4 | 116.4(3) | C19 | C18 | Cl3 | 118.0(3) |
| C2 | C3 | C4 | 119.7(3) | O5 | C19 | C18 | 116.5(3) |
| C3 | C4 | Cl1 | 118.7(3) | O5 | C19 | C20 | 124.7(3) |
| C5 | C4 | Cl1 | 119.8(3) | C20 | C19 | C18 | 118.9(3) |
| C5 | C4 | C3 | 121.3(3) | C21 | C20 | C19 | 120.7(3) |
| C4 | C5 | C6 | 117.4(3) | O6 | C21 | C20 | 123.6(3) |
| C4 | C5 | C7 | 122.2(3) | O6 | C21 | C22 | 116.9(3) |
| C6 | C5 | C7 | 120.2(3) | C20 | C21 | C22 | 119.5(3) |
| C1 | C6 | Cl2 | 118.2(3) | C17 | C22 | Cl4 | 119.8(3) |
| C1 | C6 | C5 | 122.2(3) | C21 | C22 | Cl4 | 118.7(3) |
| C5 | C6 | Cl2 | 119.6(3) | C21 | C22 | C17 | 121.4(3) |
| O3 | C7 | C5 | 115.4(3) | O7 | C26 | C17 | 114.6(3) |
| O3 | C7 | C8 | 115.7(3) | O7 | C26 | C25 | 115.9(3) |
| O3 | C7 | C9 | 58.7(2) | O7 | C26 | C27 | 58.8(2) |
| C5 | C7 | C8 | 114.7(3) | C25 | C26 | C17 | 114.9(3) |
| C9 | C7 | C5 | 117.7(3) | C27 | C26 | C17 | 117.7(3) |
| C9 | C7 | C8 | 122.4(3) | C27 | C26 | C25 | 122.5(3) |
| O3 | C9 | C7 | 59.1(2) | O7 | C27 | C26 | 59.2(2) |
| O3 | C9 | C10 | 119.1(3) | O7 | C27 | C28 | 117.9(3) |
| C10 | C9 | C7 | 123.9(3) | C26 | C27 | C28 | 124.0(3) |
| C9 | C10 | C14 | 113.9(3) | C27 | C28 | C32 | 113.6(3) |
| C11 | C10 | C9 | 120.2(3) | C29 | C28 | C27 | 119.9(3) |
| C11 | C10 | C14 | 125.8(4) | C29 | C28 | C32 | 126.5(3) |
| C10 | C11 | C12 | 126.4(4) | C28 | C29 | C30 | 127.8(4) |
| O4 | C12 | C11 | 123.1(4) | O8 | C30 | C29 | 123.9(4) |

**Table S1-5.** Bond Angles for exp_4472.

| Atom | Atom | Atom | Angle/° | Atom | Atom | Atom | Angle/° |
|---|---|---|---|---|---|---|---|
| O4 | C12 | C13 | 121.3(4) | O8 | C30 | C31 | 121.3(4) |
| C11 | C12 | C13 | 115.6(4) | C29 | C30 | C31 | 114.8(3) |

**Table S1-6.** Torsion Angles for exp_4472.

| A | B | C | D | Angle/° | A | B | C | D | Angle/° |
|---|---|---|---|---|---|---|---|---|---|
| Cl1 | C4 | C5 | C6 | -174.6(3) | Cl3 | C18 | C19 | O5 | 1.2(4) |
| Cl1 | C4 | C5 | C7 | 2.3(5) | Cl3 | C18 | C19 | C20 | -178.0(3) |
| O1 | C1 | C2 | C3 | -178.2(3) | O5 | C19 | C20 | C21 | -178.1(3) |
| O1 | C1 | C6 | Cl2 | 0.8(4) | O6 | C21 | C22 | Cl4 | -0.9(4) |
| O1 | C1 | C6 | C5 | 179.6(3) | O6 | C21 | C22 | C17 | -179.3(3) |
| O2 | C3 | C4 | Cl1 | -3.2(4) | O7 | C26 | C27 | C28 | -104.7(3) |
| O2 | C3 | C4 | C5 | -179.6(3) | O7 | C27 | C28 | C29 | 10.3(5) |
| O3 | C7 | C9 | C10 | -106.2(4) | O7 | C27 | C28 | C32 | -171.6(3) |
| O3 | C9 | C10 | C11 | 4.0(5) | C17 | C18 | C19 | O5 | 179.9(3) |
| O3 | C9 | C10 | C14 | -177.1(3) | C17 | C18 | C19 | C20 | 0.7(5) |
| C1 | C2 | C3 | O2 | 177.7(3) | C17 | C26 | C27 | O7 | -103.4(3) |
| C1 | C2 | C3 | C4 | -1.5(5) | C17 | C26 | C27 | C28 | 151.9(3) |
| C2 | C1 | C6 | Cl2 | -179.2(3) | C18 | C17 | C22 | Cl4 | -176.3(2) |
| C2 | C1 | C6 | C5 | -0.5(5) | C18 | C17 | C22 | C21 | 2.0(5) |
| C2 | C3 | C4 | Cl1 | 176.1(3) | C18 | C17 | C26 | O7 | -129.5(3) |
| C2 | C3 | C4 | C5 | -0.3(5) | C18 | C17 | C26 | C25 | 92.6(4) |
| C3 | C4 | C5 | C6 | 1.7(5) | C18 | C17 | C26 | C27 | -63.3(4) |
| C3 | C4 | C5 | C7 | 178.7(3) | C18 | C19 | C20 | C21 | 1.0(5) |
| C4 | C5 | C6 | Cl2 | 177.4(3) | C19 | C20 | C21 | O6 | 177.6(3) |
| C4 | C5 | C6 | C1 | -1.3(5) | C19 | C20 | C21 | C22 | -1.2(5) |
| C4 | C5 | C7 | O3 | 50.8(4) | C20 | C21 | C22 | Cl4 | 178.0(3) |
| C4 | C5 | C7 | C8 | -87.5(4) | C20 | C21 | C22 | C17 | -0.4(5) |
| C4 | C5 | C7 | C9 | 117.3(4) | C22 | C17 | C18 | Cl3 | 176.5(2) |
| C5 | C7 | C9 | O3 | -104.4(3) | C22 | C17 | C18 | C19 | -2.2(5) |
| C5 | C7 | C9 | C10 | 149.3(3) | C22 | C17 | C26 | O7 | 53.1(4) |
| C6 | C1 | C2 | C3 | 1.9(5) | C22 | C17 | C26 | C25 | -84.8(4) |
| C6 | C5 | C7 | O3 | -132.3(3) | C22 | C17 | C26 | C27 | 119.4(3) |
| C6 | C5 | C7 | C8 | 89.3(4) | C23 | O5 | C19 | C18 | 177.8(3) |
| C6 | C5 | C7 | C9 | -65.9(4) | C23 | O5 | C19 | C20 | -3.1(5) |
| C7 | O3 | C9 | C10 | 114.3(4) | C24 | O6 | C21 | C20 | 1.5(5) |
| C7 | C5 | C6 | Cl2 | 0.4(4) | C24 | O6 | C21 | C22 | -179.7(3) |
| C7 | C5 | C6 | C1 | -178.3(3) | C25 | C26 | C27 | O7 | 102.7(3) |
| C7 | C9 | C10 | C11 | 74.4(5) | C25 | C26 | C27 | C28 | -2.0(5) |
| C7 | C9 | C10 | C14 | -106.7(4) | C26 | O7 | C27 | C28 | 114.9(3) |

**Table S1-6.** Torsion Angles for exp_4472.

| A | B | C | D | Angle/° | A | B | C | D | Angle/° |
|---|---|---|---|---|---|---|---|---|---|
| C8 | C7 | C9 | O3 | 102.4(3) | C26 | C17 | C18 | Cl3 | -1.0(4) |
| C8 | C7 | C9 | C10 | -3.8(5) | C26 | C17 | C18 | C19 | -179.6(3) |
| C9 | O3 | C7 | C5 | 108.3(3) | C26 | C17 | C22 | Cl4 | 1.2(4) |
| C9 | O3 | C7 | C8 | -113.8(3) | C26 | C17 | C22 | C21 | 179.5(3) |
| C9 | C10 | C11 | C12 | -179.6(4) | C26 | C27 | C28 | C29 | 80.3(5) |
| C10 | C11 | C12 | O4 | 1.4(7) | C26 | C27 | C28 | C32 | -101.5(4) |
| C10 | C11 | C12 | C13 | -178.6(4) | C27 | O7 | C26 | C17 | 108.7(3) |
| C14 | C10 | C11 | C12 | 1.7(7) | C27 | O7 | C26 | C25 | -113.9(3) |
| C15 | O2 | C3 | C2 | -1.9(5) | C27 | C28 | C29 | C30 | 178.2(4) |
| C15 | O2 | C3 | C4 | 177.3(3) | C28 | C29 | C30 | O8 | -11.3(7) |
| C16 | O1 | C1 | C2 | -3.5(5) | C28 | C29 | C30 | C31 | 170.7(4) |
| C16 | O1 | C1 | C6 | 176.4(3) | C32 | C28 | C29 | C30 | 0.3(7) |

**Table S1-7.** Hydrogen Atom Coordinates (Å×$10^4$) and Isotropic Displacement Parameters (Å$^2$×$10^3$) for exp_4472.

| Atom | *x* | *y* | *z* | U(eq) |
|---|---|---|---|---|
| H2 | 3120.31 | 4458.62 | 4669.62 | 48 |
| H8A | 6661.78 | 6127.06 | 1889.87 | 60 |
| H8B | 6851.97 | 7118.23 | 3064.5 | 60 |
| H8C | 6853.61 | 7499.66 | 1929.41 | 60 |
| H9 | 3876 | 7938.7 | 2261.55 | 41 |
| H11 | 5668.13 | 8162.6 | 315.53 | 49 |
| H13A | 7259.09 | 10390.33 | -651.83 | 85 |
| H13B | 6210.12 | 9284.47 | -1108.49 | 85 |
| H13C | 7446.07 | 9111.18 | -546.81 | 85 |
| H14A | 4580.48 | 9953.19 | 3079.07 | 74 |
| H14B | 5395.4 | 10727.49 | 2474.84 | 74 |
| H14C | 5981.22 | 10095.91 | 3318.12 | 74 |
| H15A | 2525.2 | 1625.73 | 2866.87 | 83 |
| H15B | 2064.32 | 2759.72 | 3582.17 | 83 |
| H15C | 3334.3 | 2496.09 | 3954.94 | 83 |
| H16A | 2477.94 | 5809.8 | 6092.36 | 81 |
| H16B | 3295.2 | 6696.88 | 7166.43 | 81 |
| H16C | 3691.71 | 5484.71 | 6539.55 | 81 |
| H20 | 1682.75 | 4790.05 | -28.97 | 40 |
| H23A | 1650.08 | 6057.48 | -1186.23 | 67 |
| H23B | 2935.89 | 6052.84 | -600.79 | 67 |
| H23C | 2682.06 | 7154.26 | -1044.45 | 67 |
| H24A | 641.63 | 2920.47 | -558.76 | 66 |
| H24B | 918.35 | 1860.06 | -61.08 | 66 |

**Table S1-7.** Hydrogen Atom Coordinates (Å×$10^4$) and Isotropic Displacement Parameters (Å$^2$×$10^3$) for exp_4472.

| Atom | *x* | *y* | *z* | U(eq) |
|---|---|---|---|---|
| H24C | 1948.5 | 2928.17 | -14.56 | 66 |
| H25A | -1062.66 | 6378.02 | 3720.94 | 59 |
| H25B | -772.3 | 7604.63 | 3413.22 | 59 |
| H25C | -662.26 | 7589.89 | 4692.77 | 59 |
| H27 | 2450.54 | 7883.97 | 4434.39 | 37 |
| H29 | 982.72 | 7926.98 | 6594.92 | 48 |
| H31A | 919.23 | 8864.49 | 8809.03 | 83 |
| H31B | -313.34 | 8594.46 | 8022.23 | 83 |
| H31C | 104.02 | 9859.86 | 8915.31 | 83 |
| H32A | 2563.89 | 10016.27 | 5000.12 | 75 |
| H32B | 1943.28 | 10669.65 | 6020.44 | 75 |
| H32C | 1200.6 | 10110.52 | 4807.33 | 75 |

**Table S2-1.** B3LYP-D3/def2-TZVP/PCM energies (*E*), relative thermol energies (Δ*E*), relative free energies (Δ*G*), and equilibrium populations (P)[a] of low-energy conformers of 3*S*-**3**.

| Conformer of (**3**) | *E* (Hartree) | Δ*E* (kcal/mol) | Δ*G* (kcal/mol) | P (%) |
|---|---|---|---|---|
| **a** | -1070.6940687 | 0.002761044 | 0.0 | 48.89 |
| **b** | -1070.6846998 | 5.8818394883 | 3.868912905 | 0.00 |
| **c** | -1070.6901952 | 2.433421029 | 2.787273918 | 0.81 |
| **d** | -1070.6845347 | 5.985441384 | 4.105233171 | 0.00 |
| **e** | -1070.688503 | 3.495293451 | 1.061182161 | 0.13 |
| **f** | -1070.6885056 | 3.493661925 | 1.061182159 | 0.13 |
| **g** | -1070.6889318 | 3.226217163 | 3.70921161 | 0.21 |
| **h** | -1070.690067 | 2.513867811 | 3.023782437 | 0.70 |
| **i** | -1070.6940731 | 0.0 | 0.0 | 49.12 |

[a] From Δ*G* values at 298.15 K.

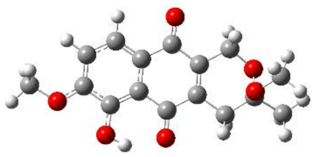
a

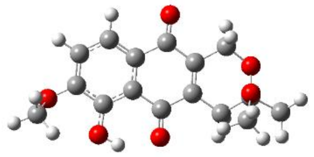
b

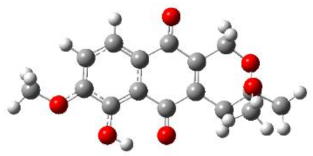
c

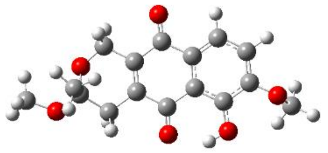
d

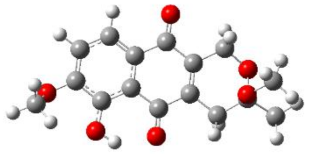
e

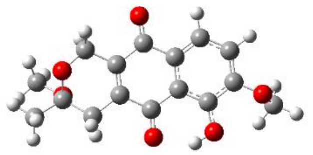
f

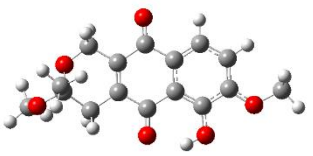
g

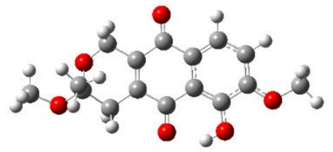
h

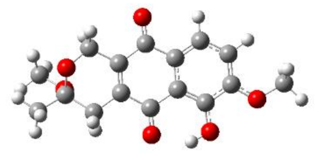
i

**Table S2-2.** B3LYP-D3/def2-TZVP/PCM energies (*E*), relative thermol energies (Δ*E*), relative free energies (Δ*G*), and equilibrium populations (P)[a] of low-energy conformers of 3*R*-**3**.

| Conformer of (**3**) | *E* (Hartree) | Δ*E* (kcal/mol) | Δ*G* (kcal/mol) | P (%) |
|---|---|---|---|---|
| **a** | -1070.688507 | 5.649849036 | 1.061182161 | 0.14 |
| **b** | -1070.6940692 | 0.002761044 | 0.000062751 | 49.33 |
| **c** | -1070.6847009 | 5.881462977 | 3.868912905 | 0.00 |
| **d** | -1070.6884359 | 3.537713127 | 2.787336669 | 0.13 |
| **e** | -1070.6845021 | 6.006211865 | 4.105233171 | 0.00 |
| **f** | -1070.6885405 | 3.472075581 | 1.055848326 | 0.14 |
| **g** | -1070.68833969 | 3.598085641 | 4.778990658 | 0.00 |
| **h** | -1070.690067 | 2.514181566 | 3.02378437 | 0.71 |
| **i** | -1070.6940736 | 0.0 | 0.0 | 49.56 |

[a] From Δ*G* values at 298.15 K.

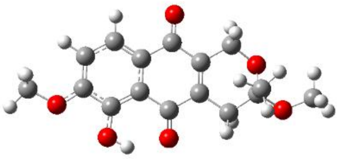

a

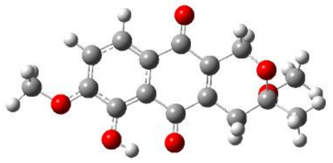

b

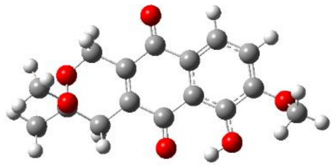

c

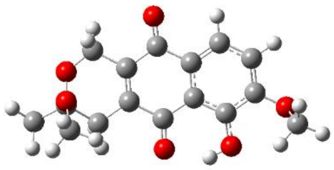

d

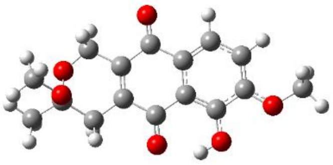

e

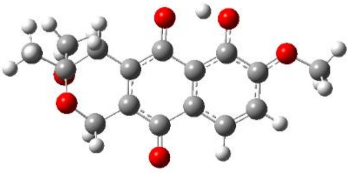

f

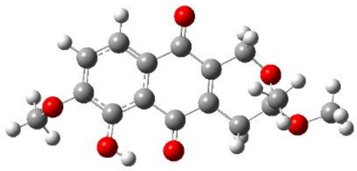

g

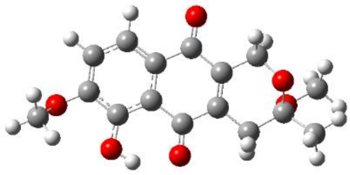

h

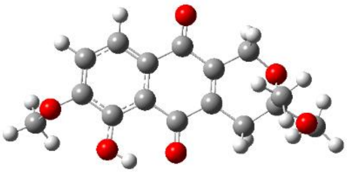

i

**Table S3-1.** B3LYP-D3/def2-TZVP/PCM energies (*E*), relative thermol energies (Δ*E*), relative free energies (Δ*G*), and equilibrium populations (P)[a] of low-energy conformers of 2'*S*-**5**.

| Conformer of (**5**) | *E* (Hartree) | Δ*E* (kcal/mol) | Δ*G* (kcal/mol) | P (%) |
|---|---|---|---|---|
| **a** | -842.8538091 | 0.915160584 | 0.381337827 | 14.16 |
| **b** | -842.8552675 | 0.0 | 0.0 | 66.46 |
| **c** | -842.8525725 | 1.69113945 | 1.009726341 | 3.82 |
| **d** | -842.8537951 | 0.923945724 | 0.938001948 | 13.95 |
| **e** | -842.8480088 | 4.554906837 | 4.042607673 | 0.03 |
| **f** | -842.8517414 | 2.468373336 | 2.30874792 | 1.58 |

[a] From Δ*G* values at 298.15 K.

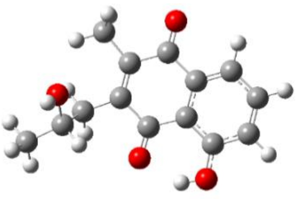

a

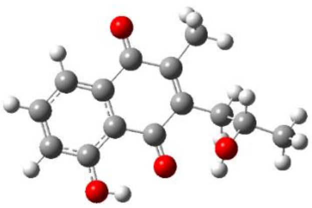

b

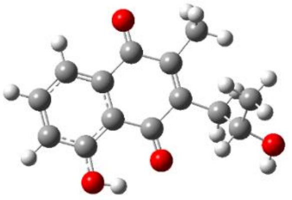

c

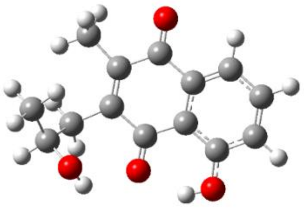

d

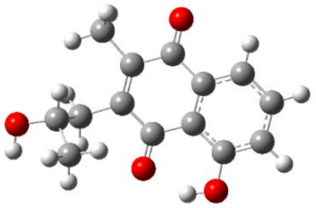

e

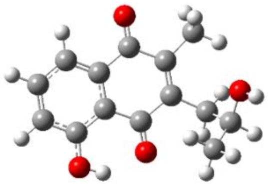

f

**Table S3-2.** B3LYP-D3/def2-TZVP/PCM energies (*E*), relative thermol energies (Δ*E*), relative free energies (Δ*G*), and equilibrium populations (P)[a] of low-energy conformers of 2'*R*-**5**.

| Conformer of (**5**) | *E* (Hartree) | Δ*E* (kcal/mol) | Δ*G* (kcal/mol) | P (%) |
|---|---|---|---|---|
| **a** | -842.8538045 | 0.917733375 | 0.381337827 | 13.20 |
| **b** | -842.855267 | 0.0 | 0.0 | 62.20 |
| **c** | -842.8525723 | 1.59638544 | 1.288152528 | 3.27 |
| **d** | -842.8537952 | 0.923569218 | 1,21665384 | 13.07 |
| **e** | -842.8531323 | 1.339545597 | 2.221868583 | 6.47 |
| **f** | -842.8517444 | 2.210466726 | 2.30874792 | 1.49 |

[a] From Δ*G* values at 298.15 K.

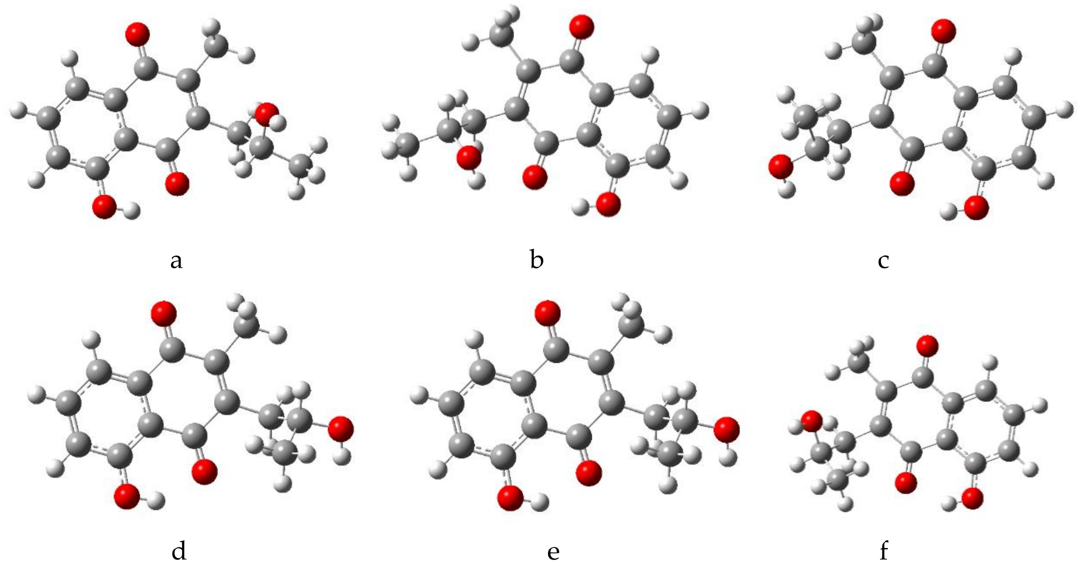

**Table S4-1.** B3LYP-D3/def2-TZVP/PCM energies (*E*), relative thermol energies (Δ*E*), relative free energies (Δ*G*), and equilibrium populations (P)[a] of low-energy conformers of 4'*S*, 6'*S*-**6**.

| Conformer of (**6**) | *E* (Hartree) | Δ*E* (kcal/mol) | Δ*G* (kcal/mol) | P (%) |
|---|---|---|---|---|
| **a** | -849.8484857 | 2.199924558 | 3.0380896665 | 1.18 |
| **b** | -849.8487374 | 2.041980291 | 2.874748812 | 1.54 |
| **c** | -849.8474571 | 2.845381344 | 3.637612719 | 0.40 |
| **d** | -849.8517246 | 0.167482419 | 0.197351895 | 36.44 |
| **e** | -849.850685 | 0.819841815 | 0.798882981 | 12.10 |
| **f** | -849.8519915 | 0.0 | 0.0 | 48.35 |

[a] From Δ*G* values at 298.15 K.

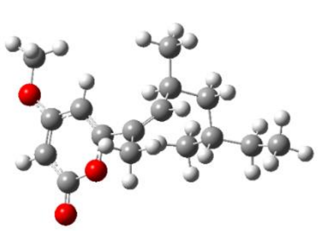

a

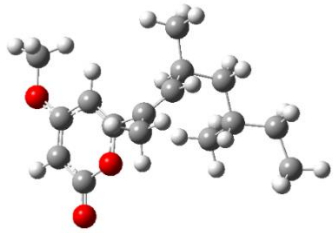

b

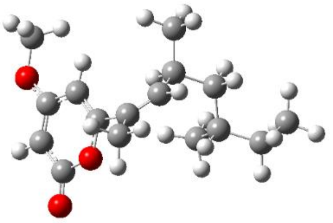

c

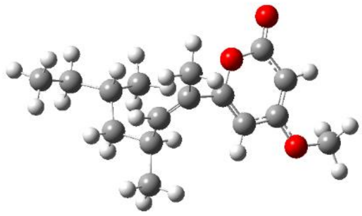

d

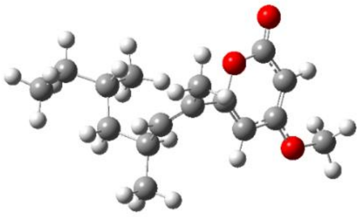

e

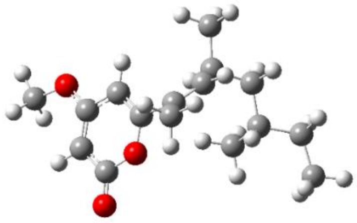

f

**Table S4-2.** B3LYP-D3/def2-TZVP/PCM energies (*E*), relative thermol energies (Δ*E*), relative free energies (Δ*G*), and equilibrium populations (P)[a] of low-energy conformers of 4'*R*,6'*R*-**6**.

| Conformer of (**6**) | *E* (Hartree) | Δ*E* (kcal/mol) | Δ*G* (kcal/mol) | P (%) |
|---|---|---|---|---|
| **a** | -849.8484834 | 1.990336218 | 2.845569597 | 2.24 |
| **b** | -849.8475649 | 2.56856683 | 3.415976187 | 0.84 |
| **c** | - 849.8516552 | 0.0 | 0.0 | 64.56 |
| **d** | -849.8507756 | 0.551957796 | 0.563252976 | 25.41 |
| **e** | -849.8465115 | 3.227723187 | 3.785705079 | 0.28 |
| **f** | -849.8495138 | 1.343749914 | 3.543046962 | 6.67 |

[a] From Δ*G* values at 298.15 K.

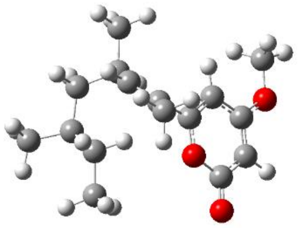

a

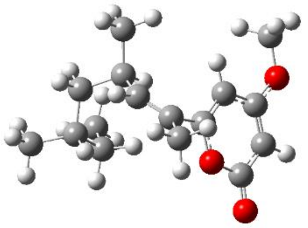

b

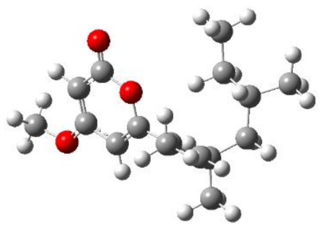

c

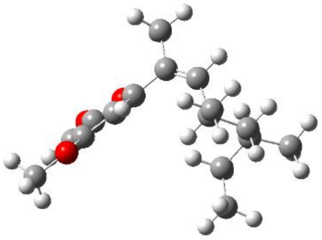

d

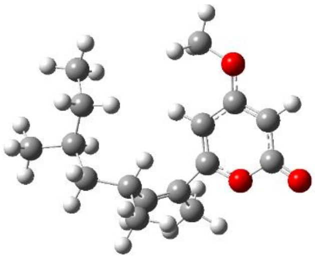

e

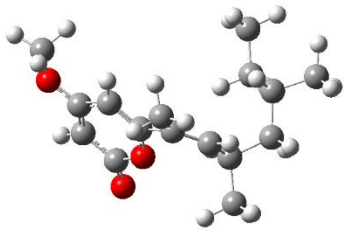

f

**Table S4-3.** B3LYP-D3/def2-TZVP/PCM energies (*E*), relative thermol energies (Δ*E*), relative free energies (Δ*G*), and equilibrium populations (P)[a] of low-energy conformers of 4'*R*,6'*S*-**6**.

| Conformer of (**6**) | *E* (Hartree) | Δ*E* (kcal/mol) | Δ*G* (kcal/mol) | P (%) |
|---|---|---|---|---|
| **a** | -849.8484834 | 1.990336218 | 2.845569597 | 2.24 |
| **b** | -849.8475649 | 2.566704153 | 3.415976187 | 0.84 |
| **c** | -849.8516552 | 0.0 | 0.0 | 64.56 |
| **d** | -849.8507756 | 0.551957796 | 0.563252976 | 25.41 |
| **e** | -849.8465115 | 3.227723187 | 3.785705079 | 0.28 |
| **f** | -849.8495138 | 1.343749914 | 3.543046962 | 6.67 |

[a] From Δ*G* values at 298.15 K.

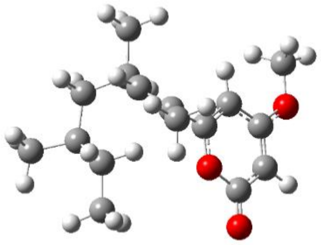

a

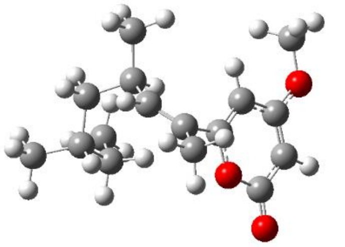

b

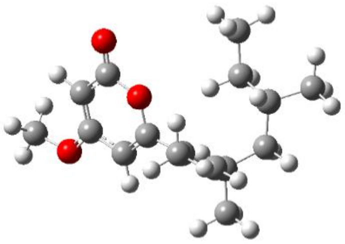

c

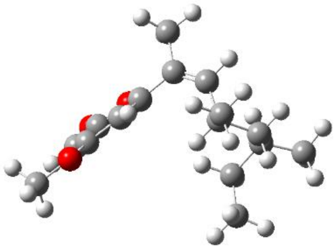

d

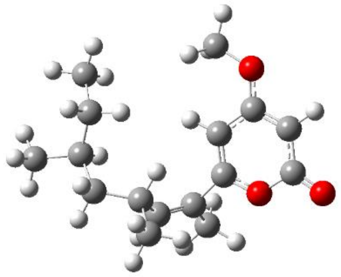

e

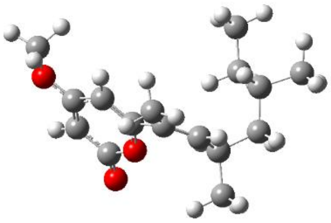

f

**Table S4-4.** B3LYP-D3/def2-TZVP/PCM energies (*E*), relative thermol energies (Δ*E*), relative free energies (Δ*G*), and equilibrium populations (P)[a] of low-energy conformers of 4'*R*,6'*S*-**6**.

| Conformer of (**6**) | *E* (Hartree) | Δ*E* (kcal/mol) | Δ*G* (kcal/mol) | P (%) |
|---|---|---|---|---|
| **a** | -849.8484835 | 1.4385282 | 2.845569597 | 3.68 |
| **b** | -849.8475661 | 2.013930594 | 3.415976187 | 1.39 |
| **c** | -849.8507755 | 0.0 | 0.0 | 41.76 |
| **d** | -849.8507754 | 0.000062751 | 0.0 | 41.76 |
| **e** | -849.8465137 | 2.674322118 | 3.785705079 | 0.46 |
| **f** | -849.8495137 | 0.791792118 | 3.543046962 | 10.96 |

[a] From Δ*G* values at 298.15 K.

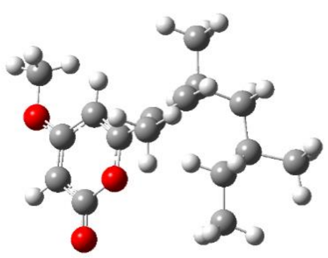

a

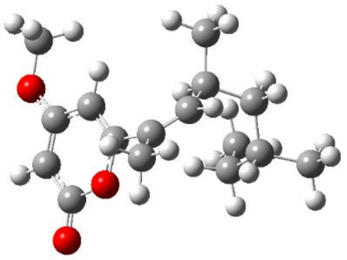

b

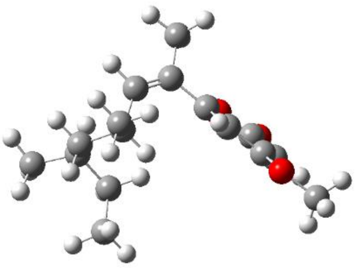

c

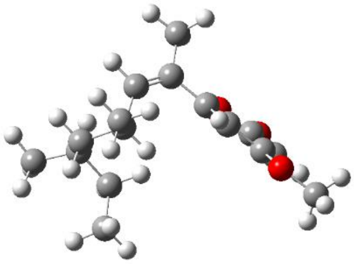

d

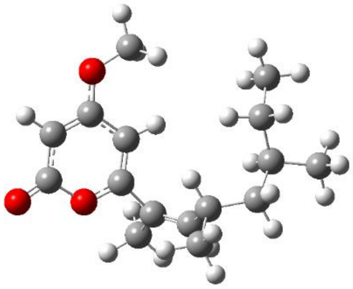

e

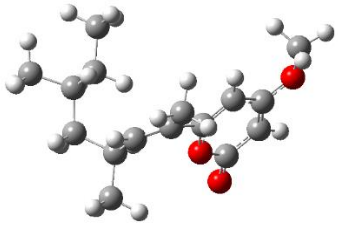

f

**Table S5-1.** B3LYP-D3/def2-TZVP/PCM energies (*E*), relative thermol energies (Δ*E*), relative free energies (Δ*G*), and equilibrium populations (P)[a] of low-energy conformers of 3*S*-**7**.

| Conformer of (**7**) | *E* (Hartree) | Δ*E* (kcal/mol) | Δ*G* (kcal/mol) | P (%) |
|---|---|---|---|---|
| **a** | -923.8246737 | 0.0 | 0.0 | 55.38 |
| **b** | -923.8233003 | 0.861822234 | 0.76689971 | 12.91 |
| **c** | -923.8235458 | 0.707768529 | 0.6064256664 | 15.75 |
| **d** | -923.822185 | 1.561684137 | 1.373682141 | 3.96 |
| **e** | -923.8184277 | 3.91942746 | 3.660454083 | 0.07 |
| **f** | -923.8231418 | 0.961282569 | 0.715424151 | 10.92 |

[a] From Δ*G* values at 298.15 K.

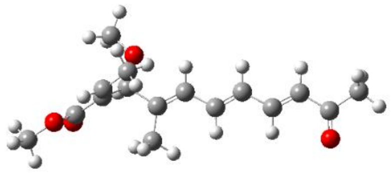
a

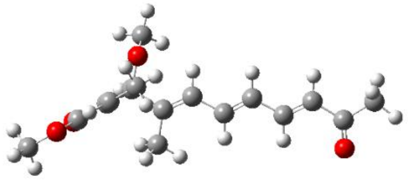
b

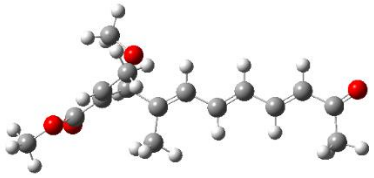
c

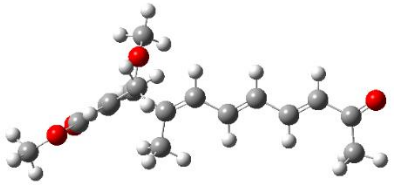
d

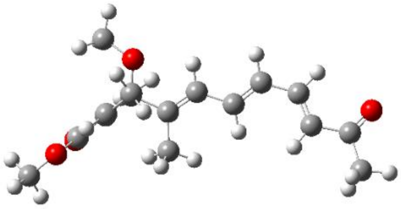
e

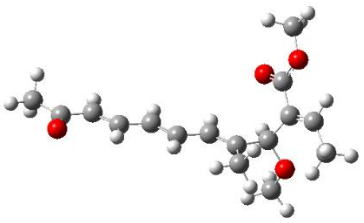
f

**Table S5-2.** B3LYP-D3/def2-TZVP/PCM energies (*E*), relative thermol energies (Δ*E*), relative free energies (Δ*G*), and equilibrium populations (P)[a] of low-energy conformers of 3*R*-**7**.

| Conformer of (**7**) | *E* (Hartree) | Δ*E* (kcal/mol) | Δ*G* (kcal/mol) | P (%) |
|---|---|---|---|---|
| **a** | -923.8246735 | 0.0 | 0.0 | 56.70 |
| **b** | -923.8233003 | 0.861696732 | 0.766879971 | 13.22 |
| **c** | -923.823545 | 0.708145035 | 0.606425664 | 17.14 |
| **d** | -923.8184268 | 3.919866717 | 3.660454083 | 0.08 |
| **e** | -923.8232741 | 0.878137494 | 0.479354889 | 12.86 |
| **f**[b] | - | - | - | - |

[a] From Δ*G* values at 298.15 K.

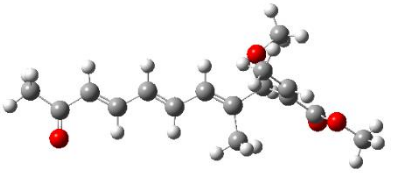

a

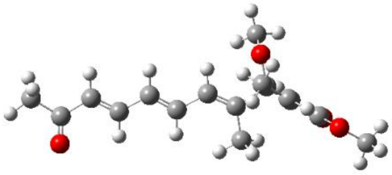

b

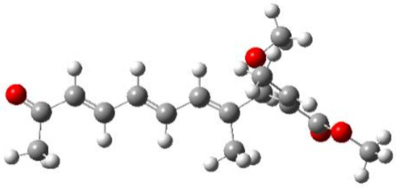

c

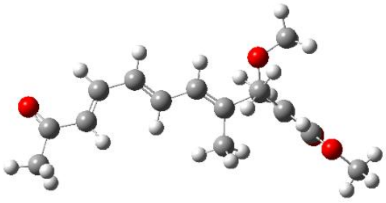

d

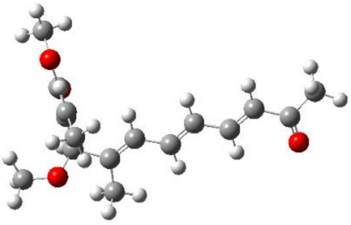

e

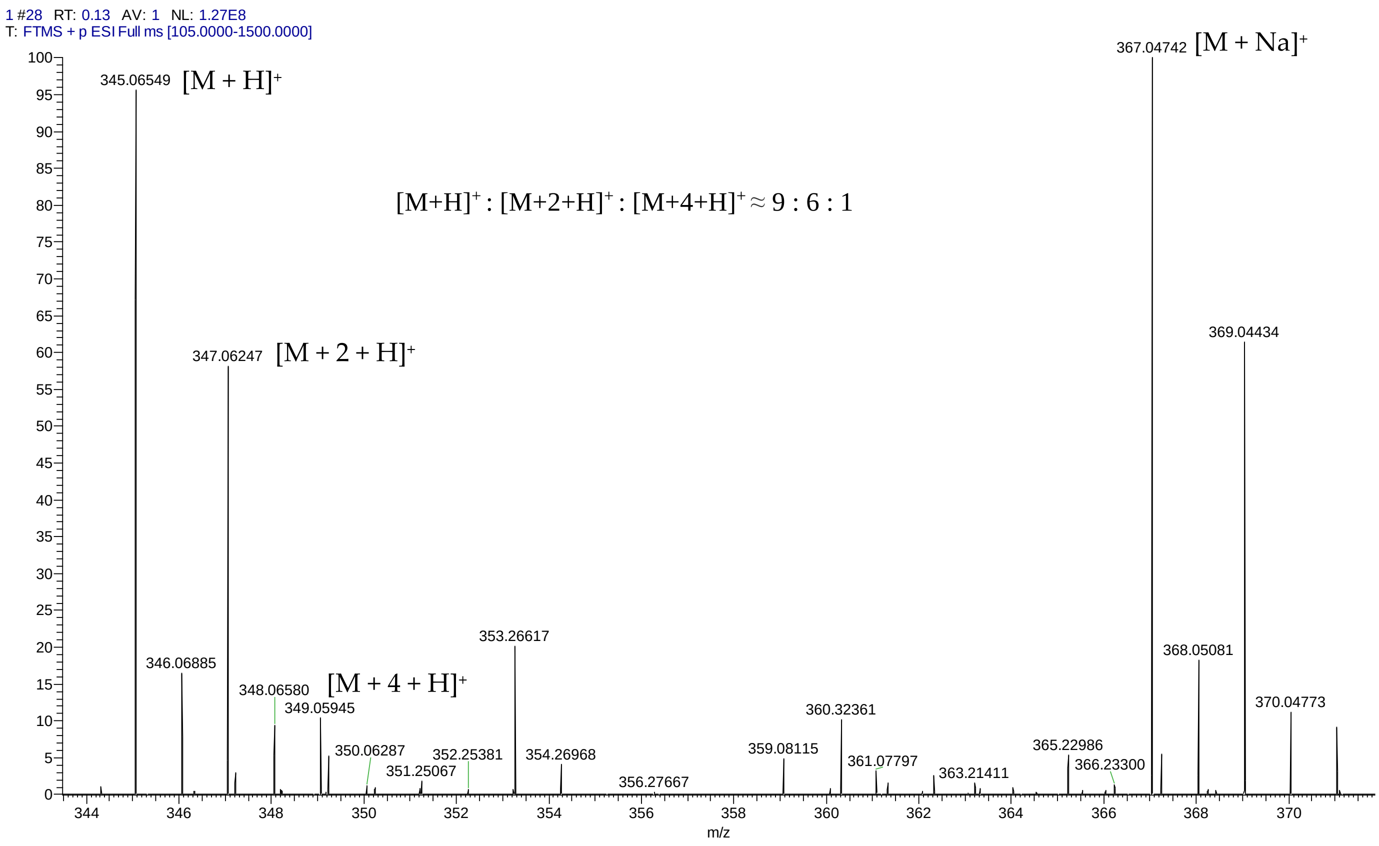


**Figure S1-1**. HRESIMS spectrum of compound **1**

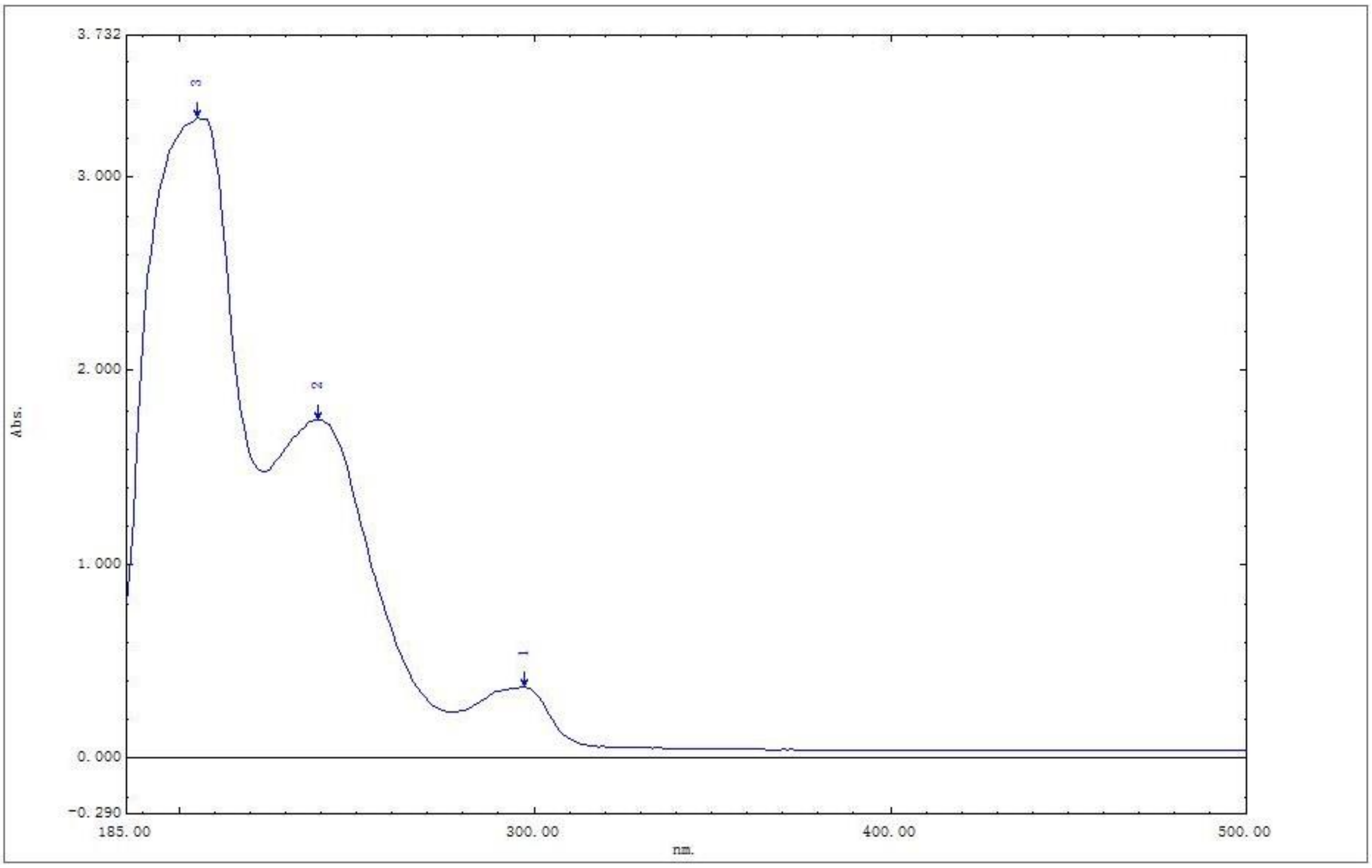


**Figure S1-2.** UV spectrum of compound **1**

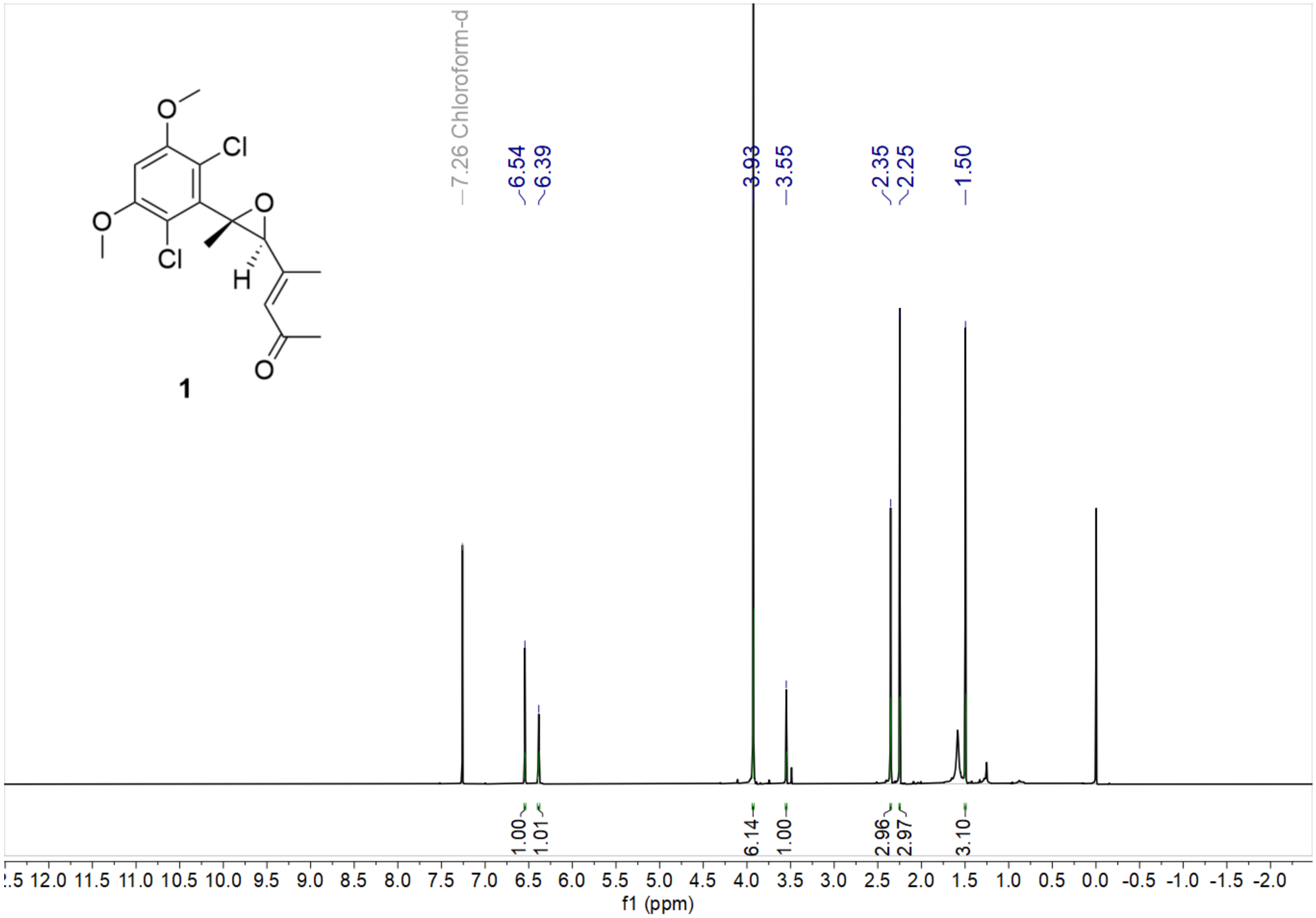


**Figure S1-3**. $^{1}$H NMR spectrum of **1** in $CDCl_3$ (400 MHz)

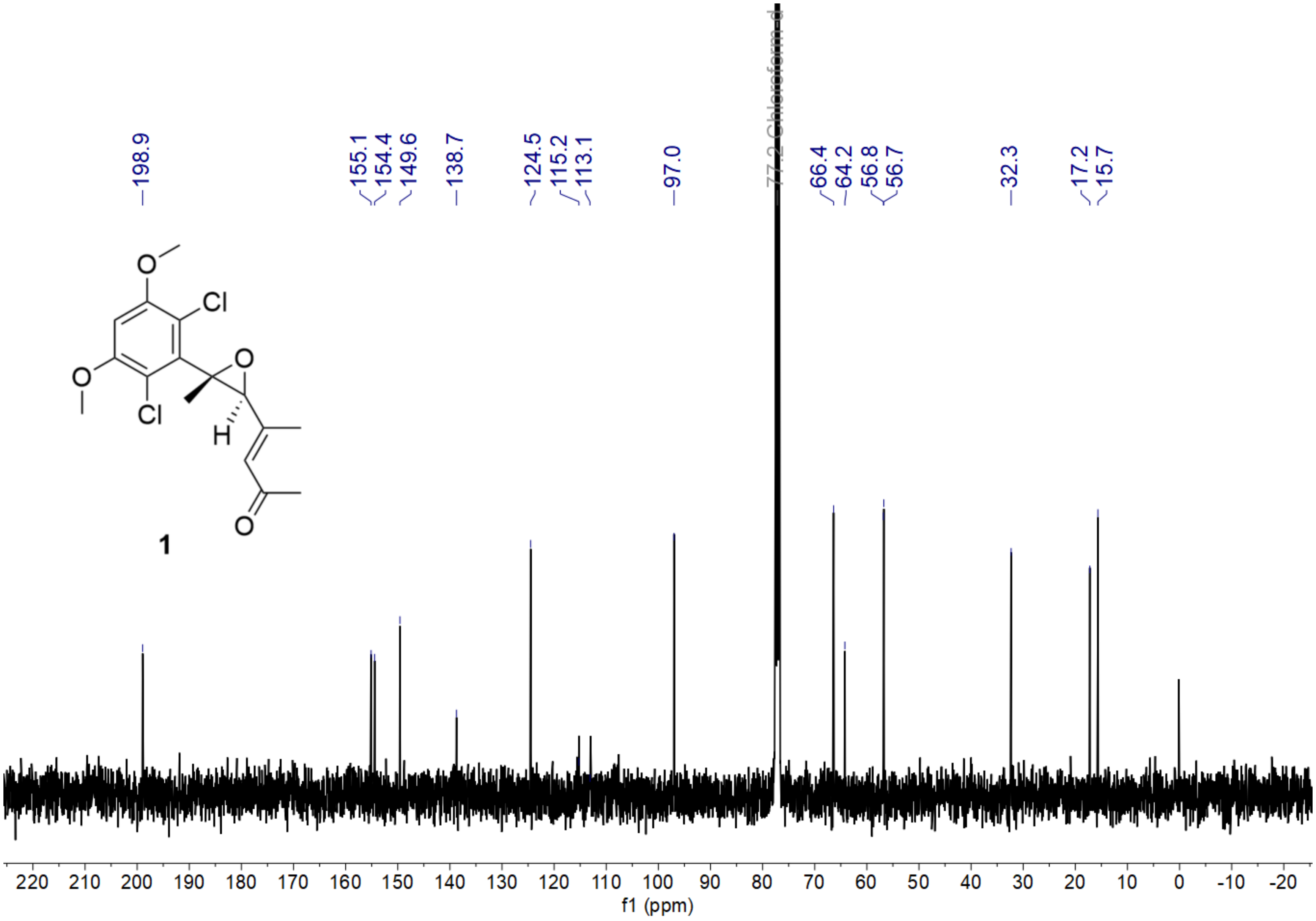


**Figure S1-4.** $^{13}C$ NMR spectrum of **1** in $CDCl_3$ (100 MHz)

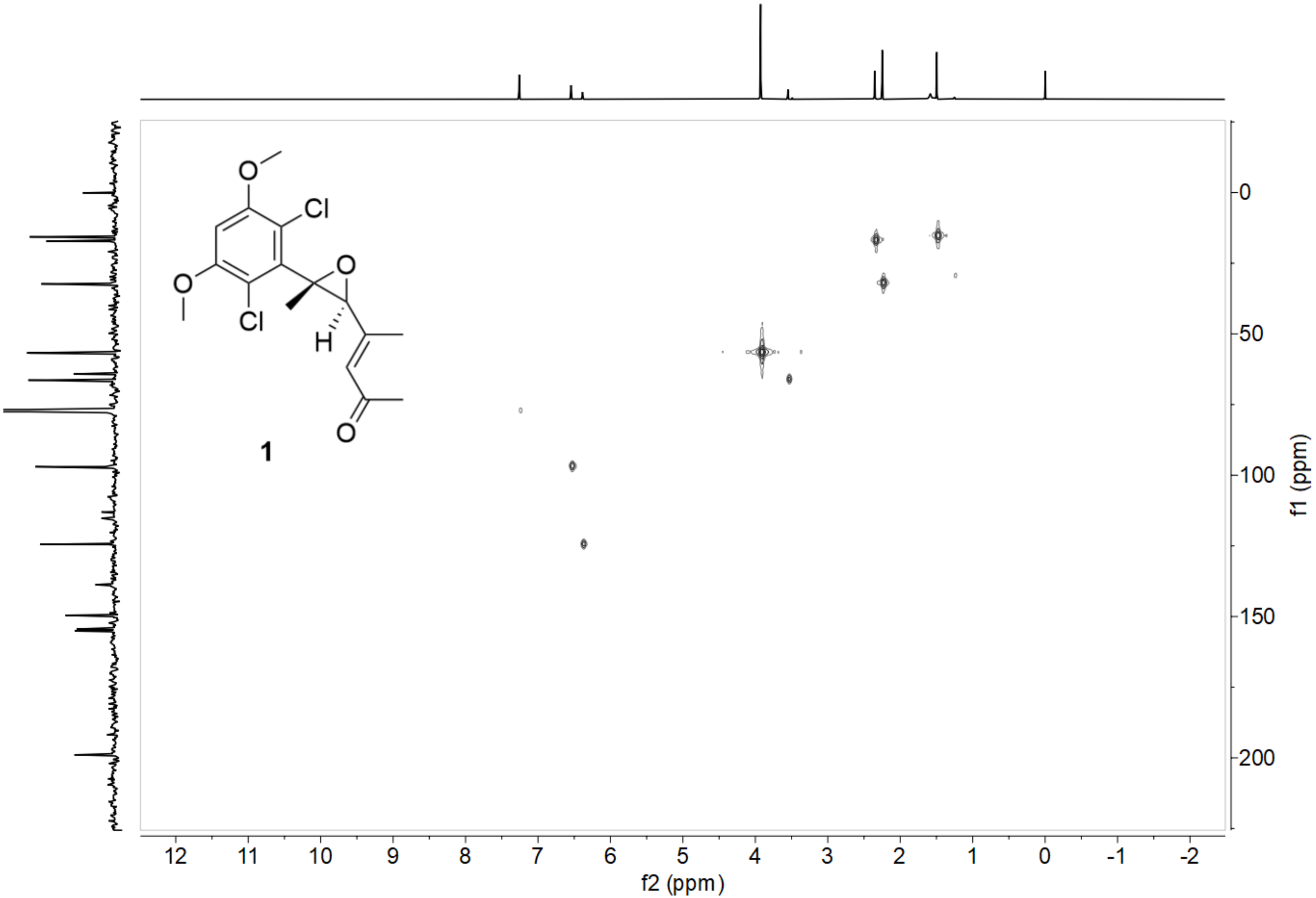


**Figure S1-5.** HMQC spectrum of **1** in $CDCl_3$

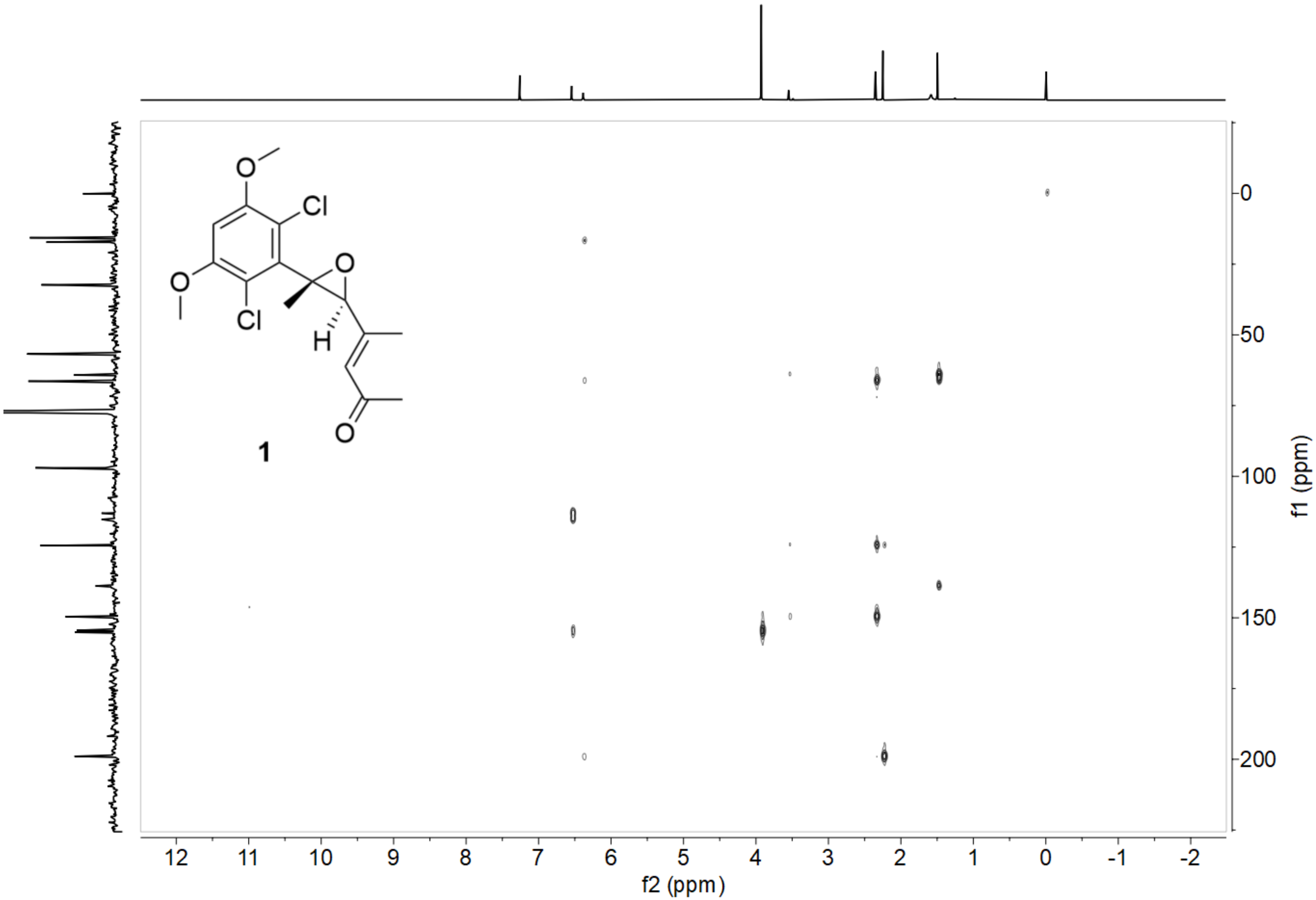


**Figure S1-6.** HMBC spectrum of **1** in $CDCl_3$

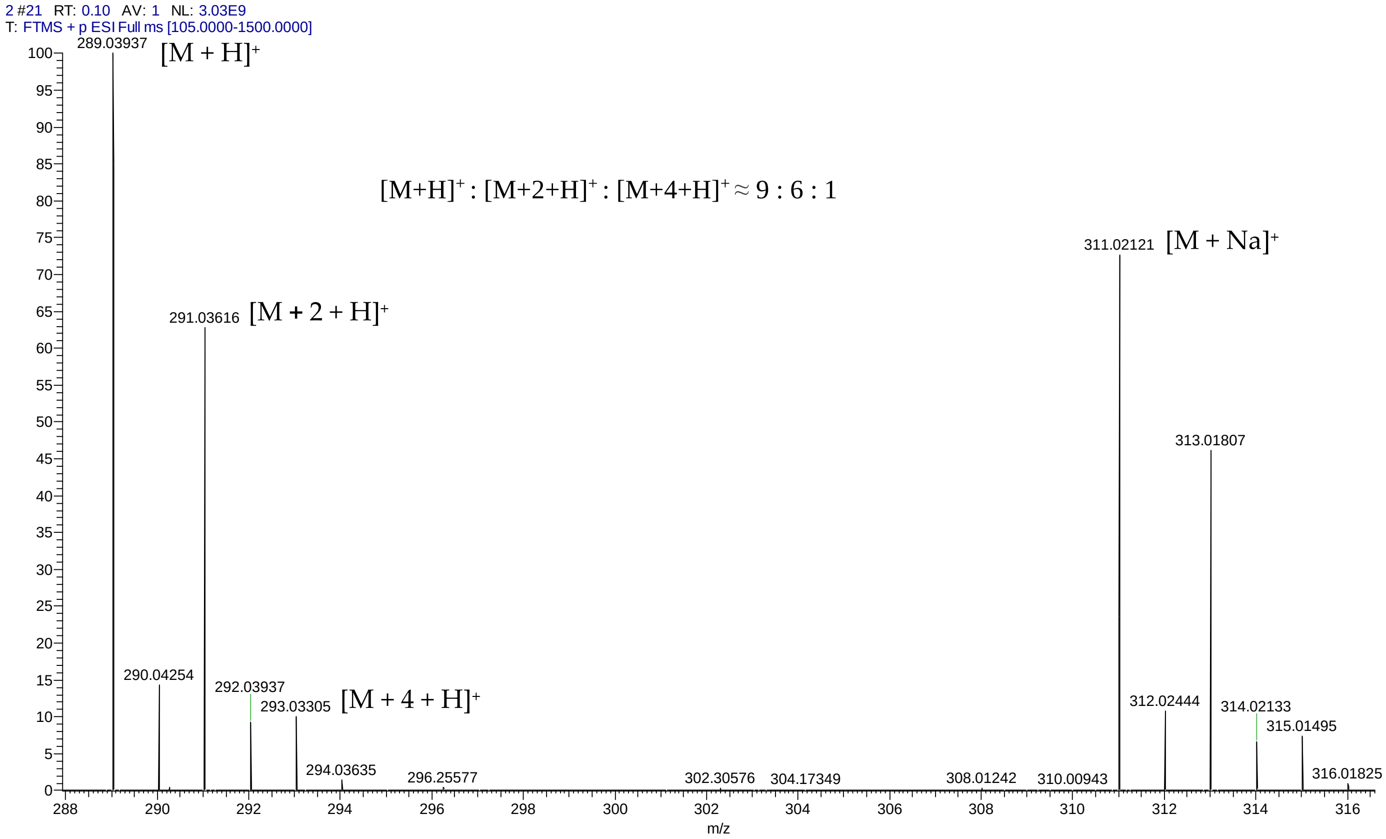


**Figure S2-1.** HRESIMS spectrum of compound **2**

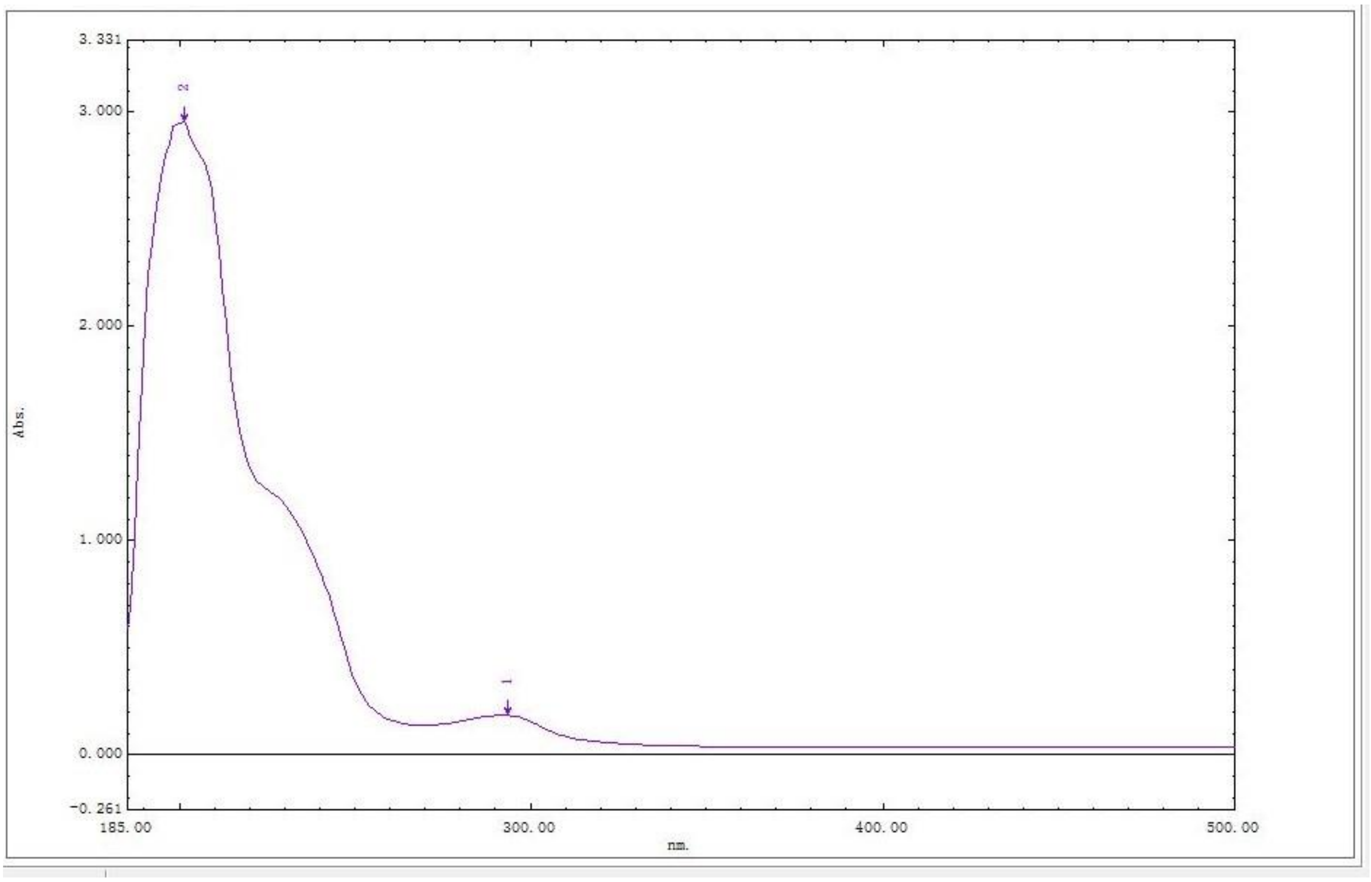


**Figure S2-2.** UV spectrum of compound **2**

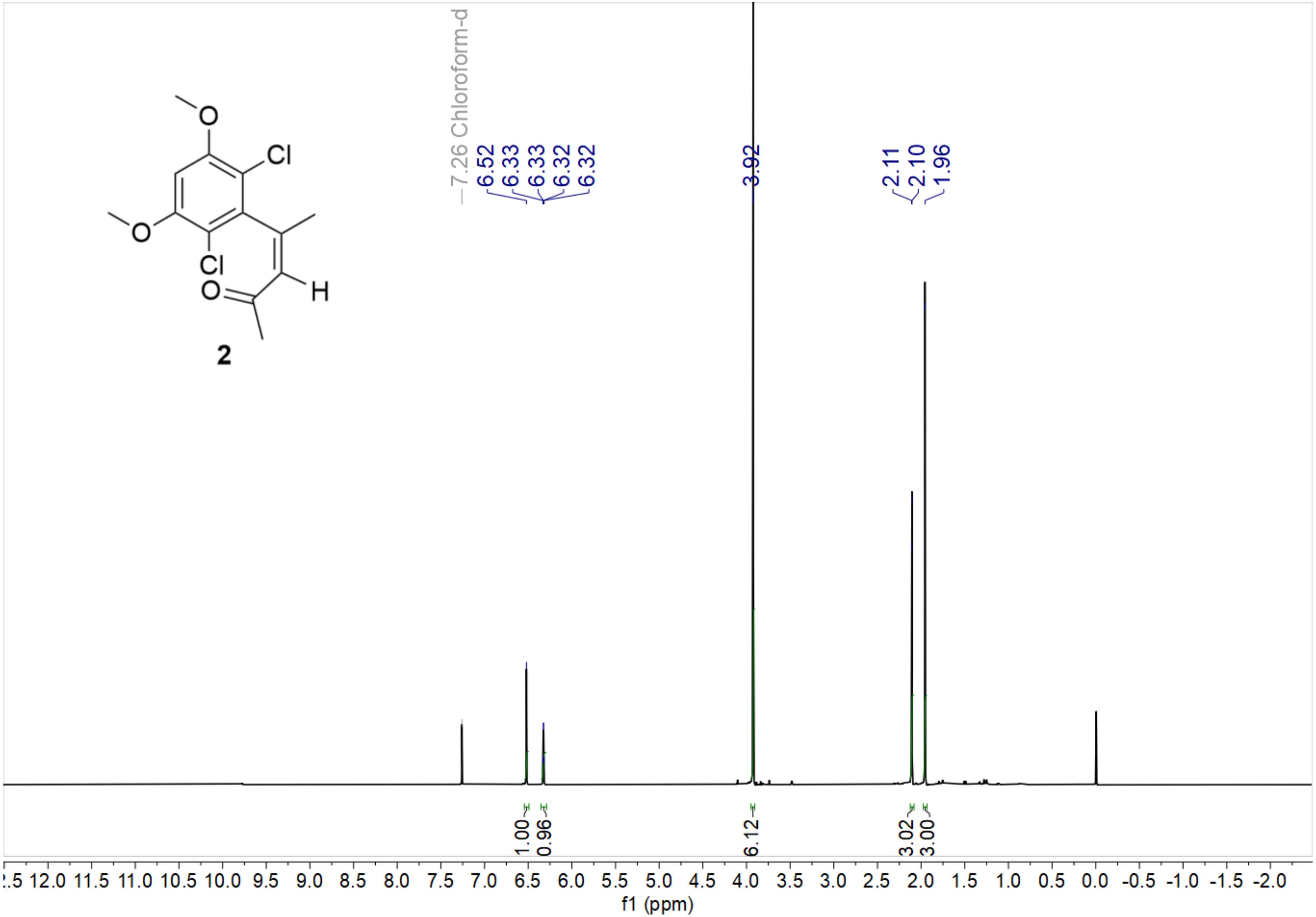


**Figure S2-3.** $^{1}$H NMR spectrum of **2** in $CDCl_3$ (400 MHz)

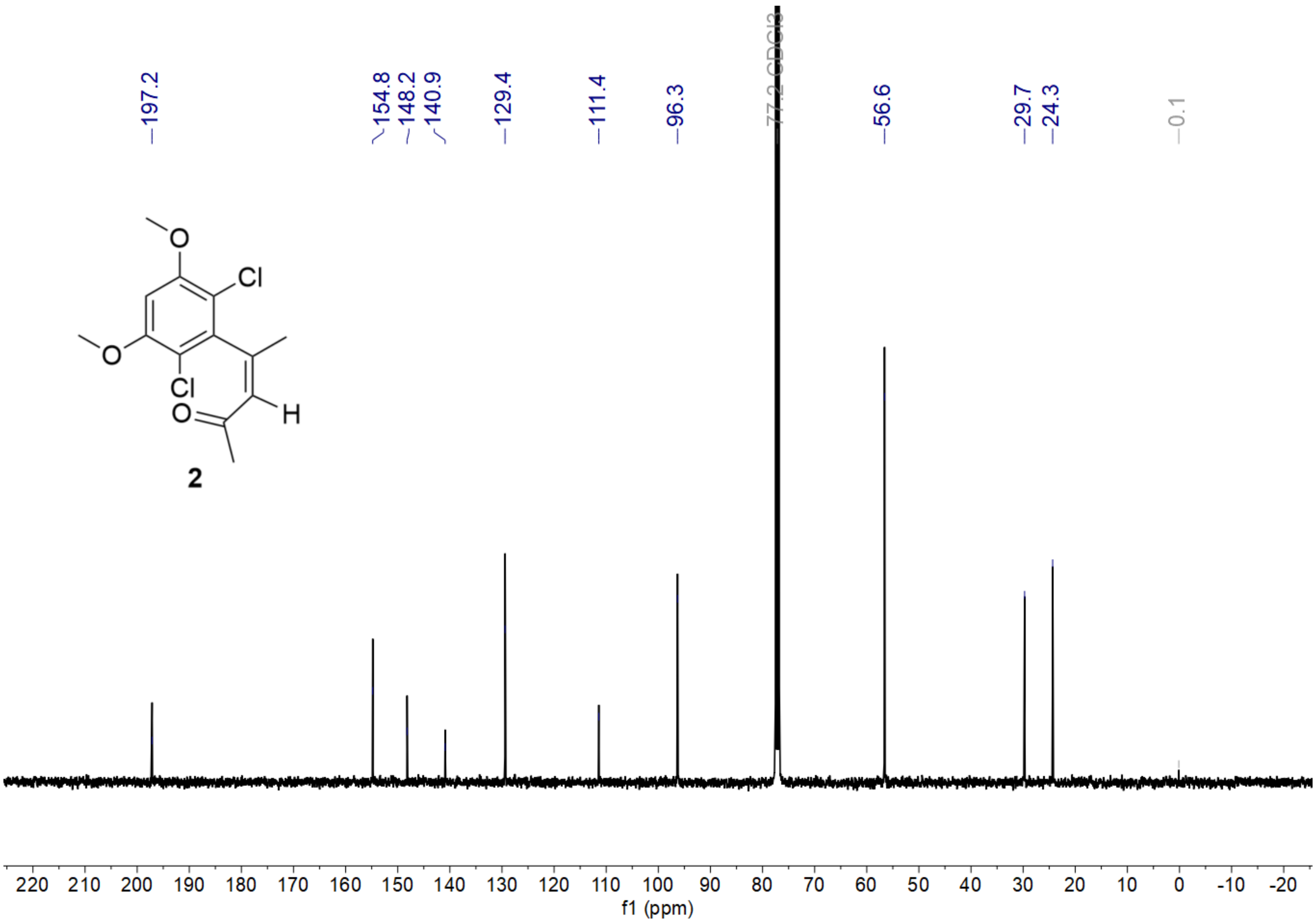


**Figure S2-4.** $^{13}C$ NMR spectrum of **2** in $CDCl_3$ (100 MHz)

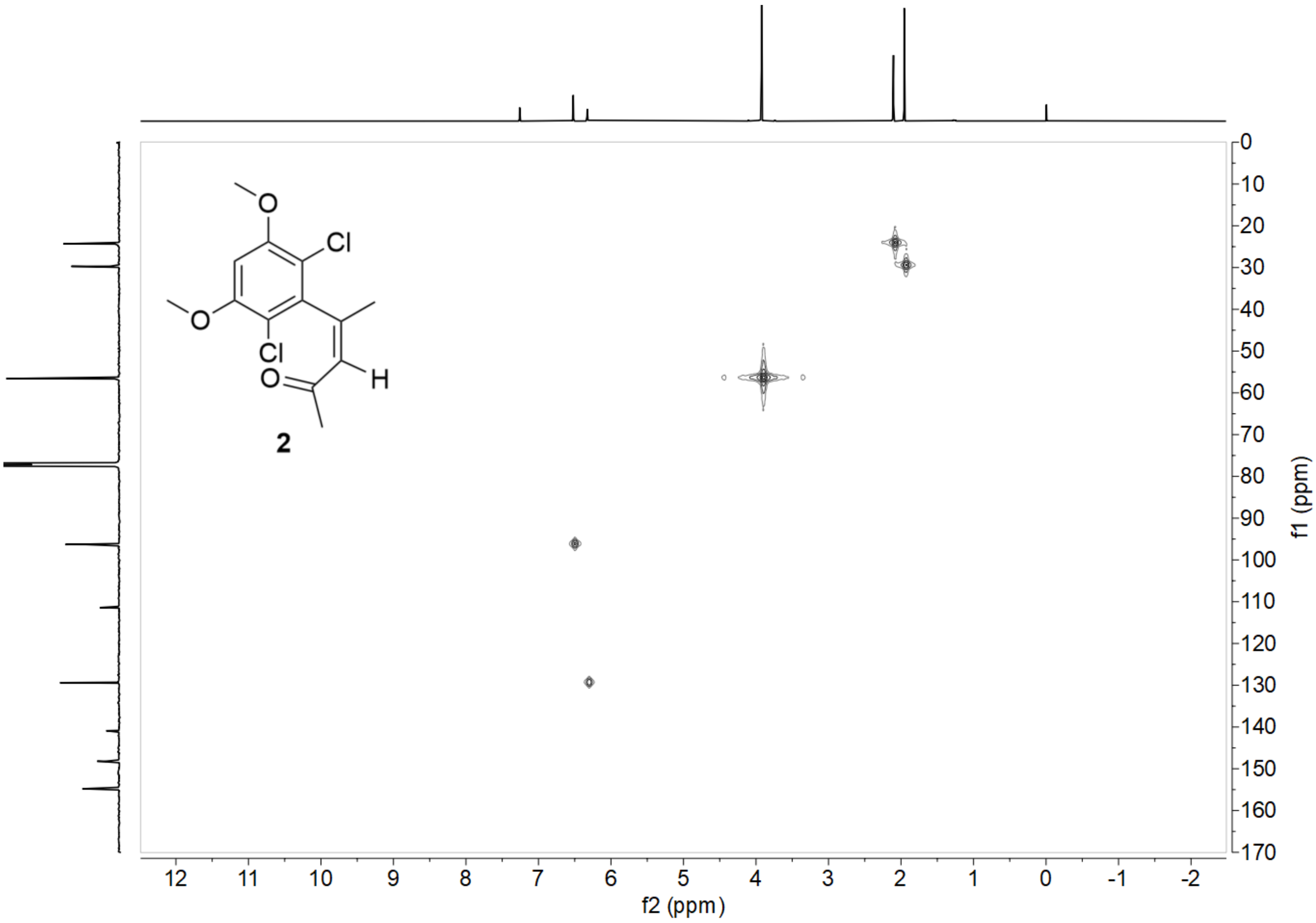


**Figure S2-5.** HMQC spectrum of **2** in $CDCl_3$

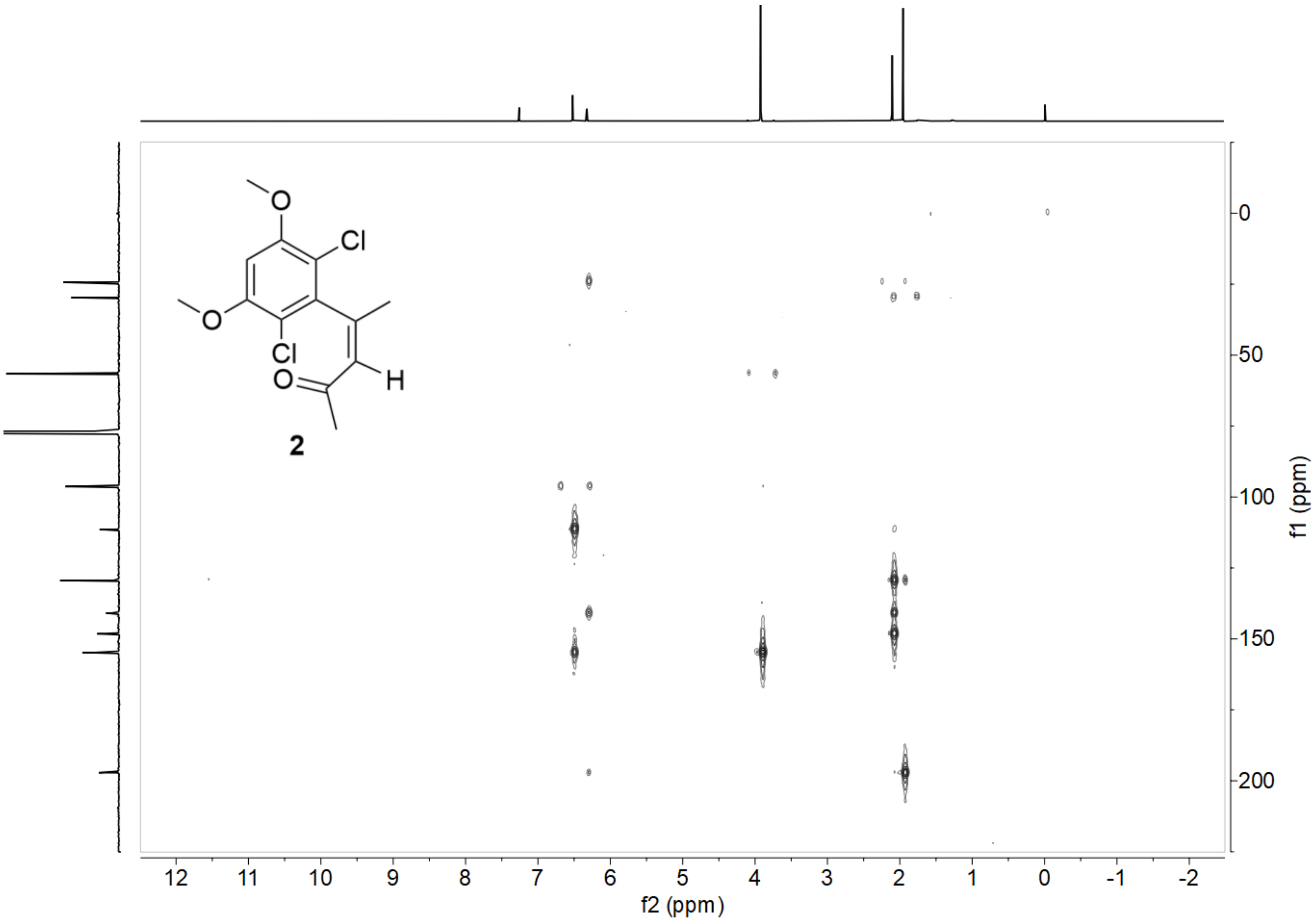


**Figure S2-6.** HMBC spectrum of **2** in $CDCl_3$

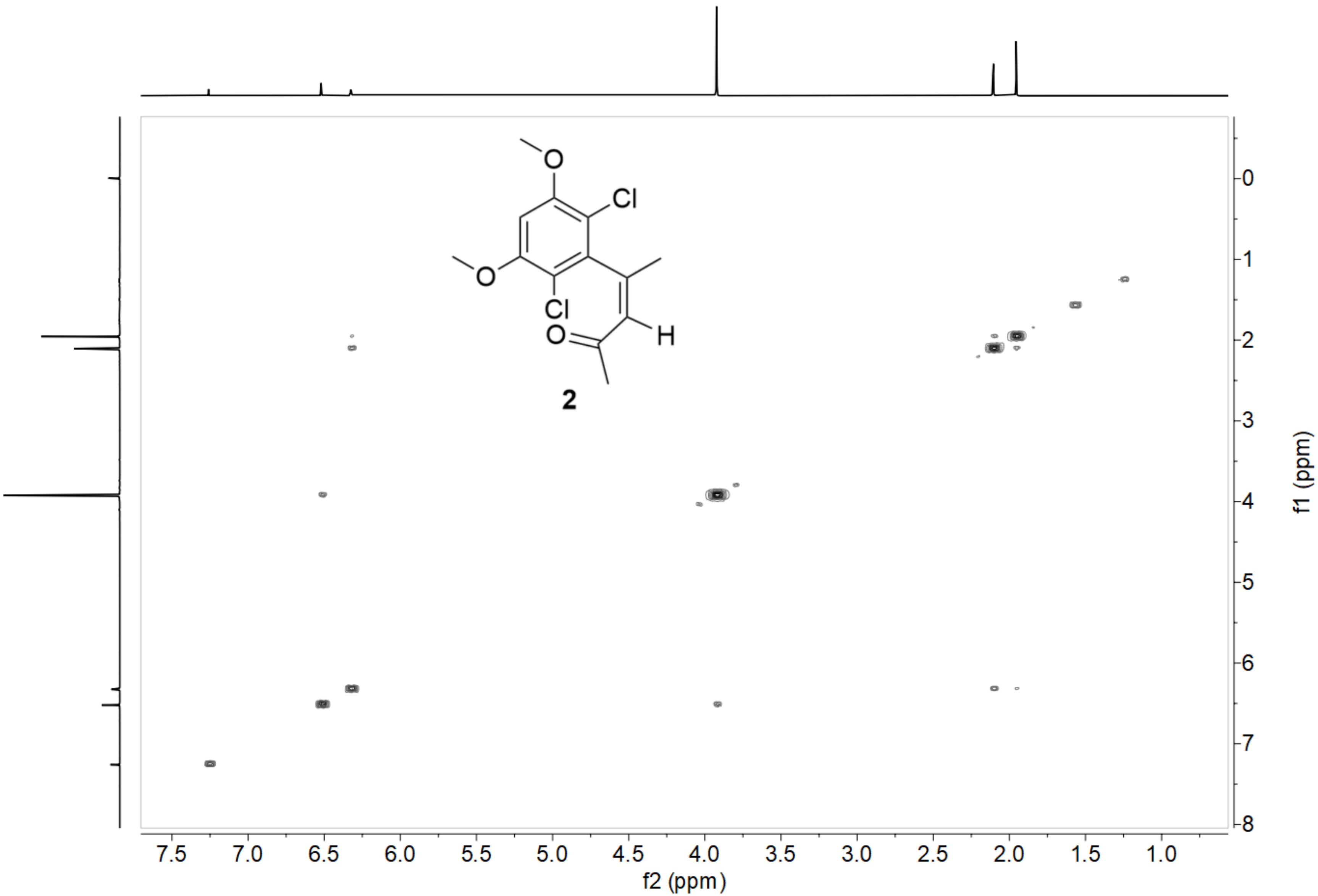


**Figure S2-7.** $^1H$-$^1H$ COSY spectrum of **2** in $CDCl_3$

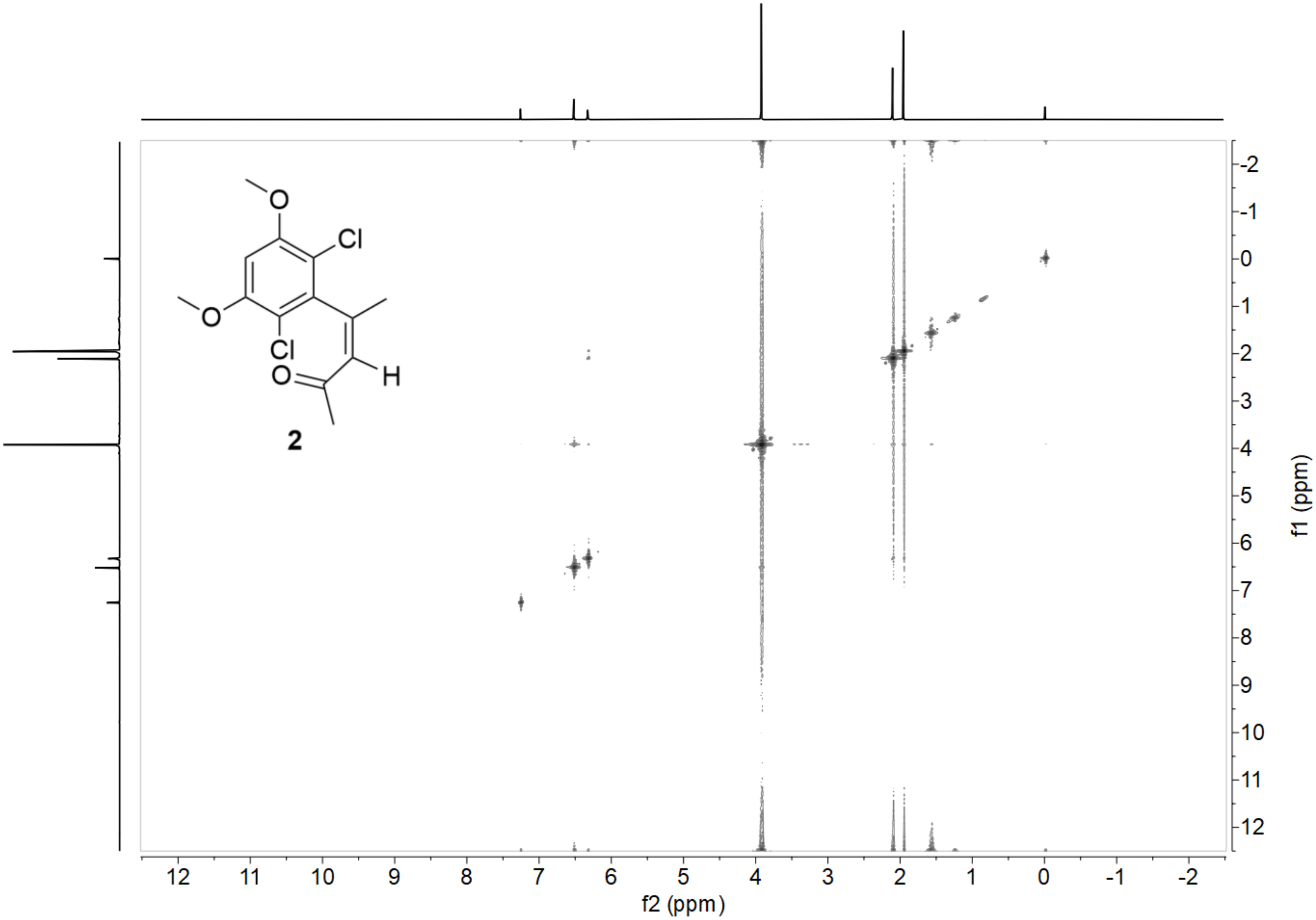


**Figure S2-8.** NOESY spectrum of **2** in $CDCl_3$

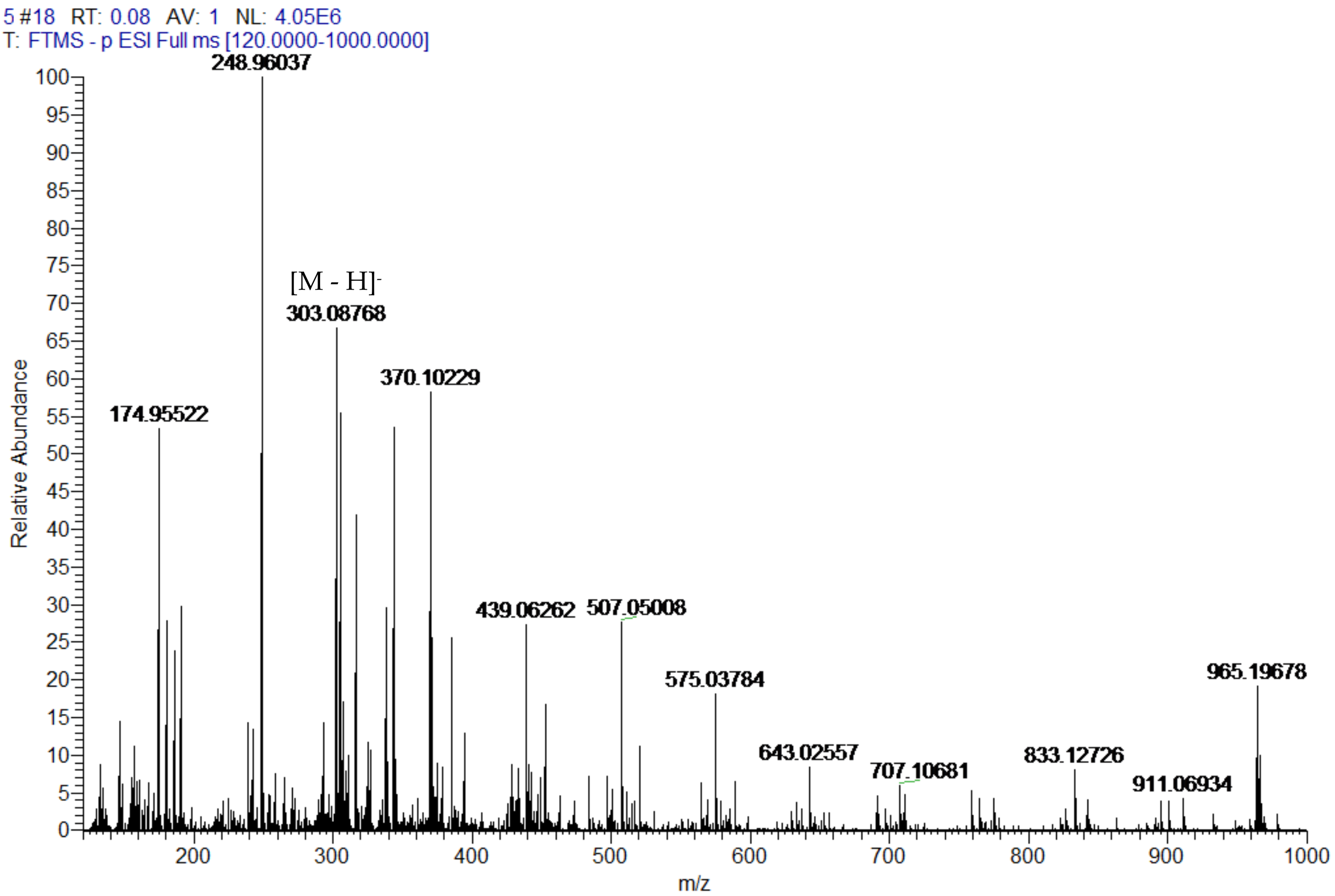


**Figure S3-1.** HRESIMS spectrum of compound **3**

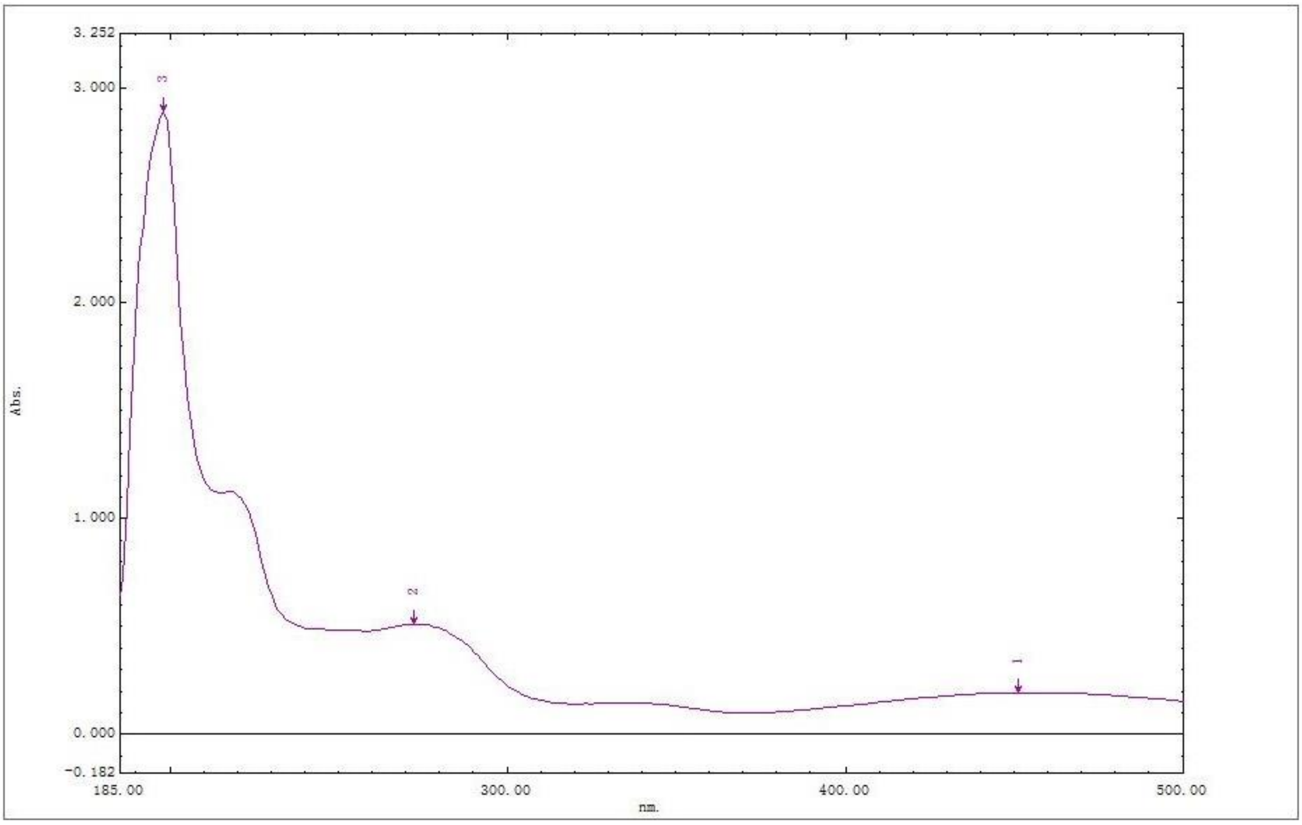


**Figure S3-2.** UV spectrum of compound **3**

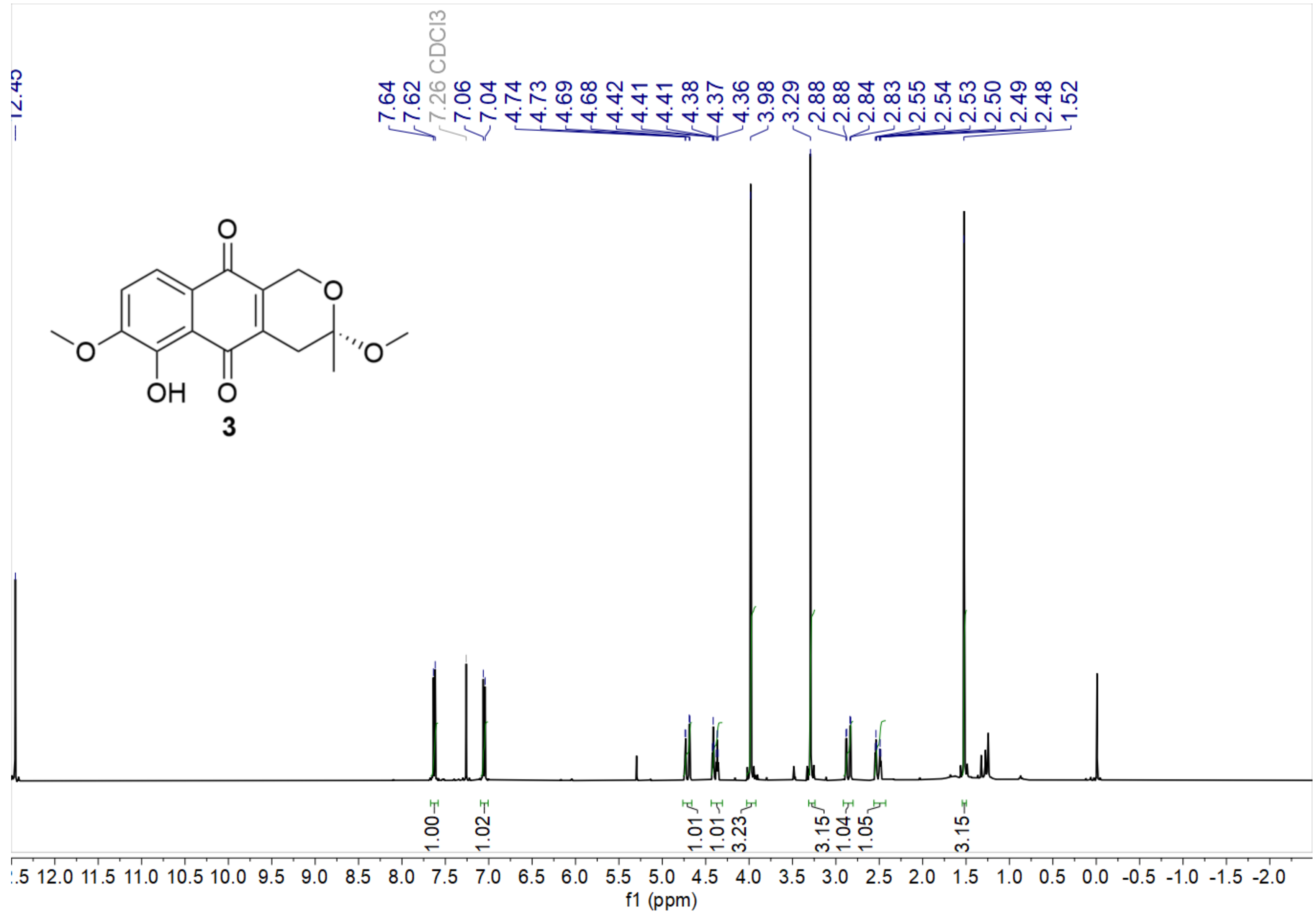


**Figure S3-3.** $^{1}$H NMR spectrum of **3** in $CDCl_3$ (400 MHz)

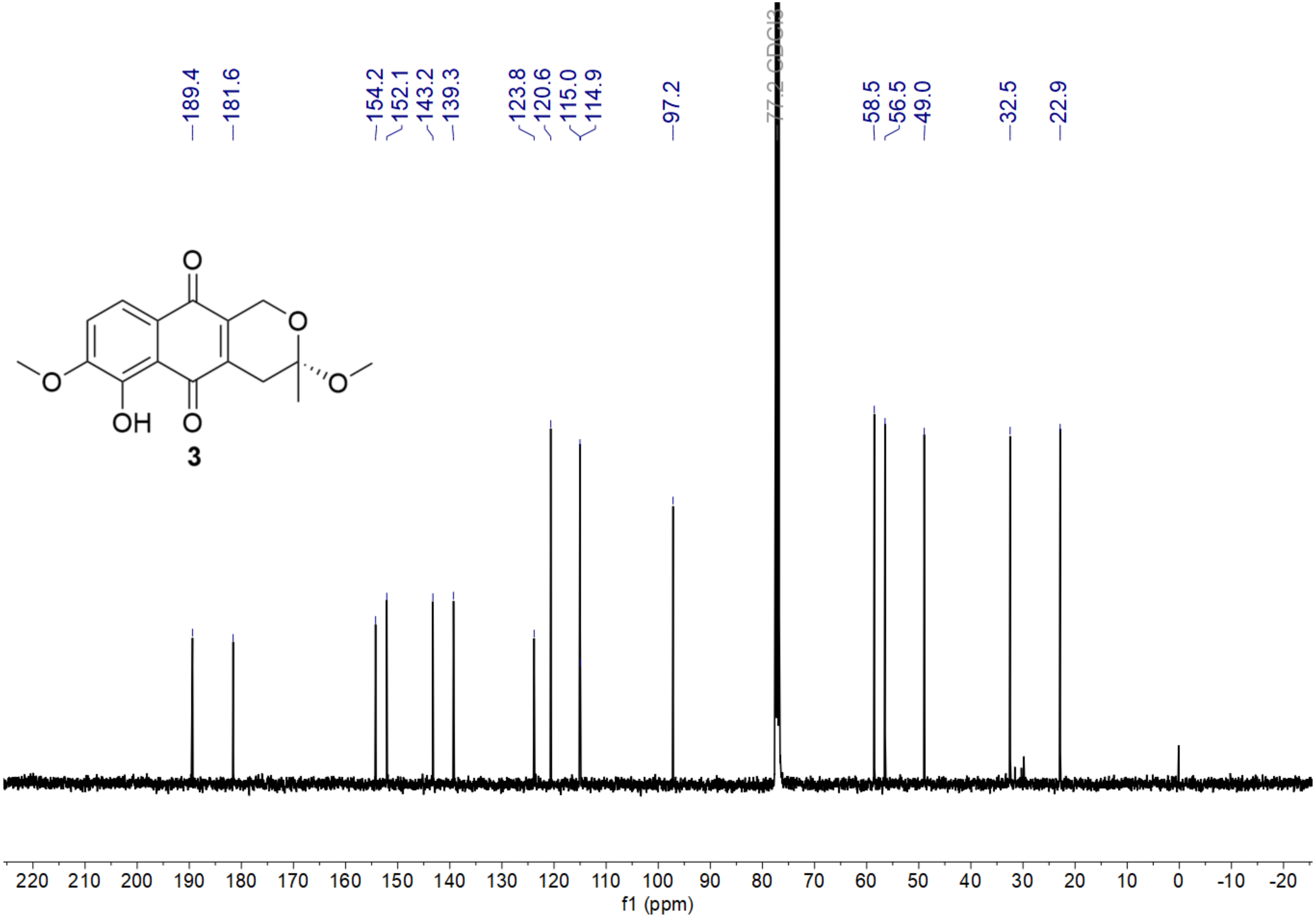


**Figure S3-4.** $^{13}C$ NMR spectrum of **3** in $CDCl_3$ (100 MHz)

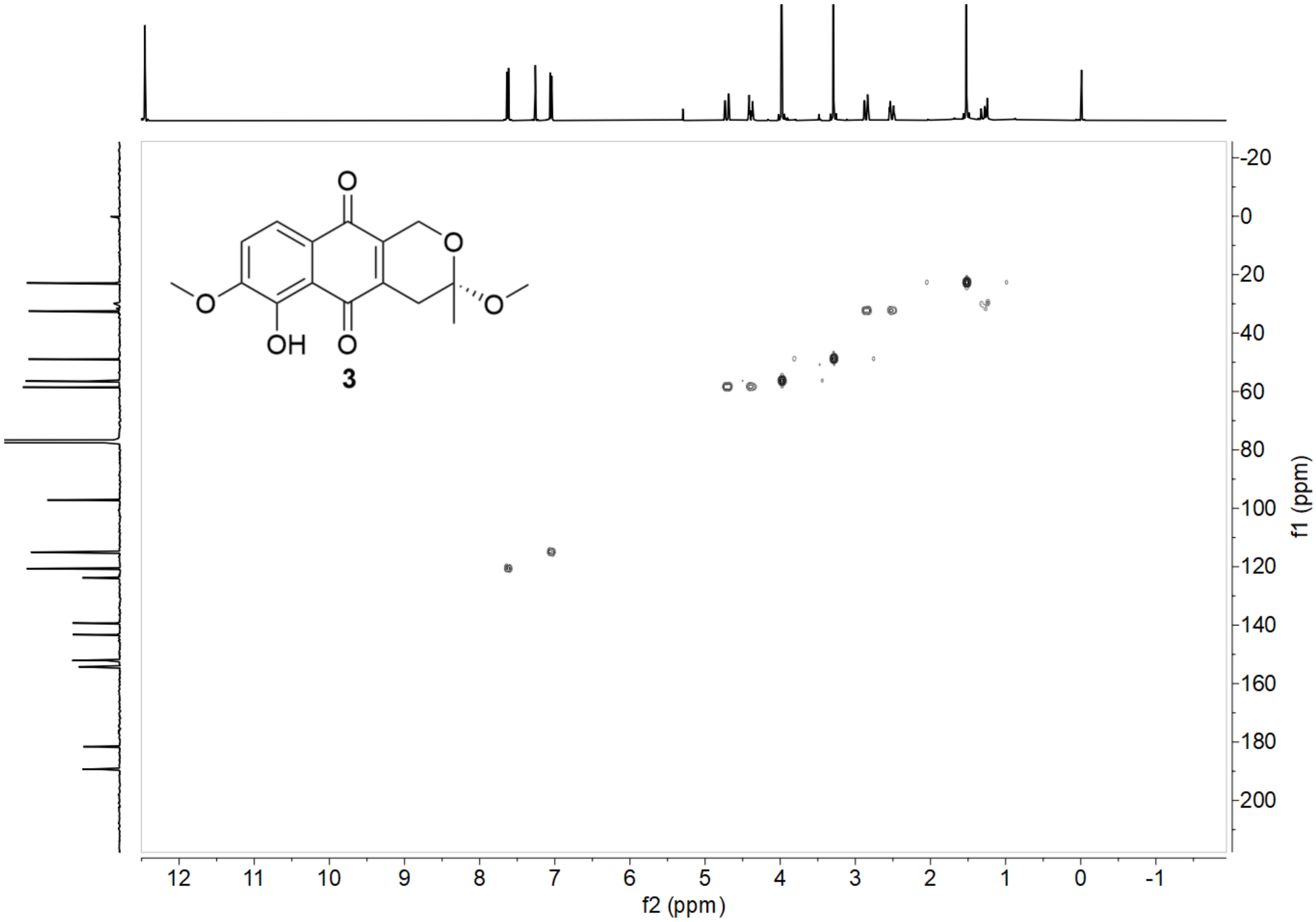


**Figure S3-5.** HMQC spectrum of **3** in $CDCl_3$

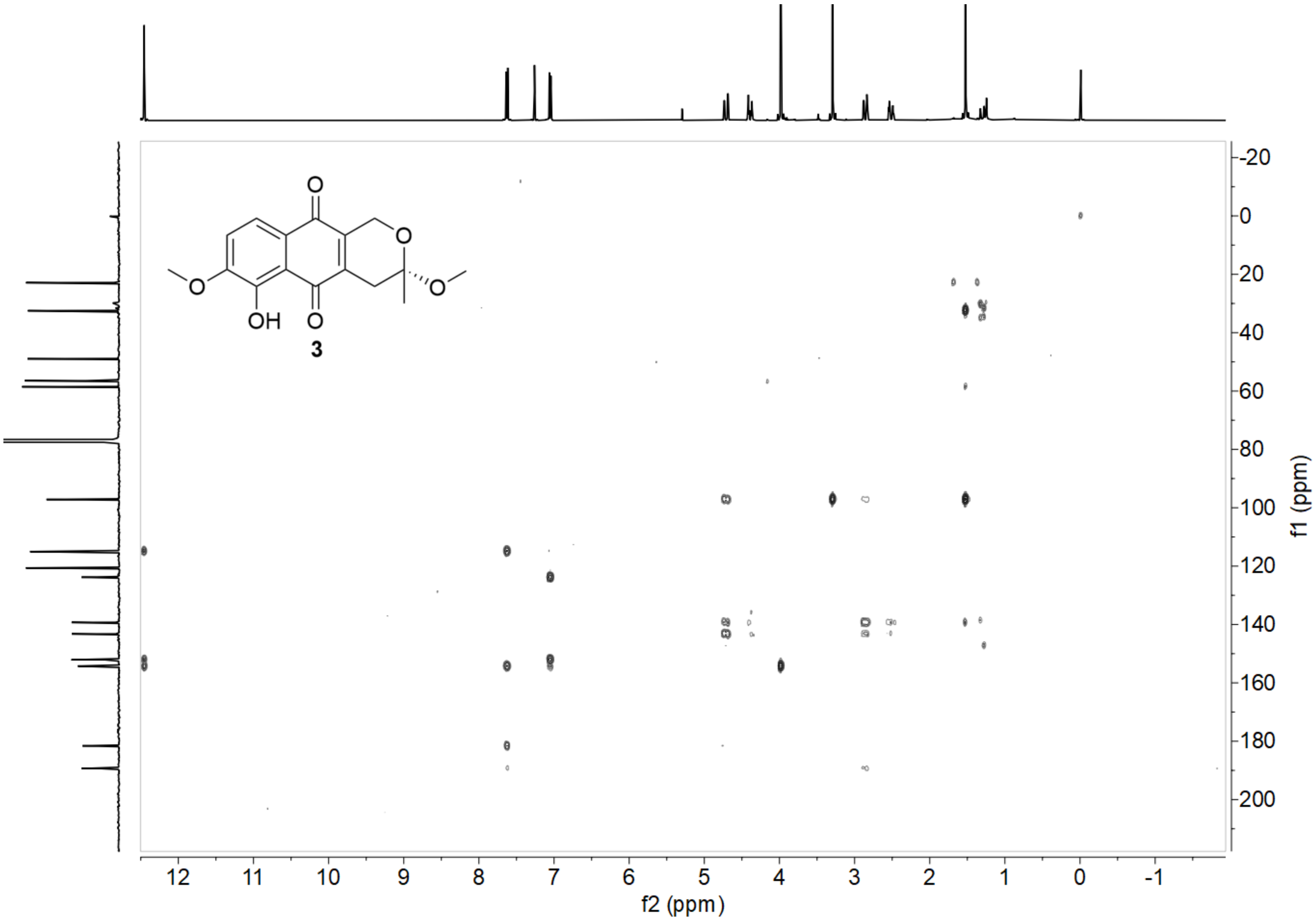


**Figure S3-6.** HMBC spectrum of **3** in $CDCl_3$

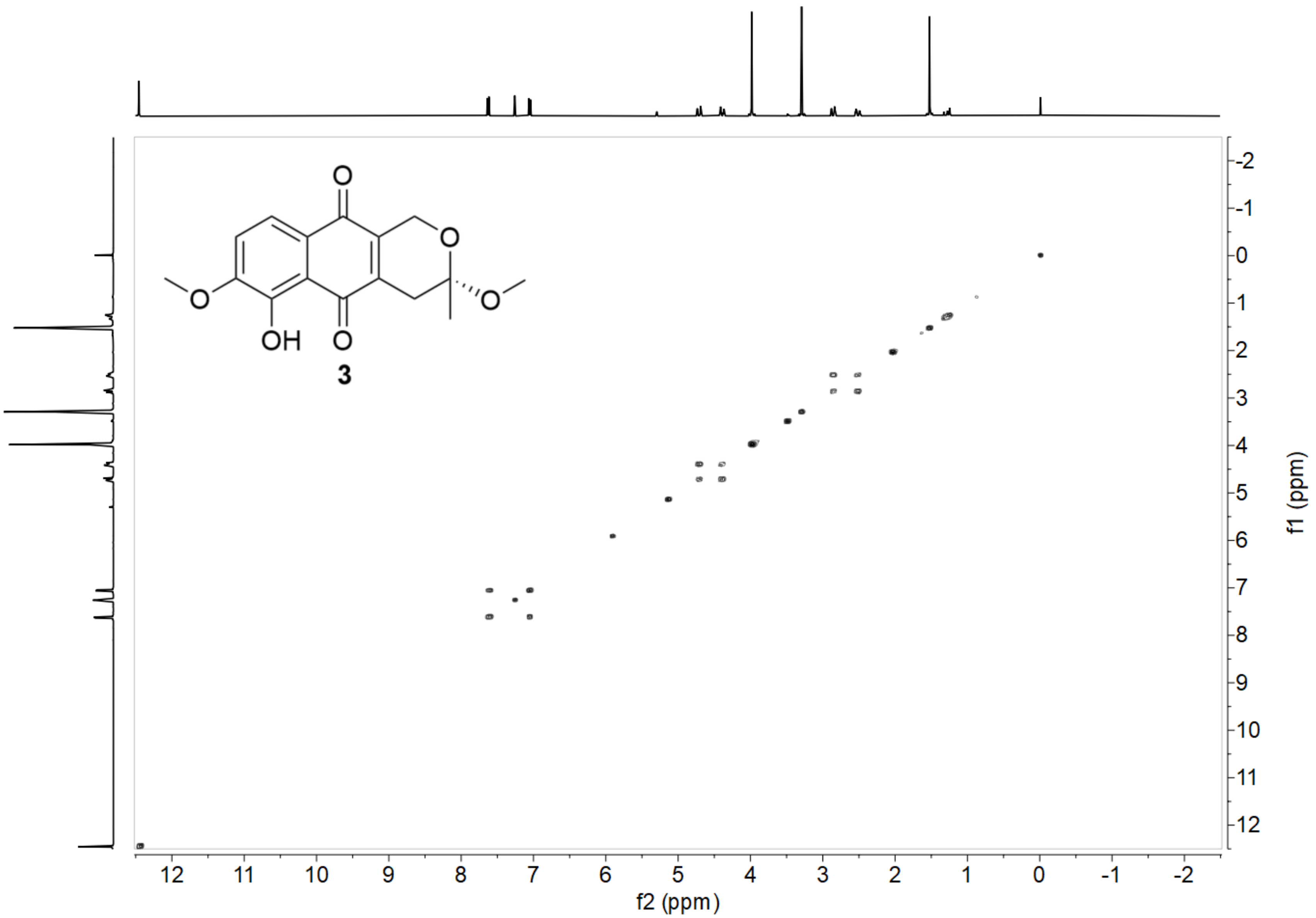


**Figure S3-7.** $^{1}$H-$^{1}$H COSY spectrum of **3** in $CDCl_3$

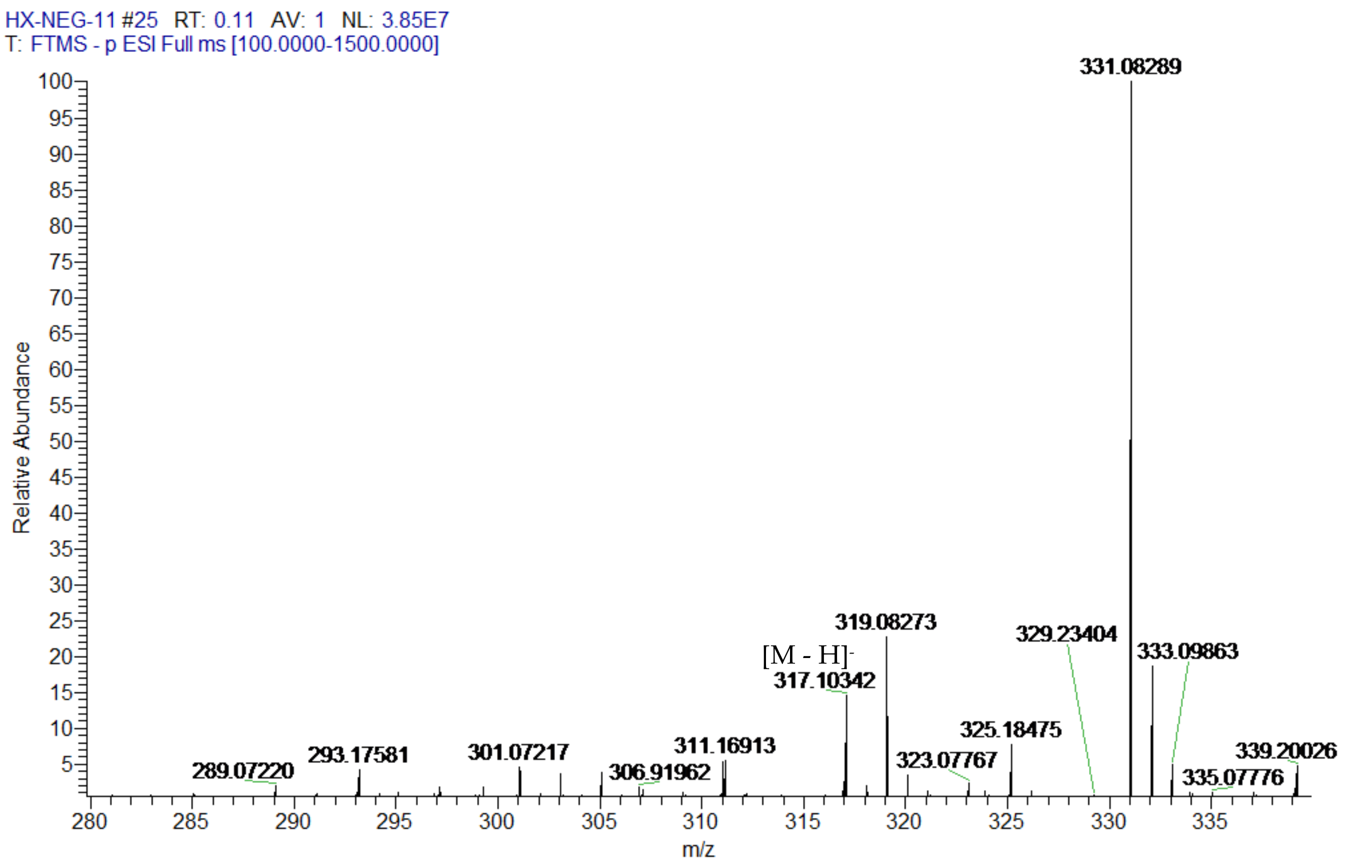


**Figure S4-1.** HRESIMS spectrum of compound **4**

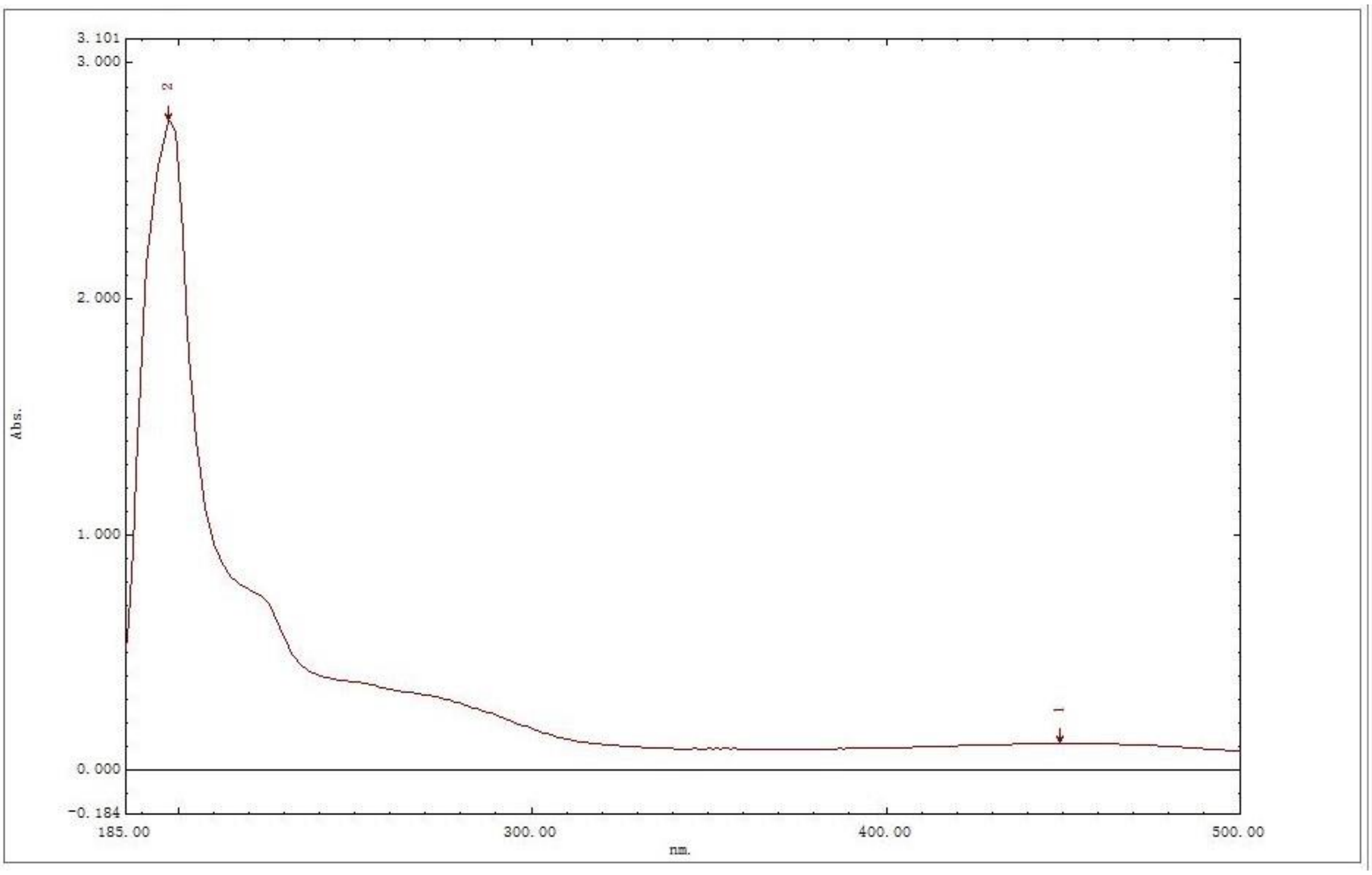


**Figure S4-2.** UV spectrum of compound **4**

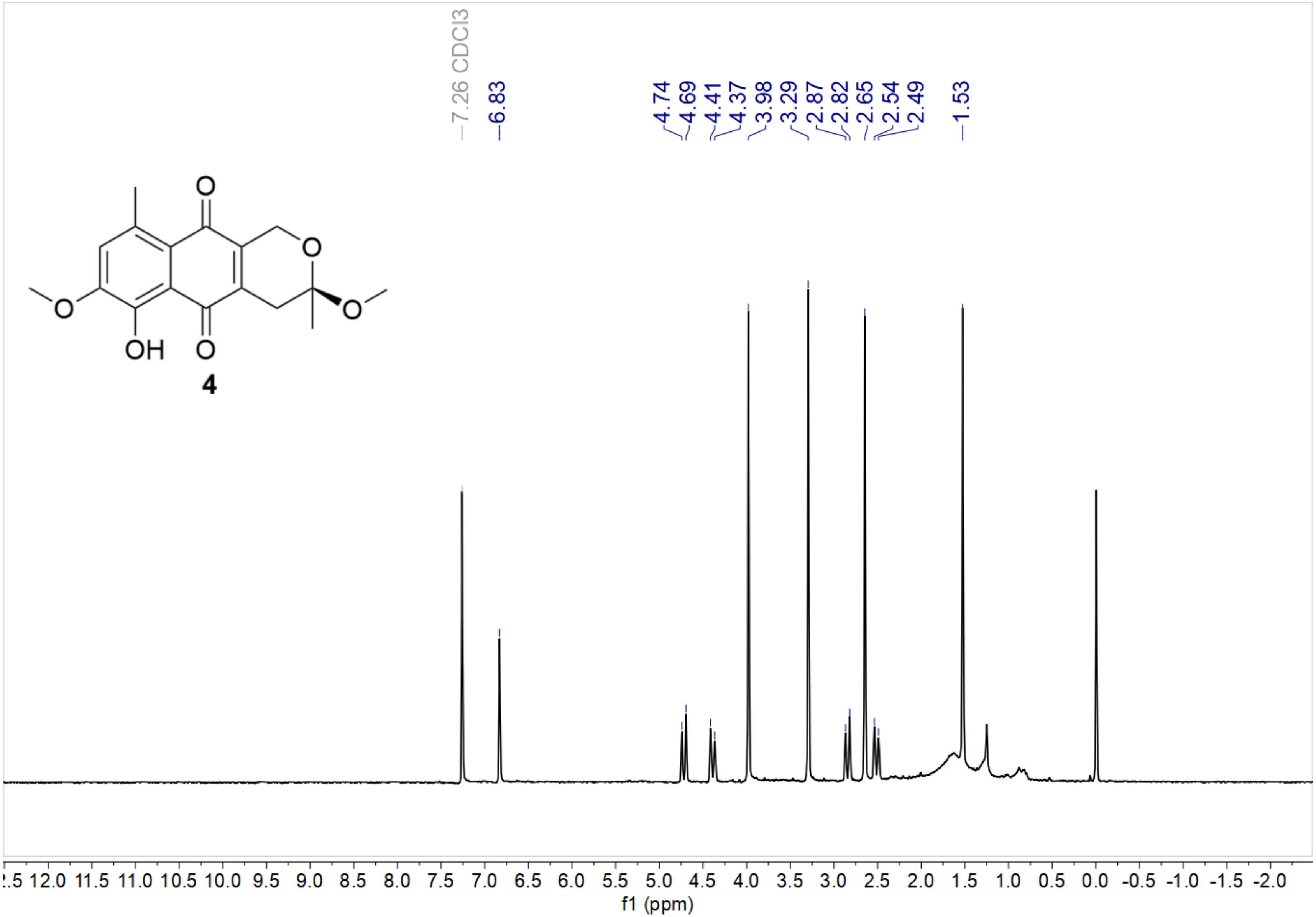


**Figure S4-3.** $^1$H NMR spectrum of **4** in $CDCl_3$ (400 MHz)

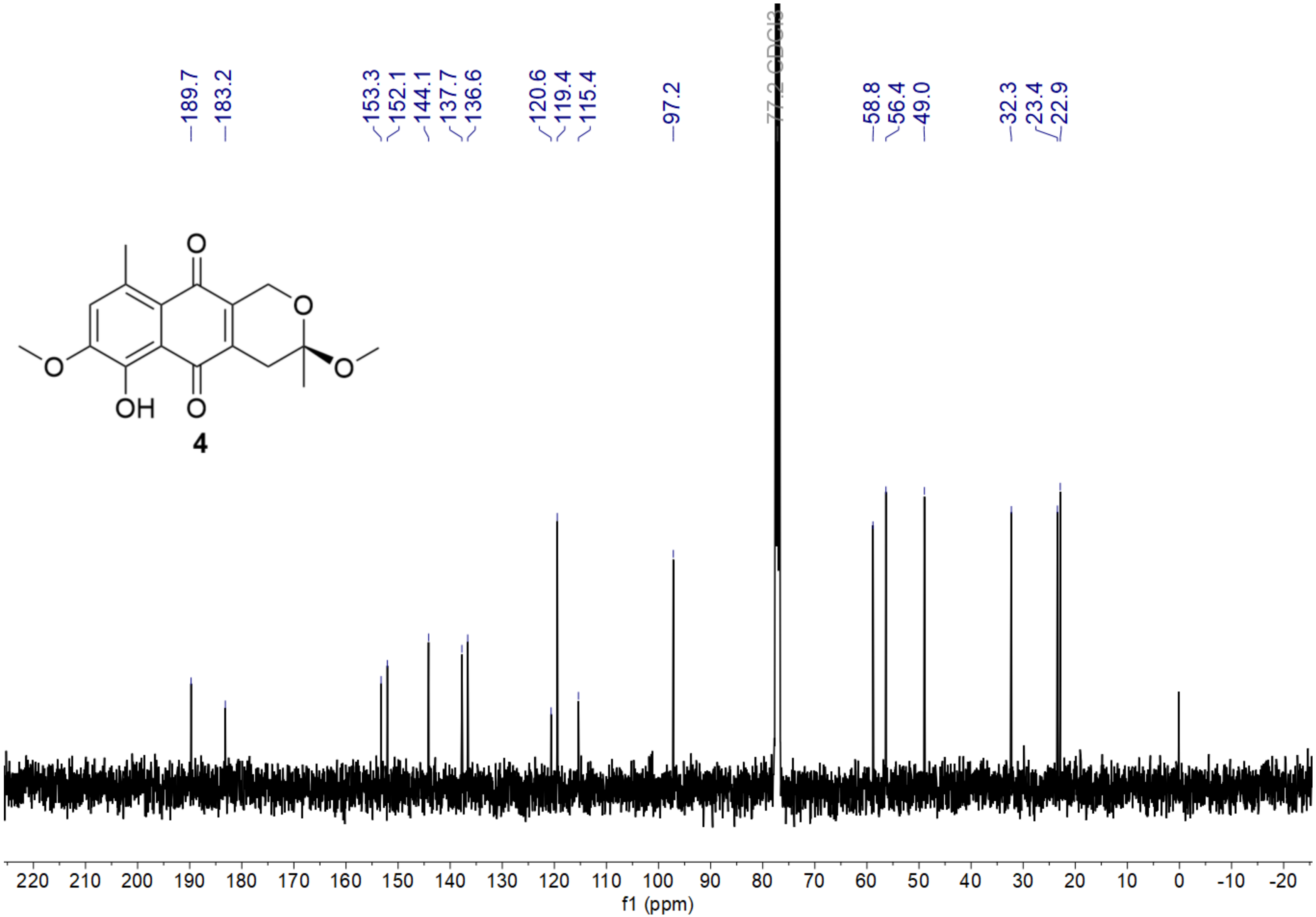


**Figure S4-4** $^{13}$C NMR spectrum of **4** in $CDCl_3$ (100 MHz)

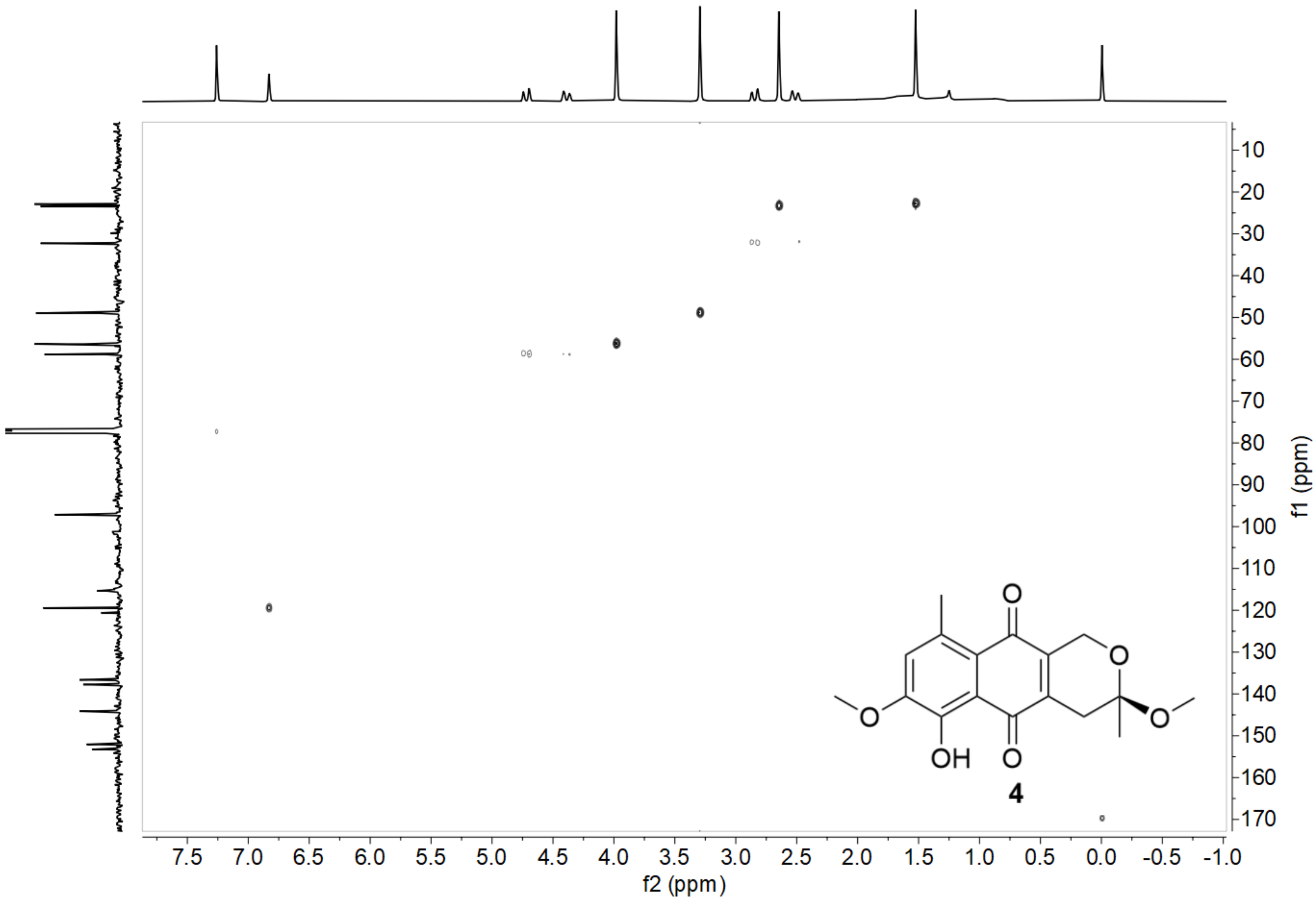


**Figure S4-5.** HMQC spectrum of **4** in $CDCl_3$

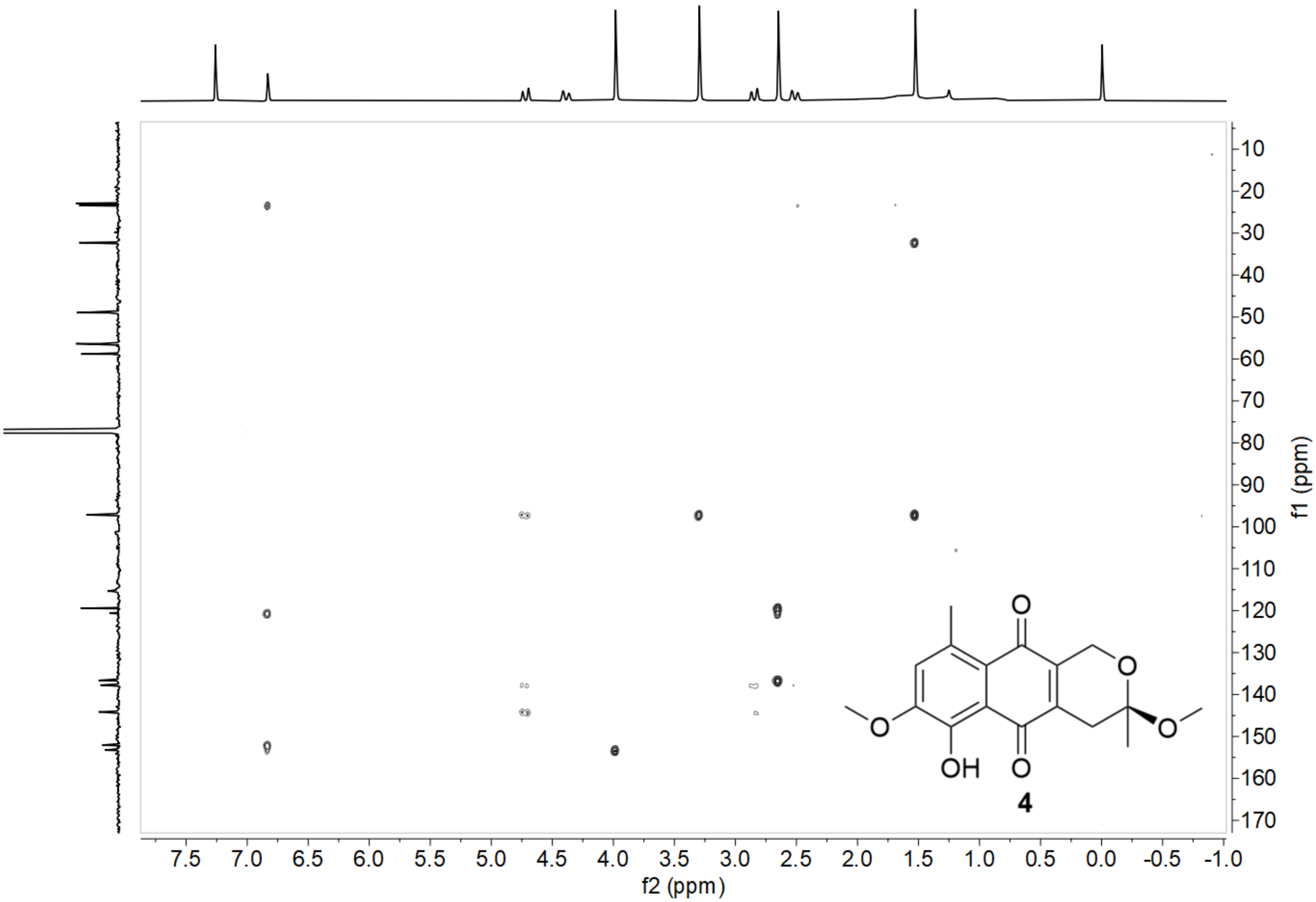


**Figure S4-6.** HMBC spectrum of **4** in $CDCl_3$

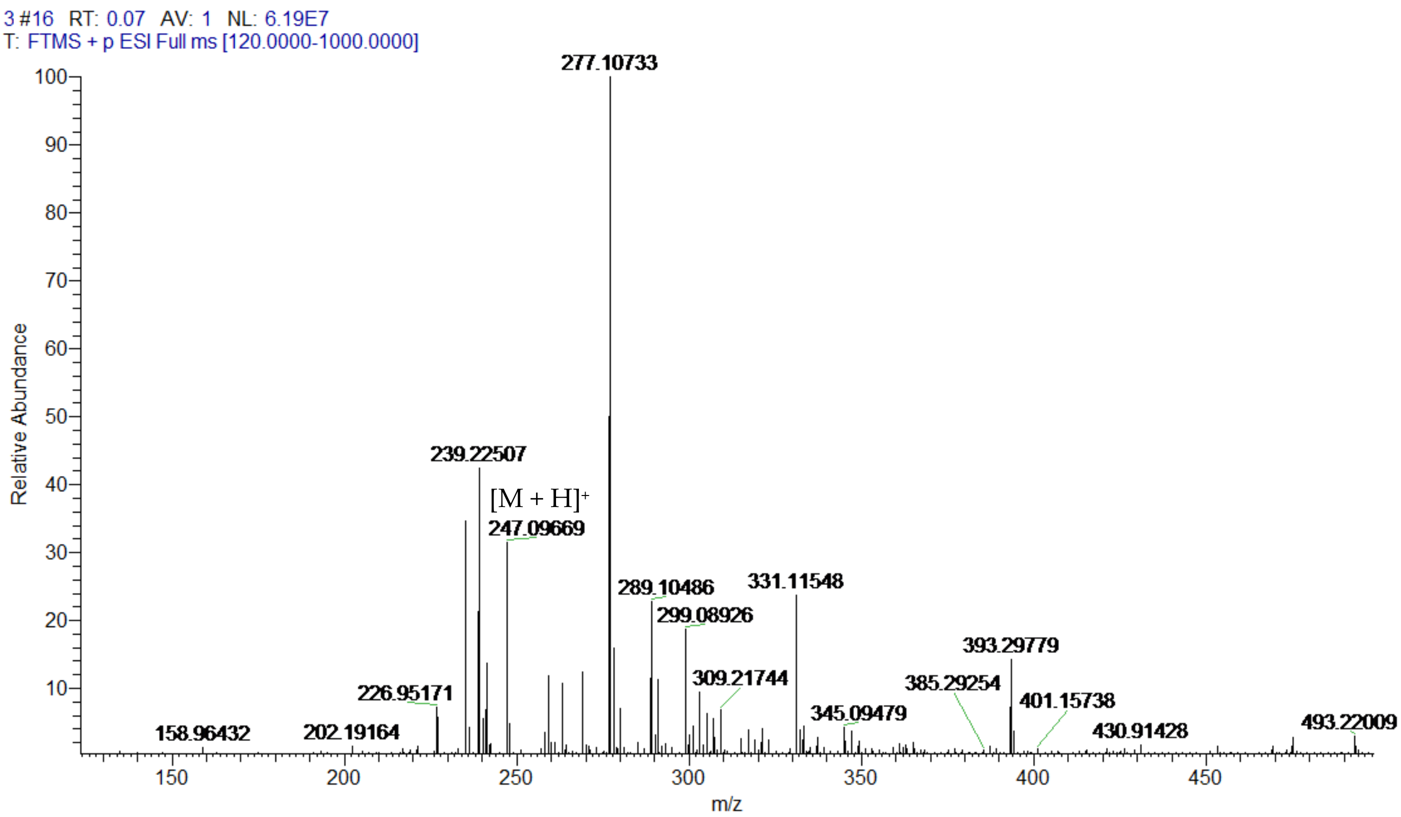


**Figure S5-1.** HRESIMS spectrum of compound **5.**

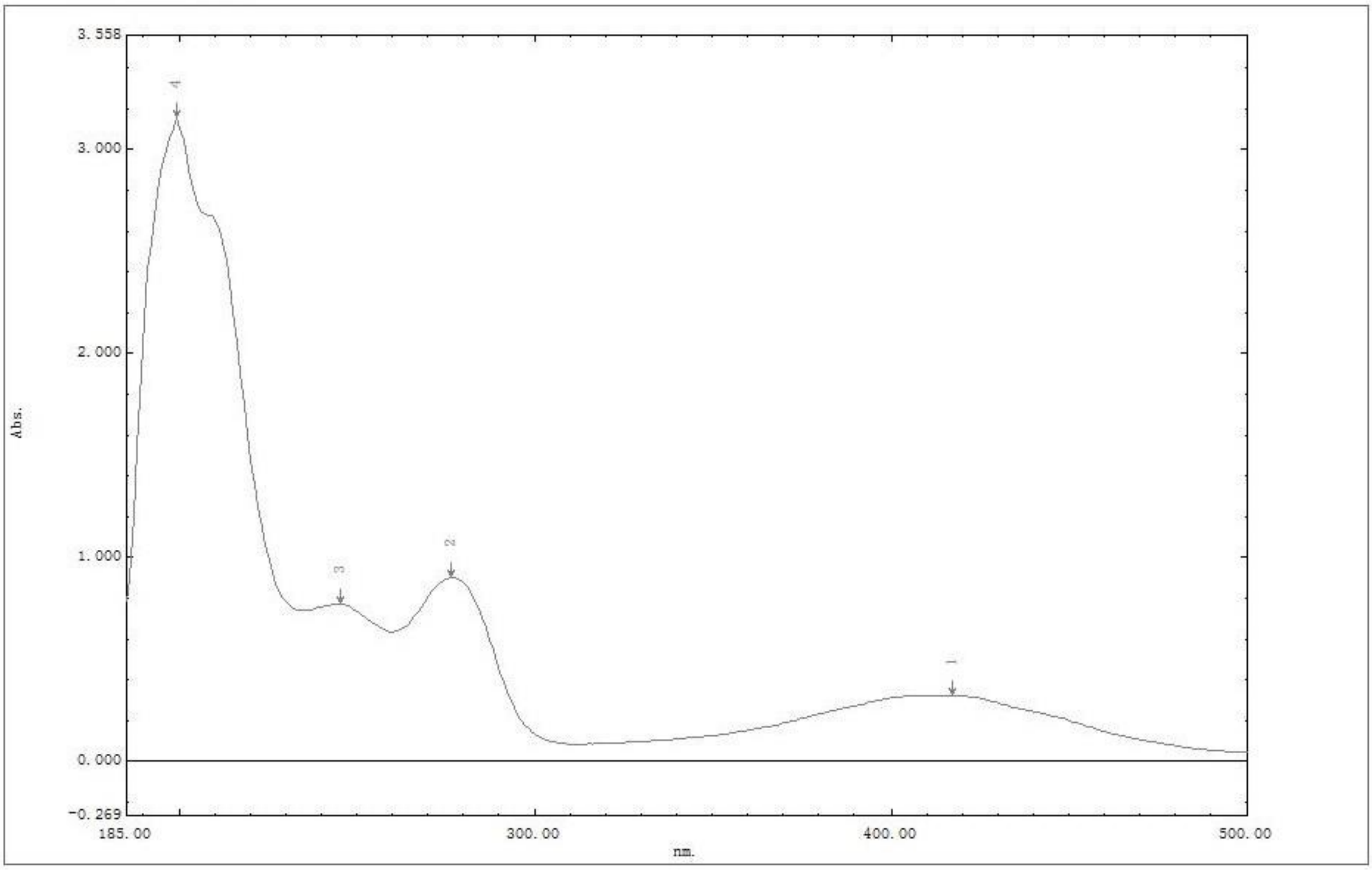


**Figure S5-2.** UV spectrum of compound **5**

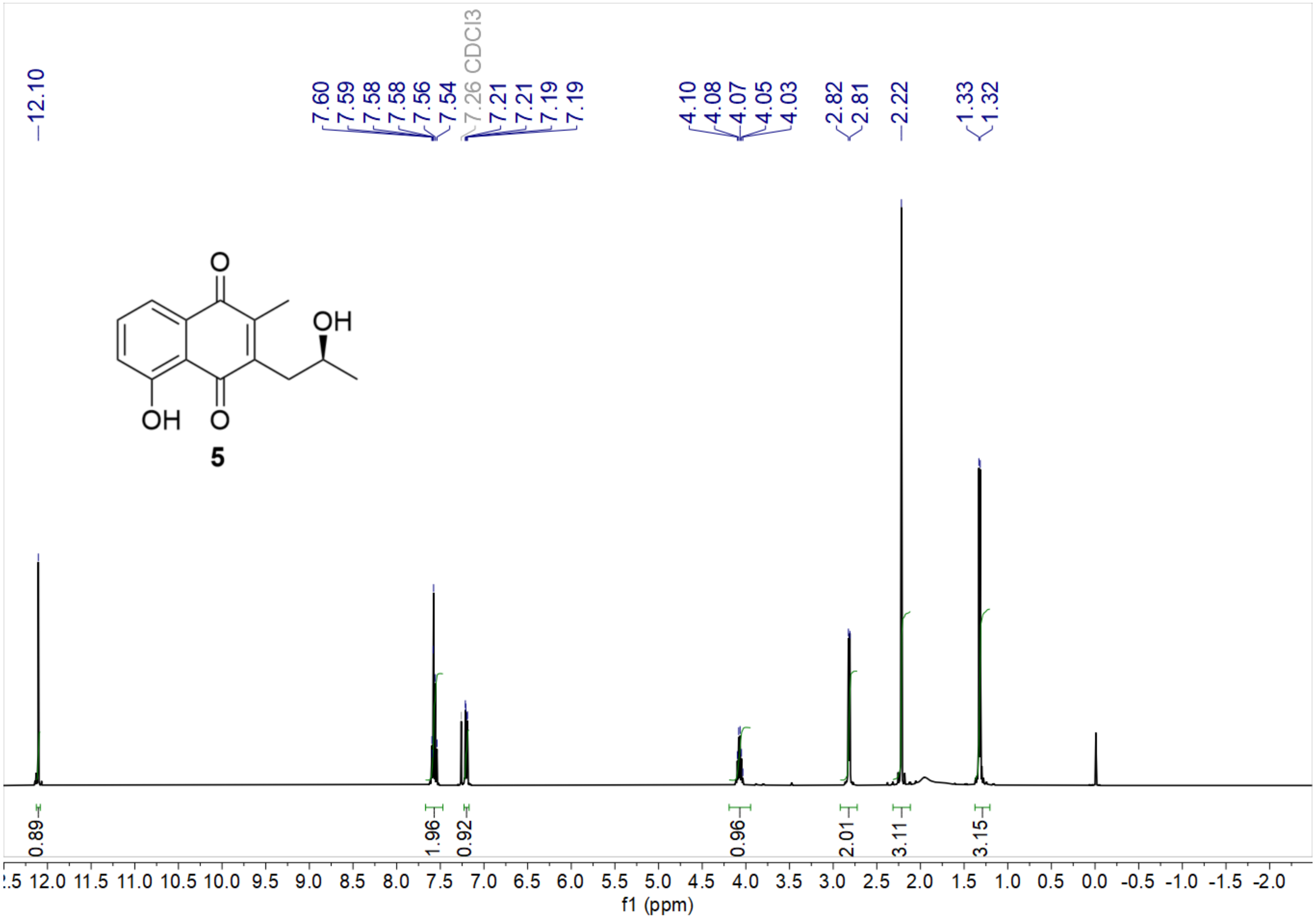


**Figure S5-3.** $^{1}H$ NMR spectrum of **5** in $CDCl_3$ (400 MHz)

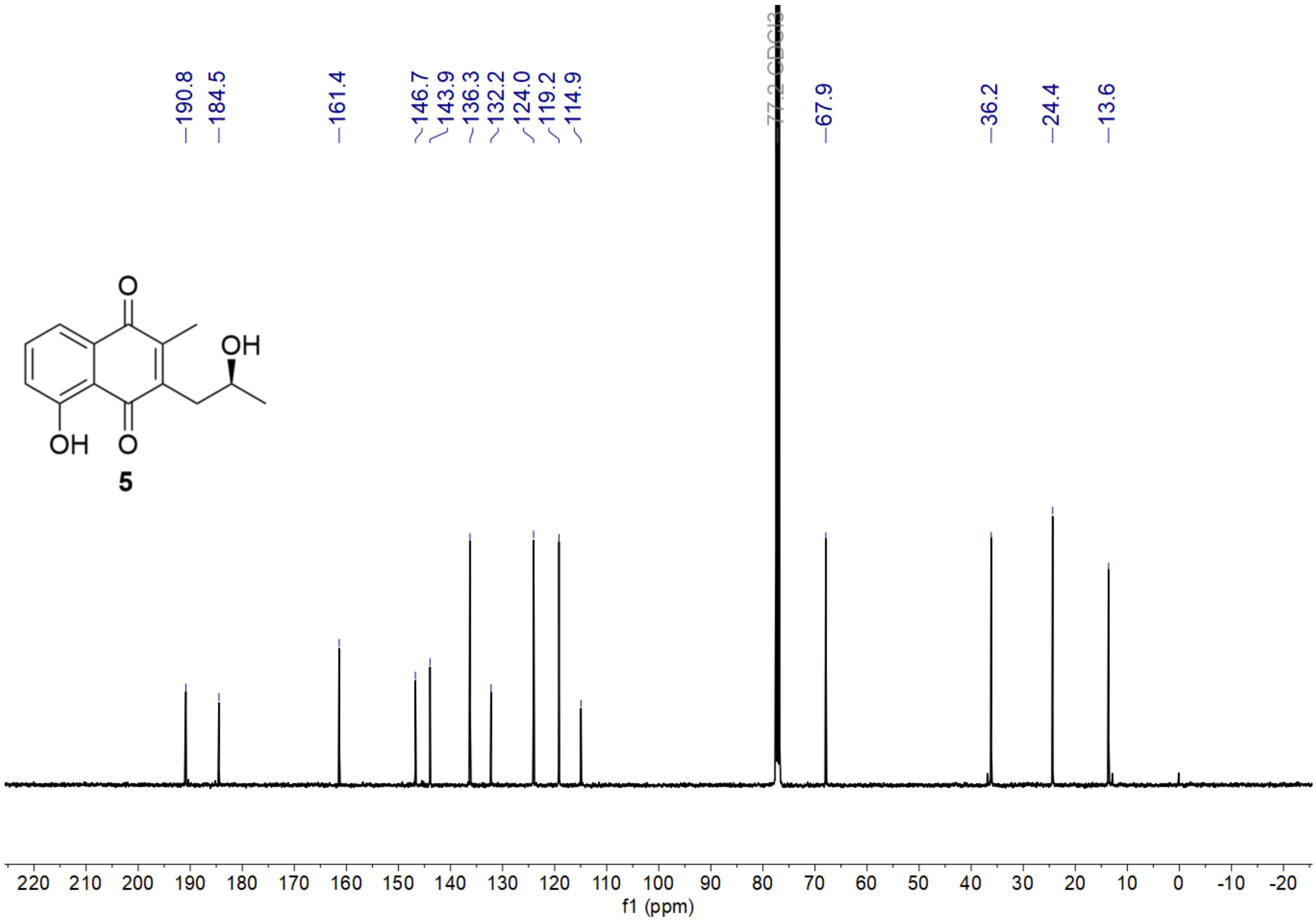


**Figure S5-4.** $^{13}C$ NMR spectrum of **5** in $CDCl_3$ (100 MHz)

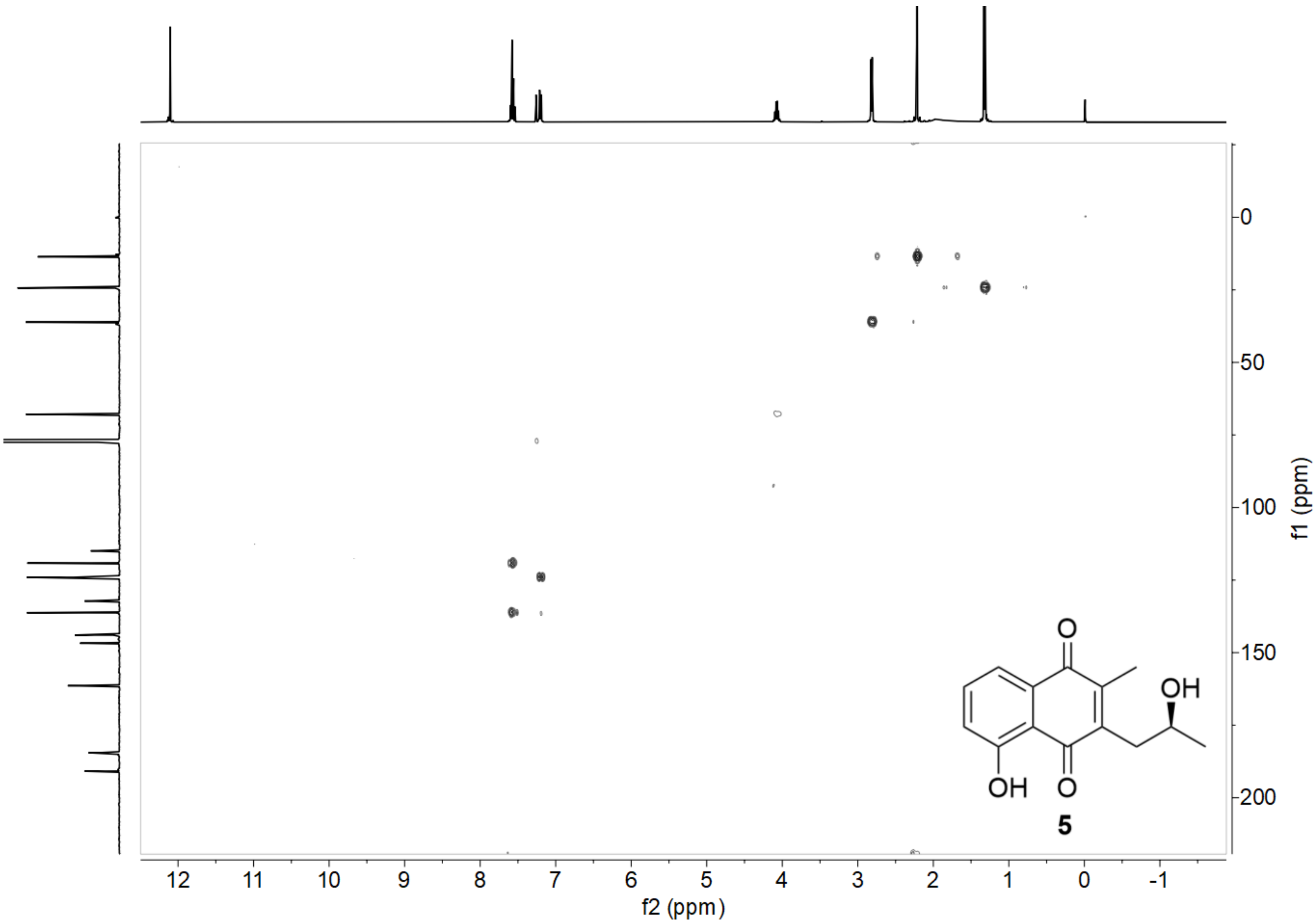


**Figure S5-5.** HMQC spectrum of **5** in $CDCl_3$

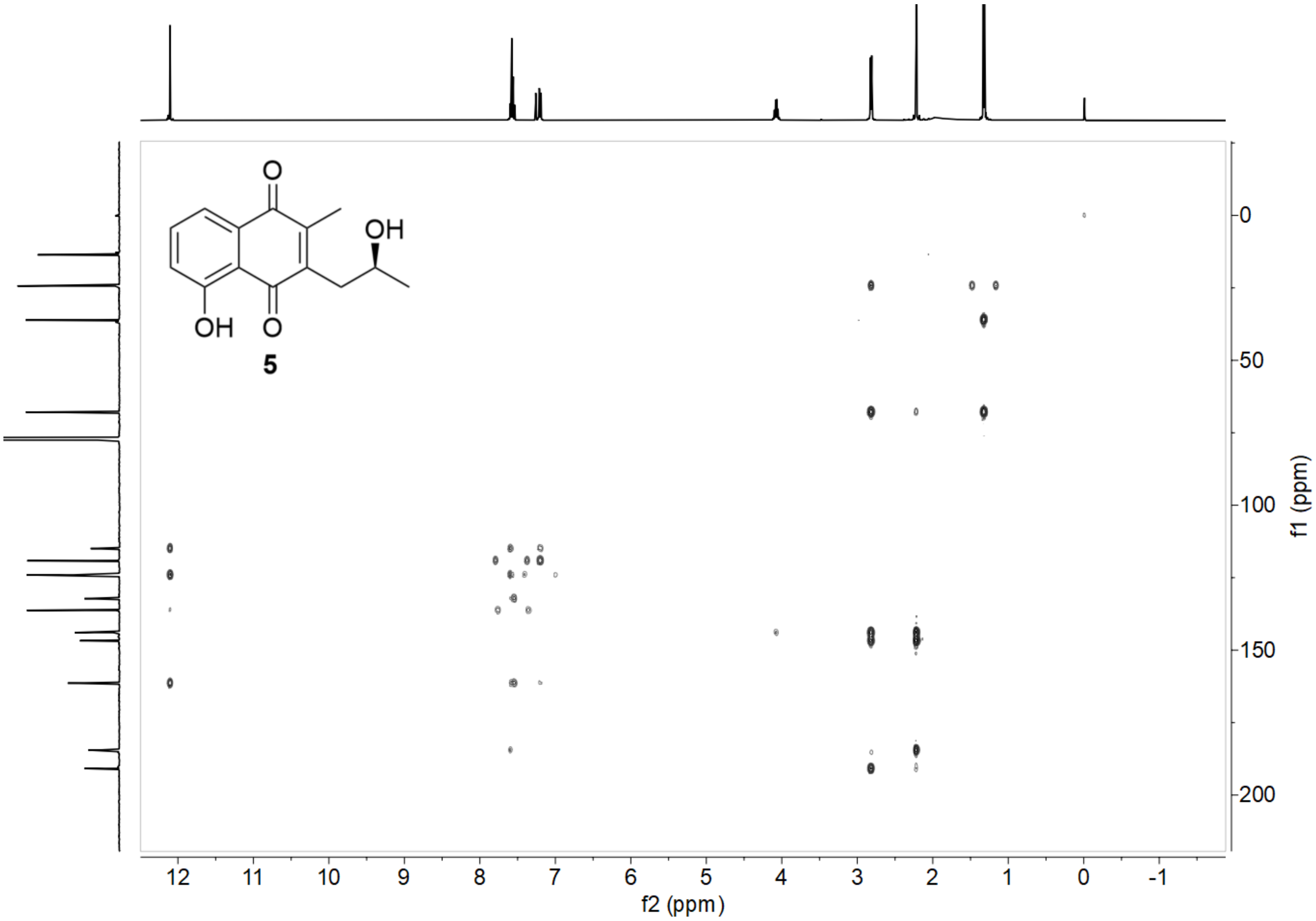


**Figure S5-6.** HMBC spectrum of **5** in $CDCl_3$

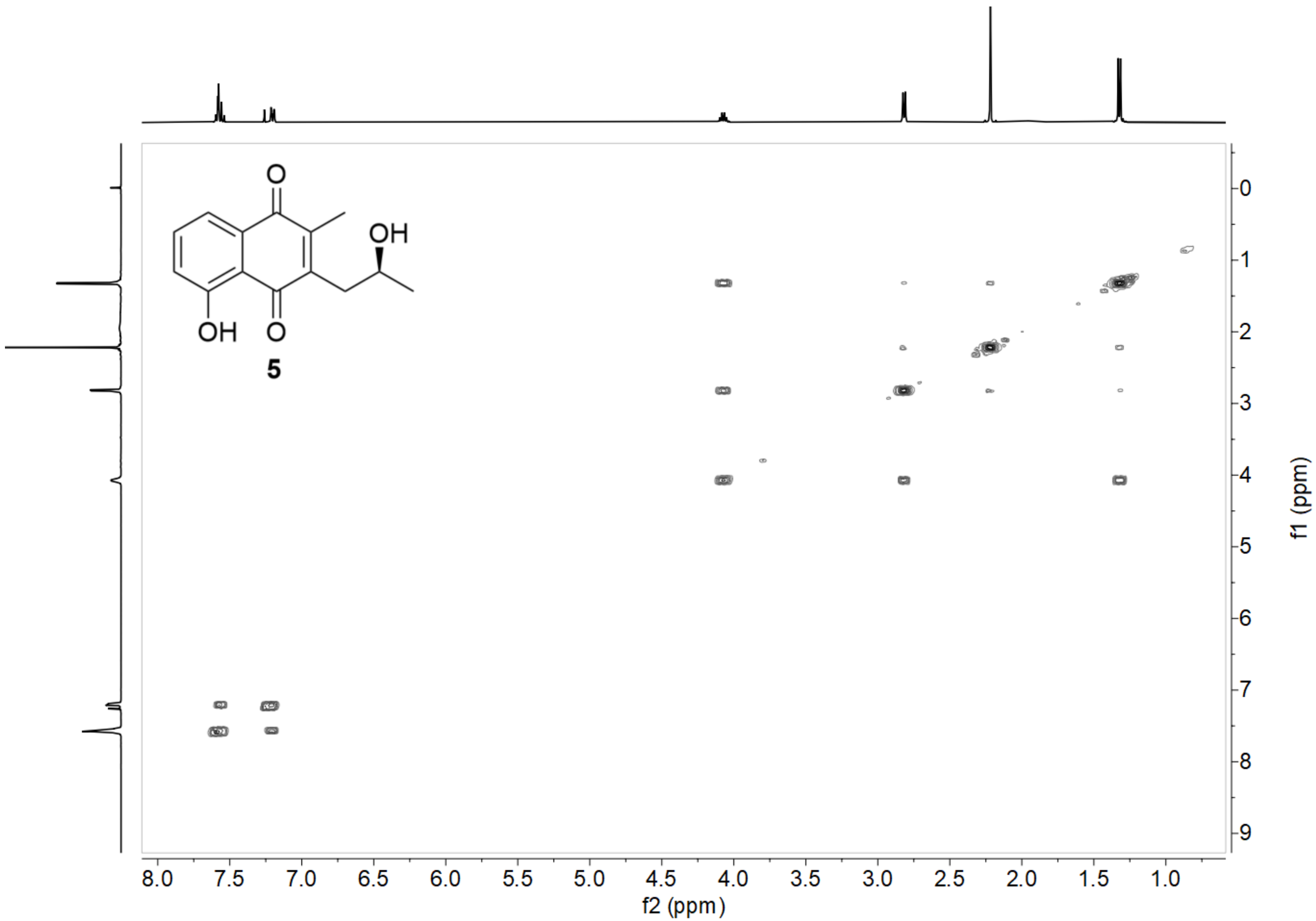


**Figure S5-7.** $^1H$-$^1H$ COSY spectrum of **5** in $CDCl_3$

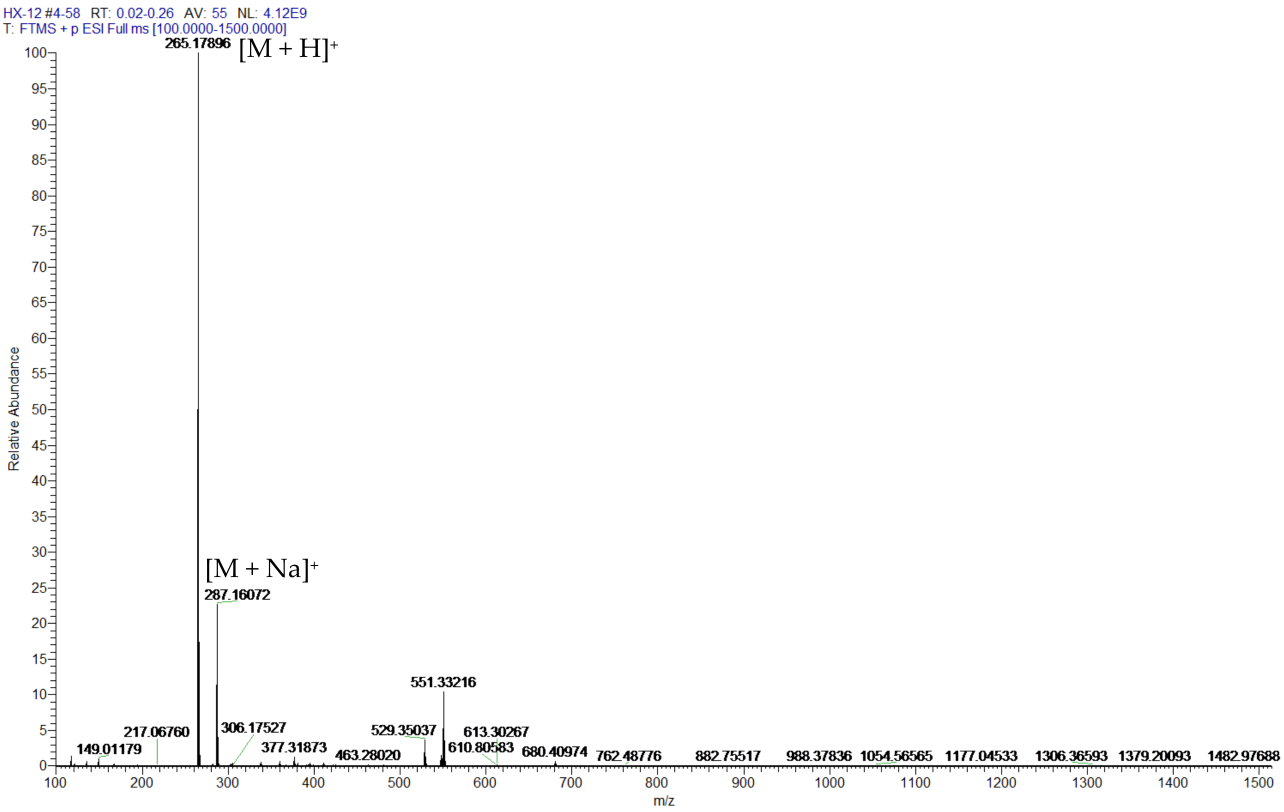


**Figure S6-1.** HRESIMS spectrum of compound **6**

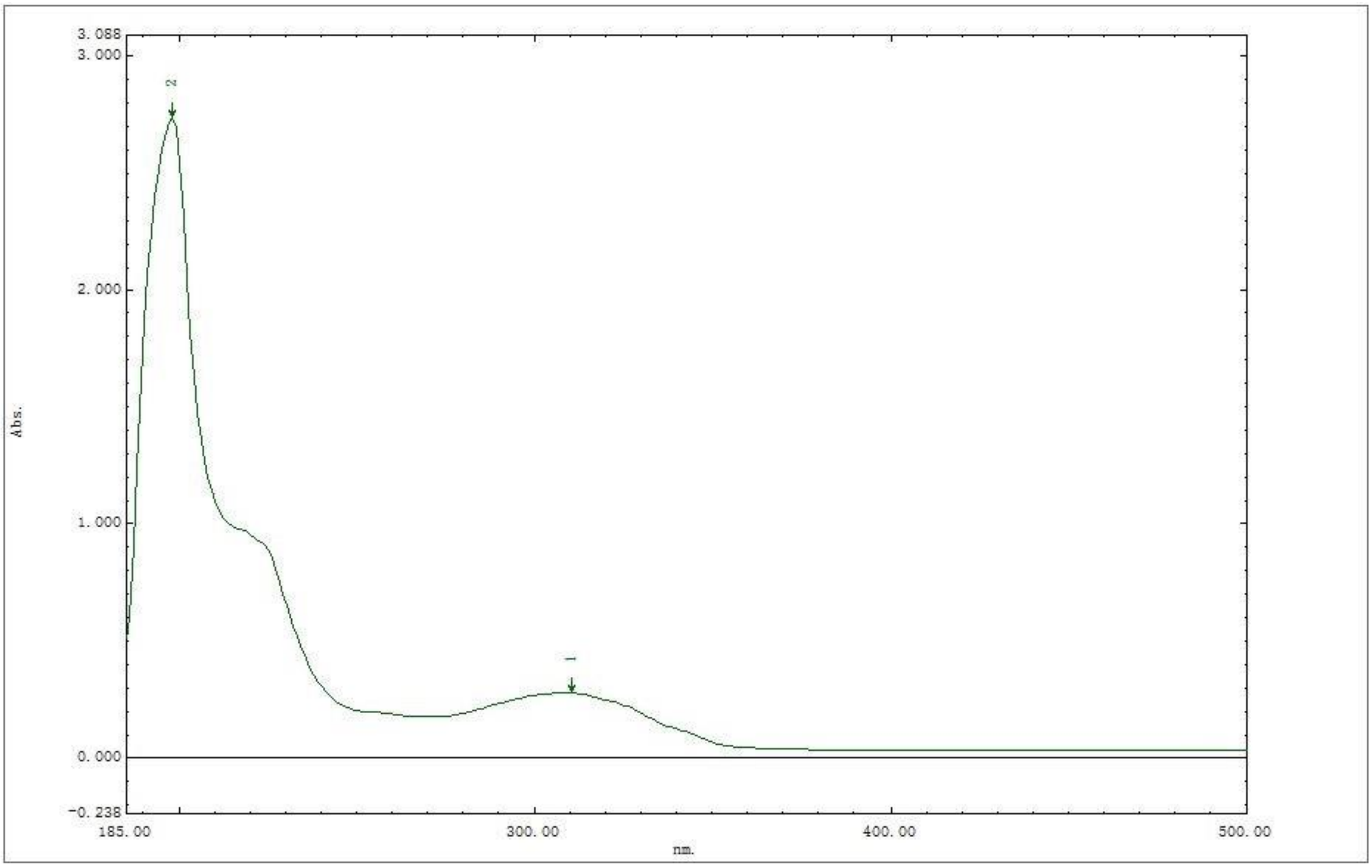


**Figure S6-2.** UV spectrum of compound **6**

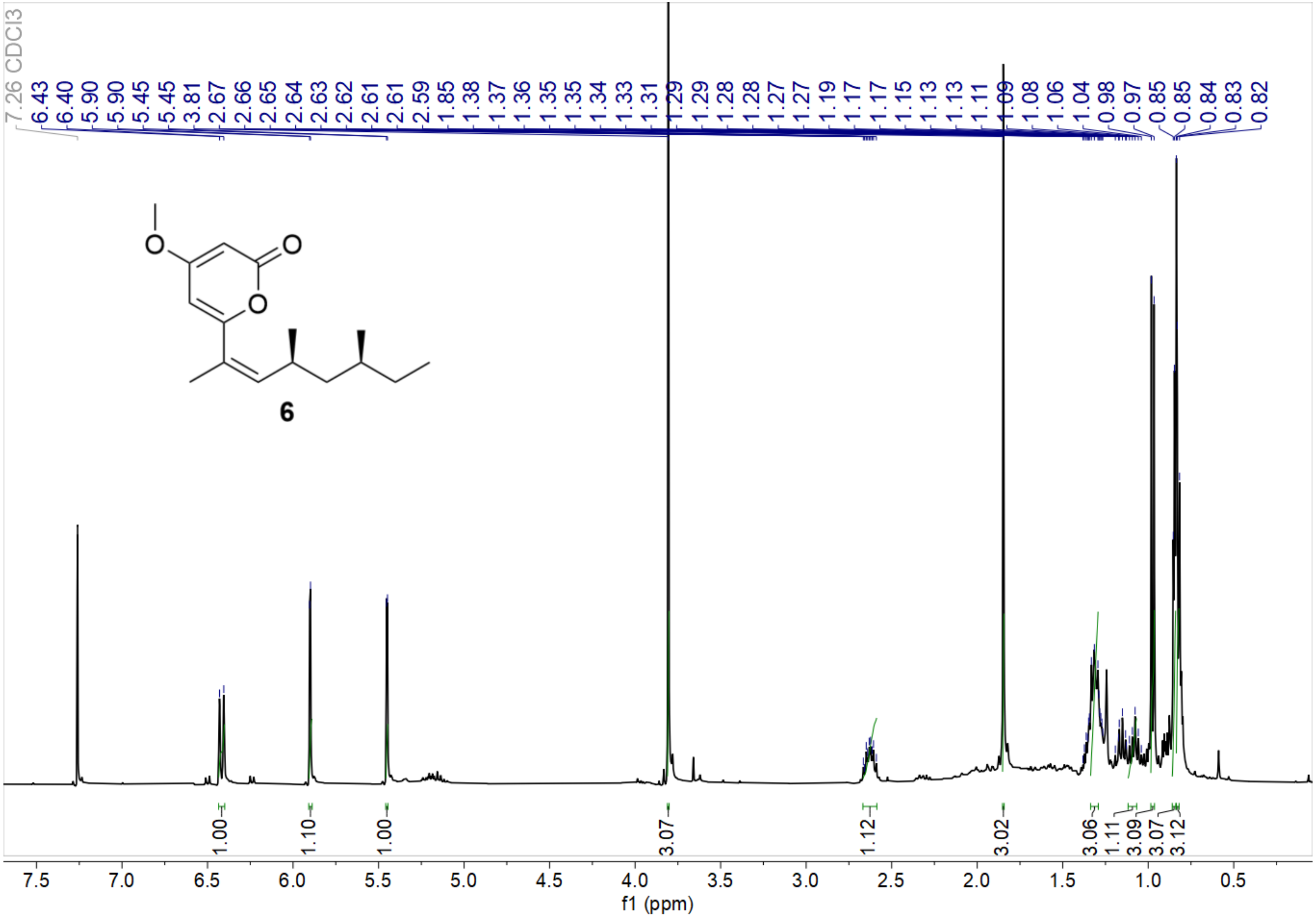


**Figure S6-3.** $^1$H NMR spectrum of **6** in $CDCl_3$ (400 MHz)

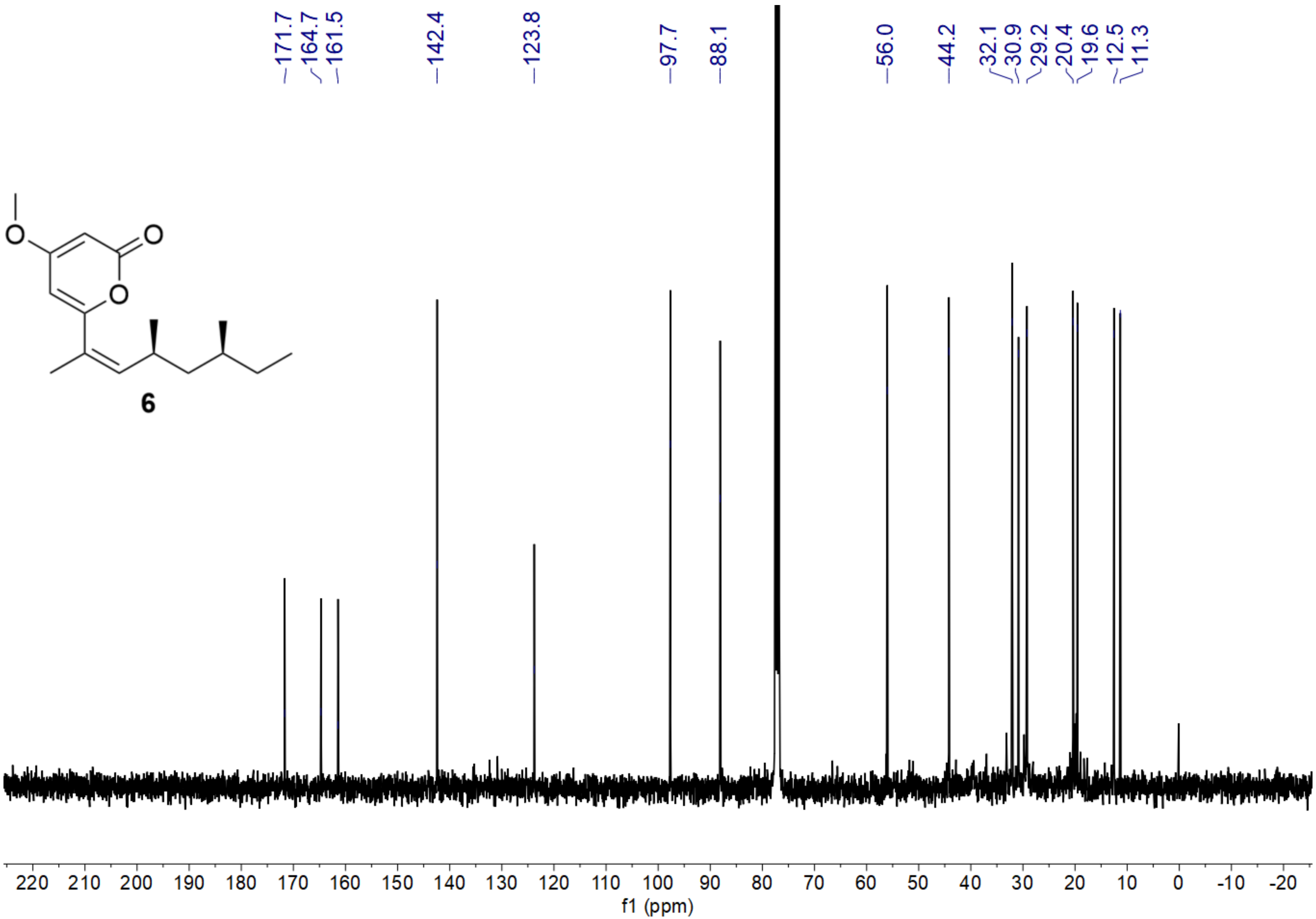


**Figure S6-4.** $^{13}C$ spectrum of **6** in $CDCl_3$ (100 MHz)

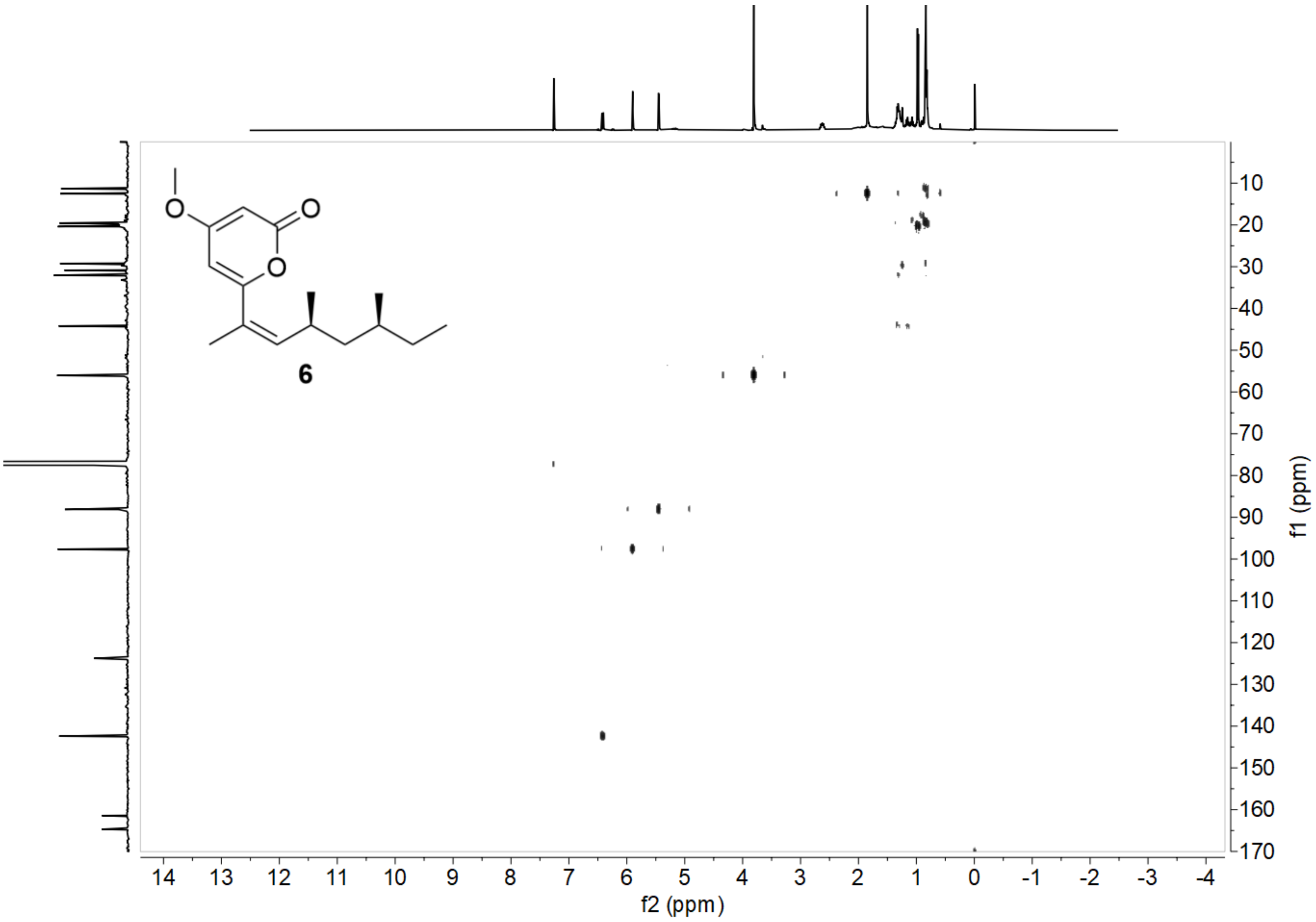


**Figure S6-5.** HMQC spectrum of **6** in $CDCl_3$

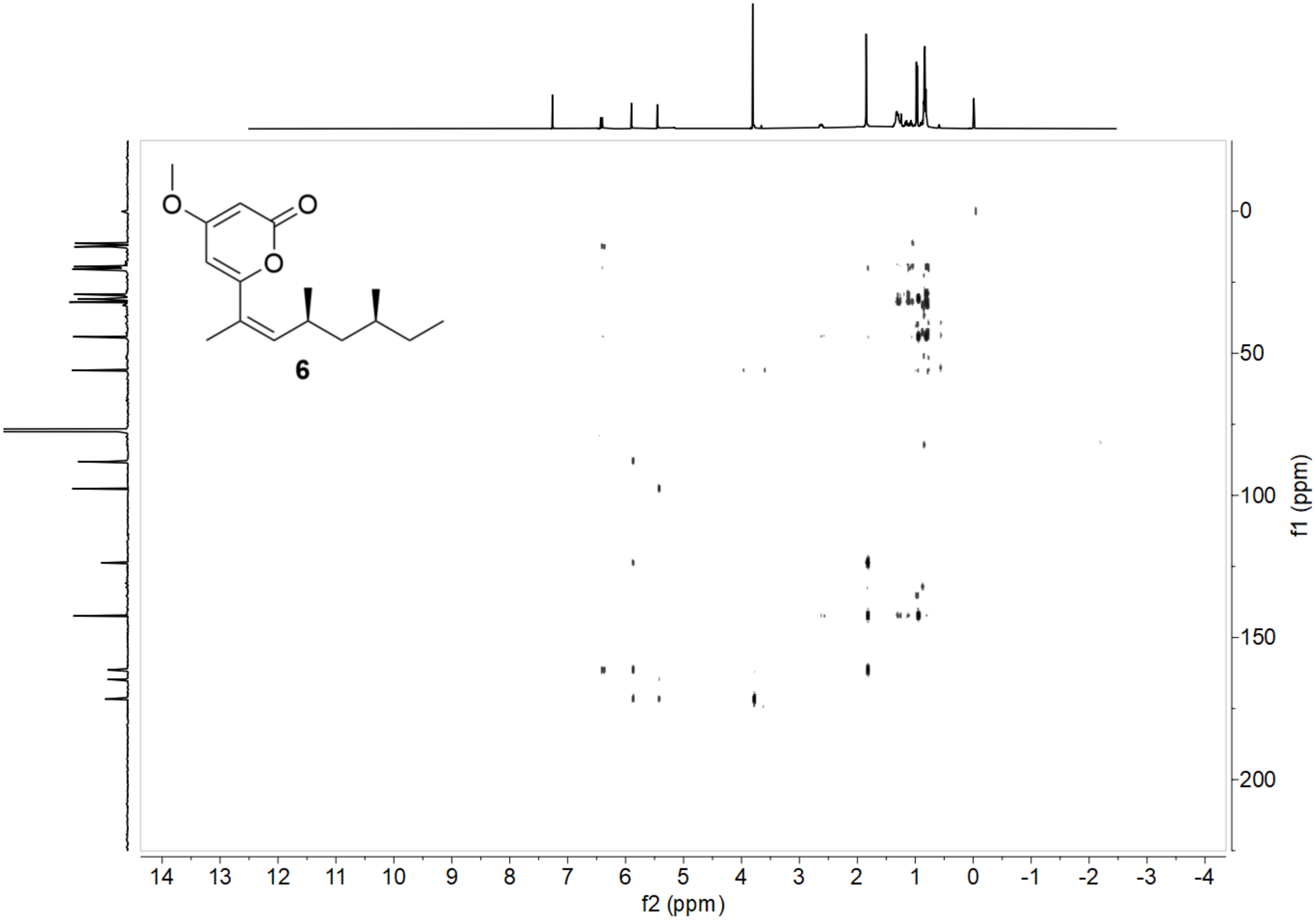


**Figure S6-6.** HMBC spectrum of **6** in $CDCl_3$

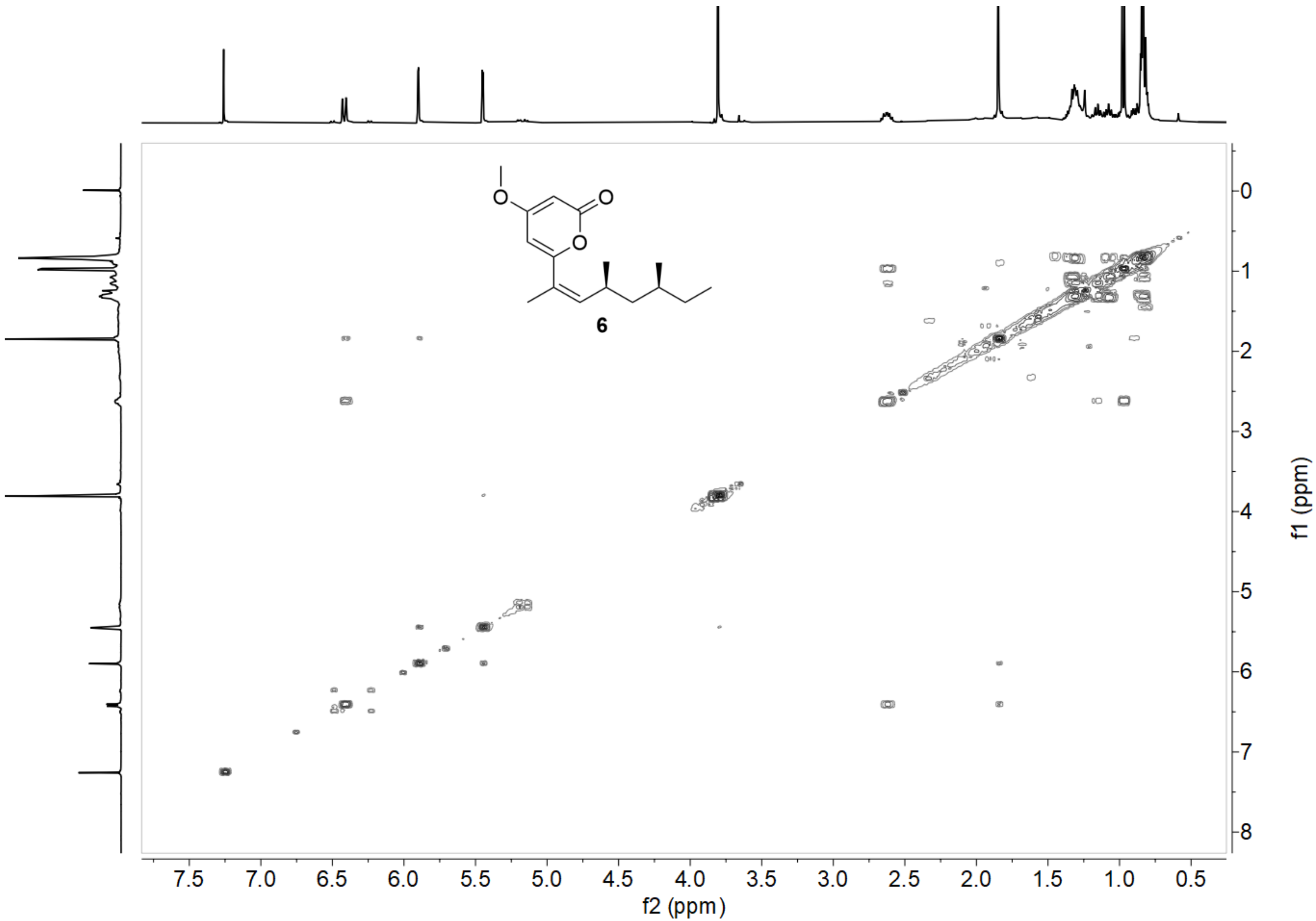


**Figure S6-7.** $^1H$-$^1H$ COSY spectrum of **6** in $CDCl_3$

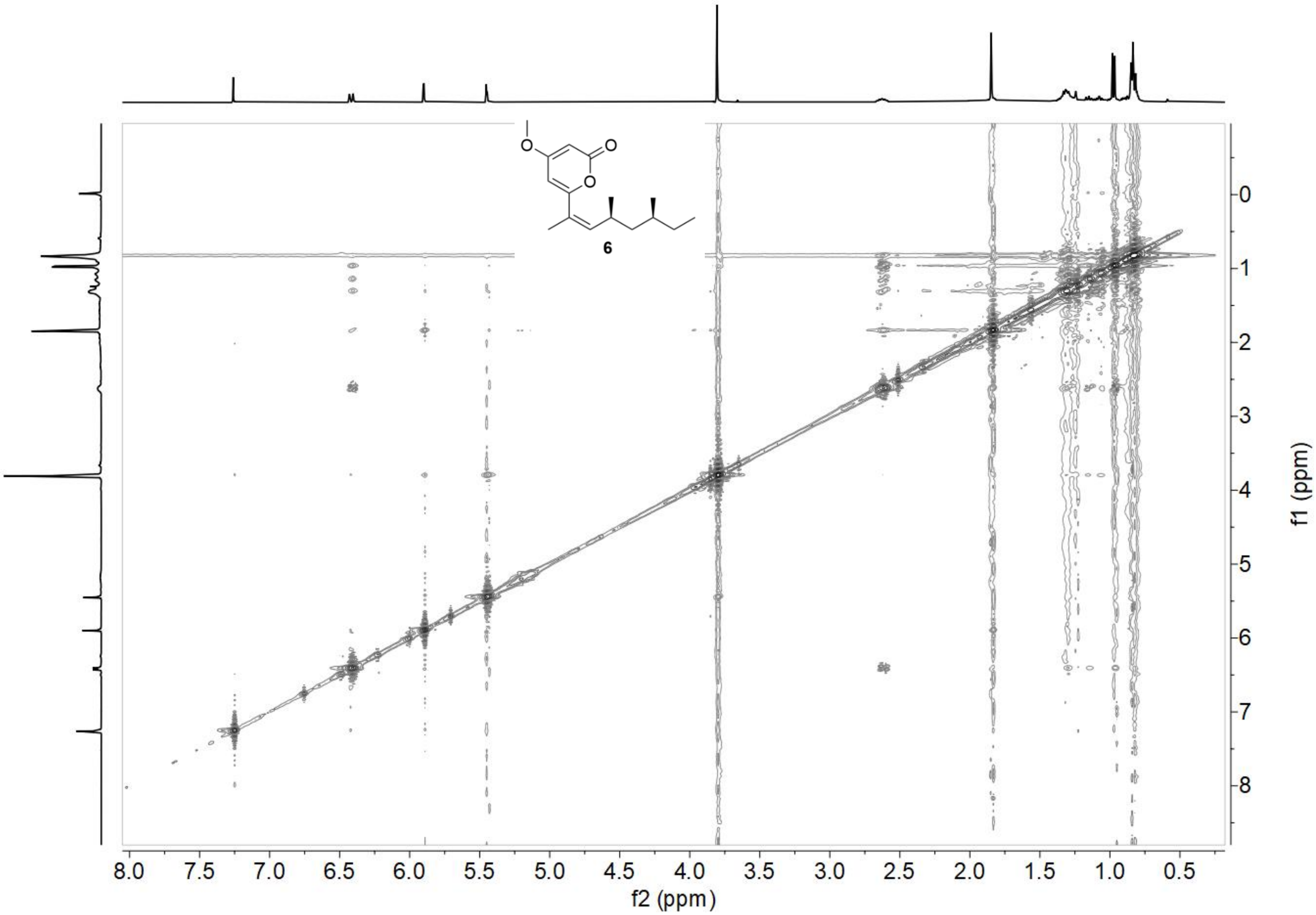


**Figure S6-8.** NOESY spectrum of **6** in $CDCl_3$

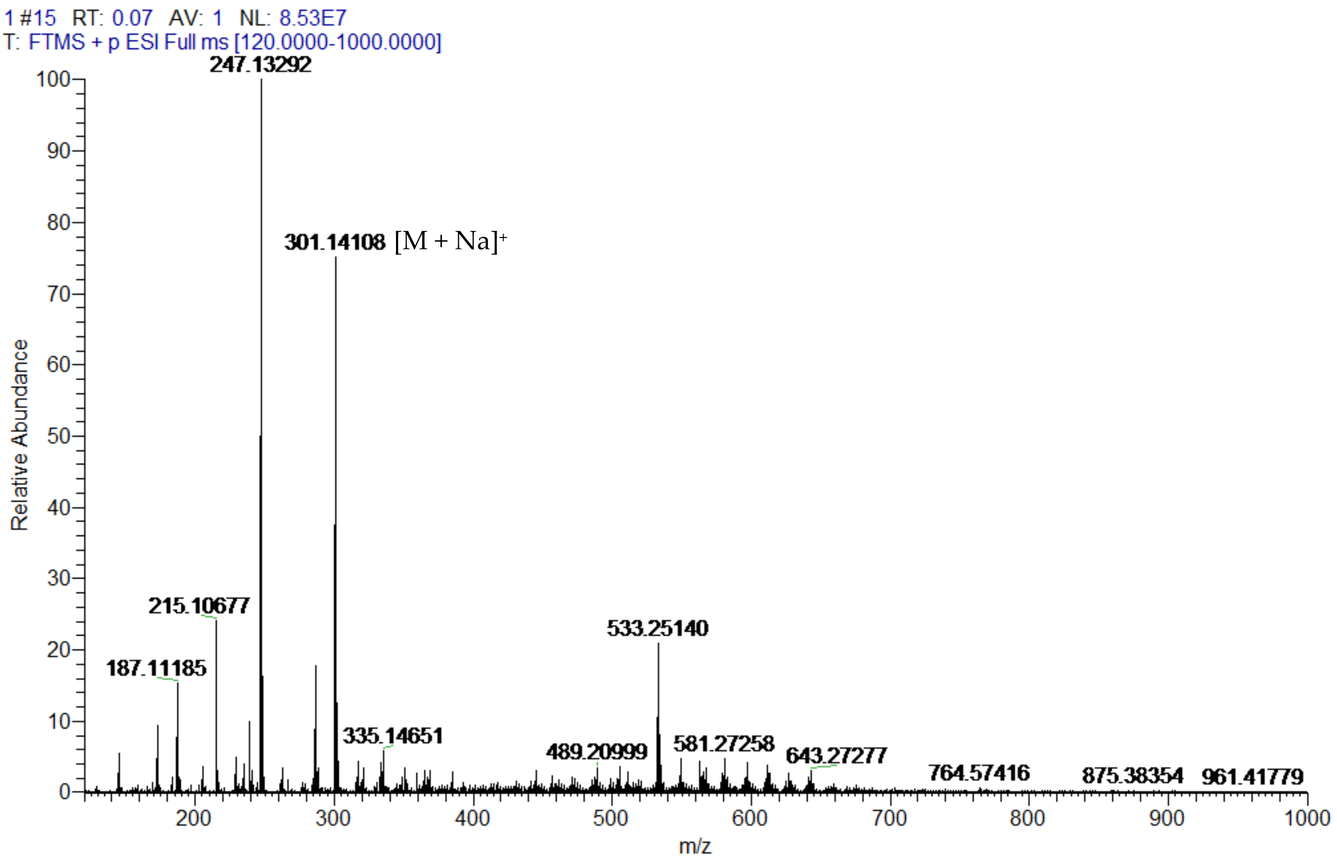


**Figure S7-1.** HRESIMS spectrum of compound **7**

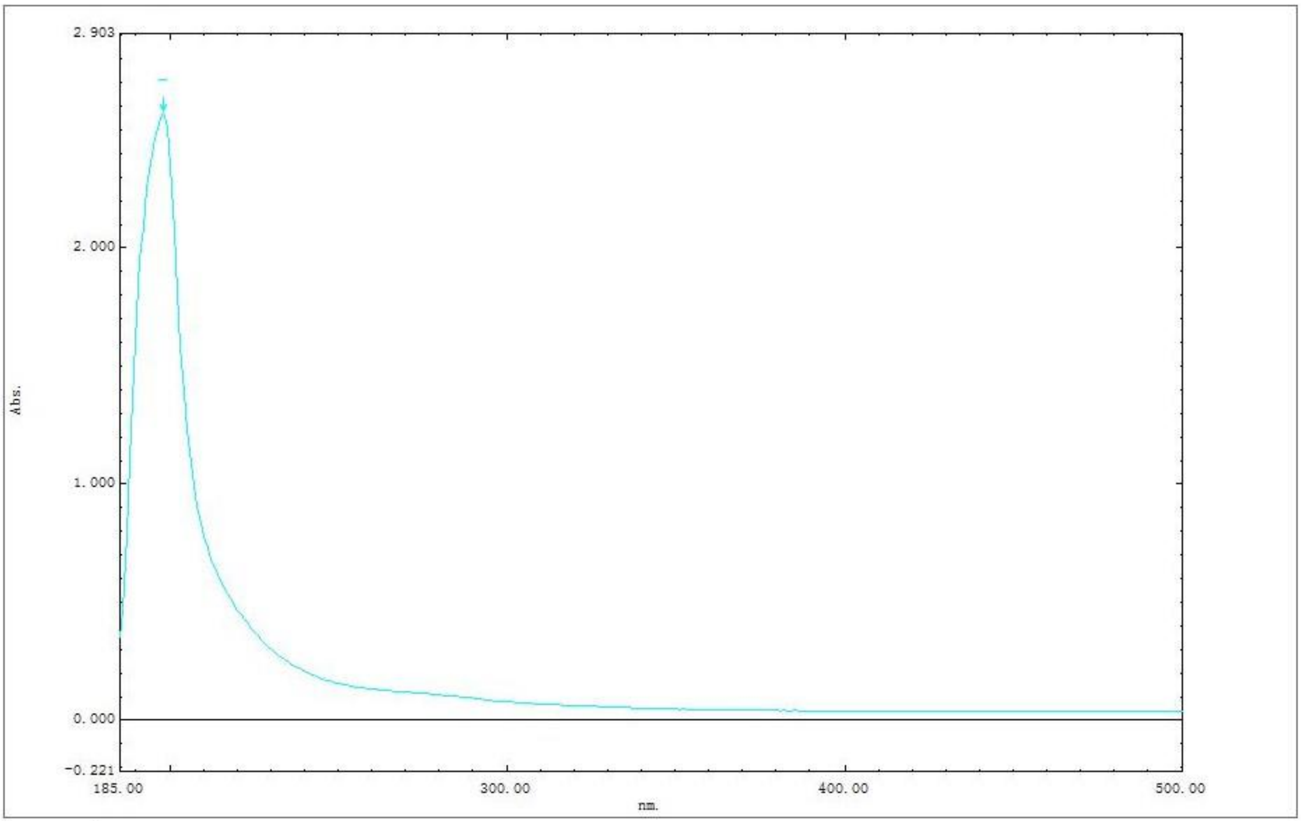


**Figure S7-2.** UV spectrum of compound **7**

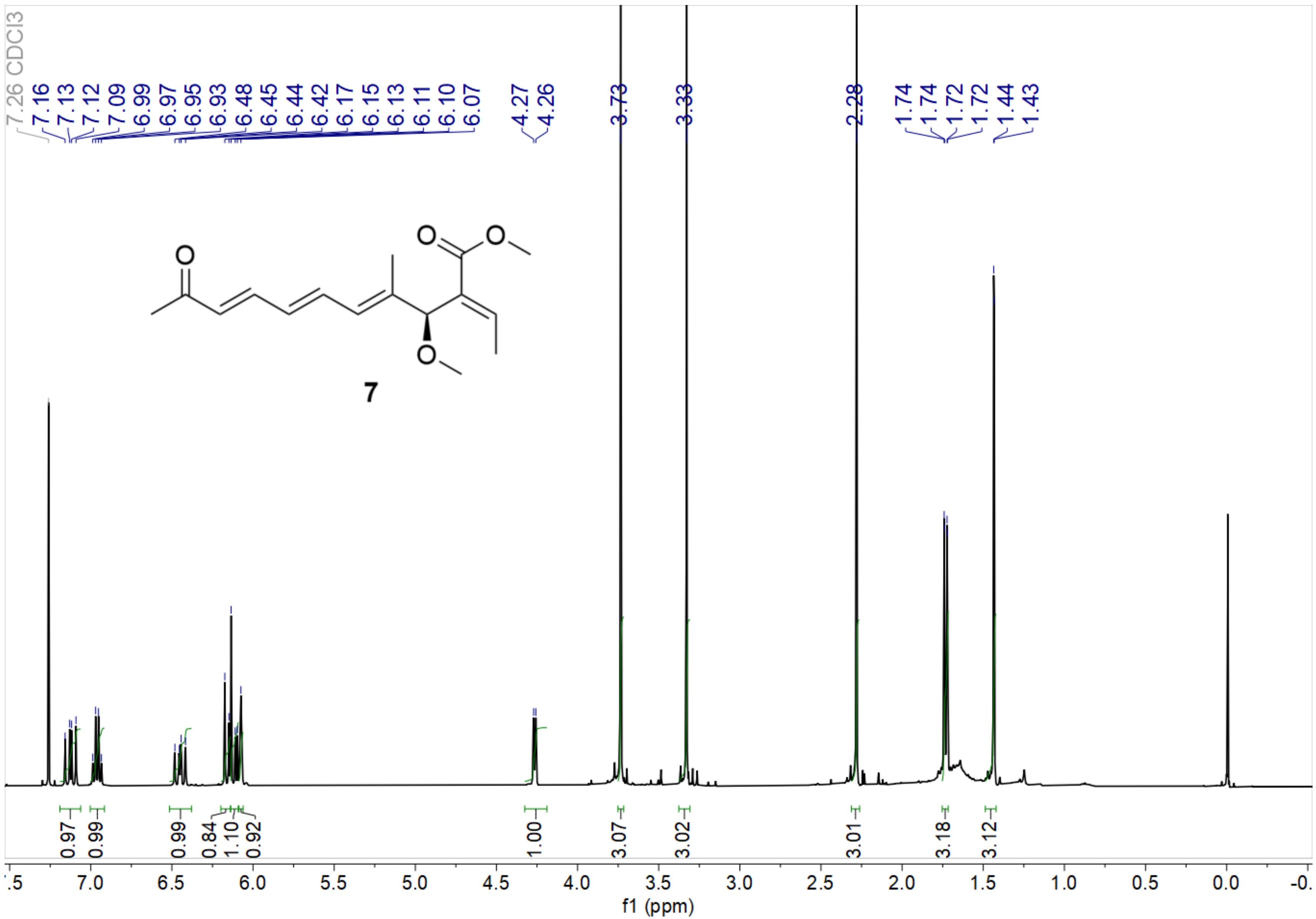


**Figure S7-3.** $^1$H NMR spectrum of **7** in $CDCl_3$ (400 MHz)

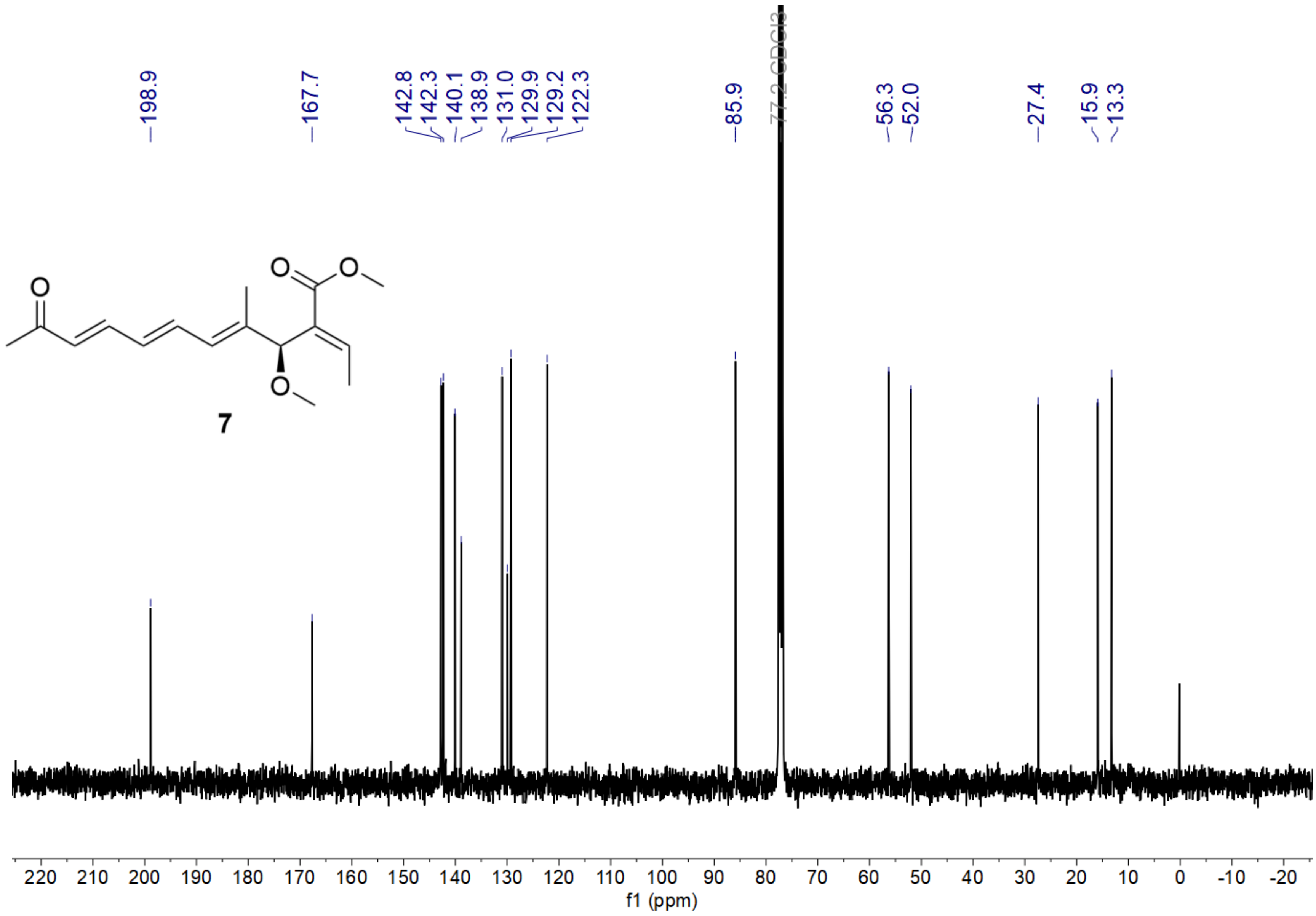


**Figure S7-4.** $^{13}C$ NMR spectrum of **7** in $CDCl_3$ (100 MHz)

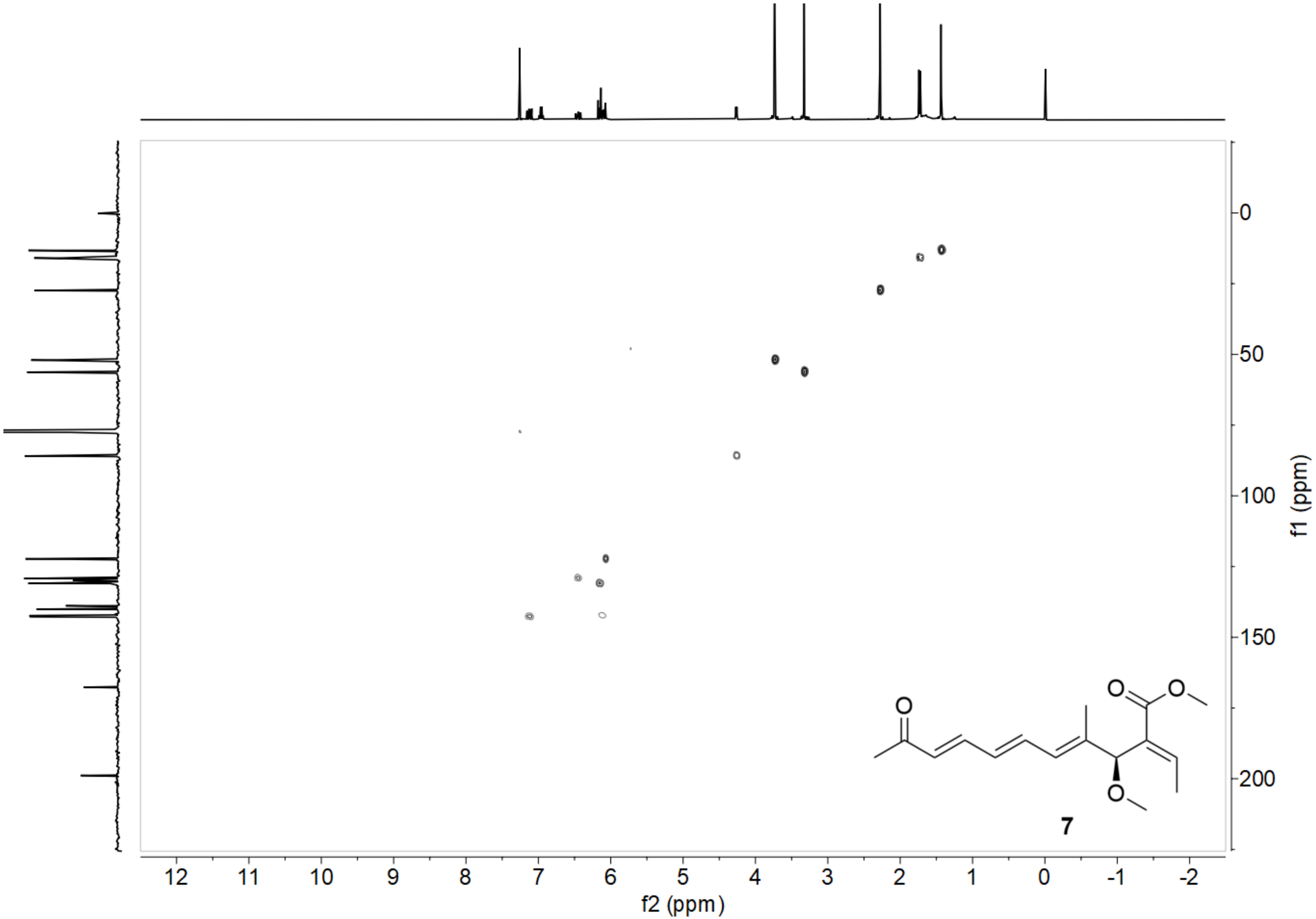


**Figure S7-5.** HMQC spectrum of **7** in $CDCl_3$

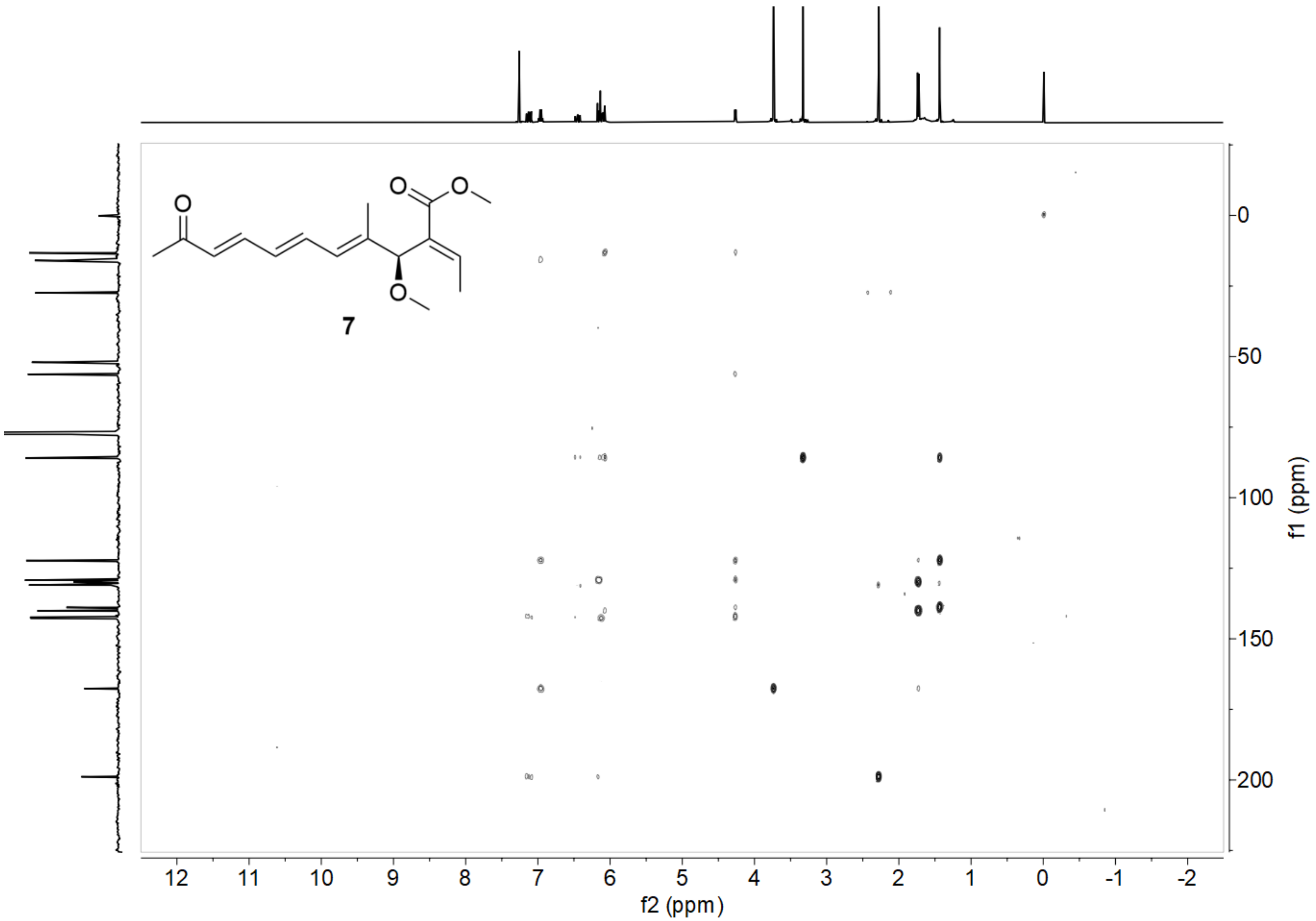


**Figure S7-6.** HMBC spectrum of **7** in $CDCl_3$

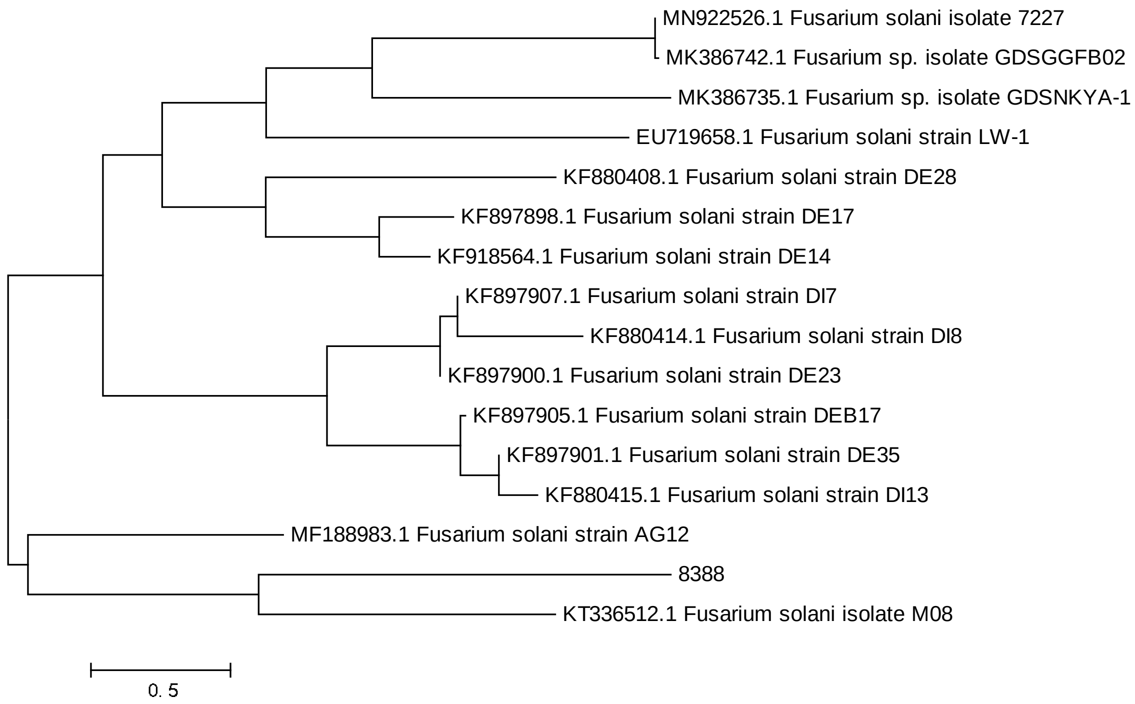


**Figure S8.** Phylogenic tree of marine-derived fungus 8388 constructed by MEGA 5.10

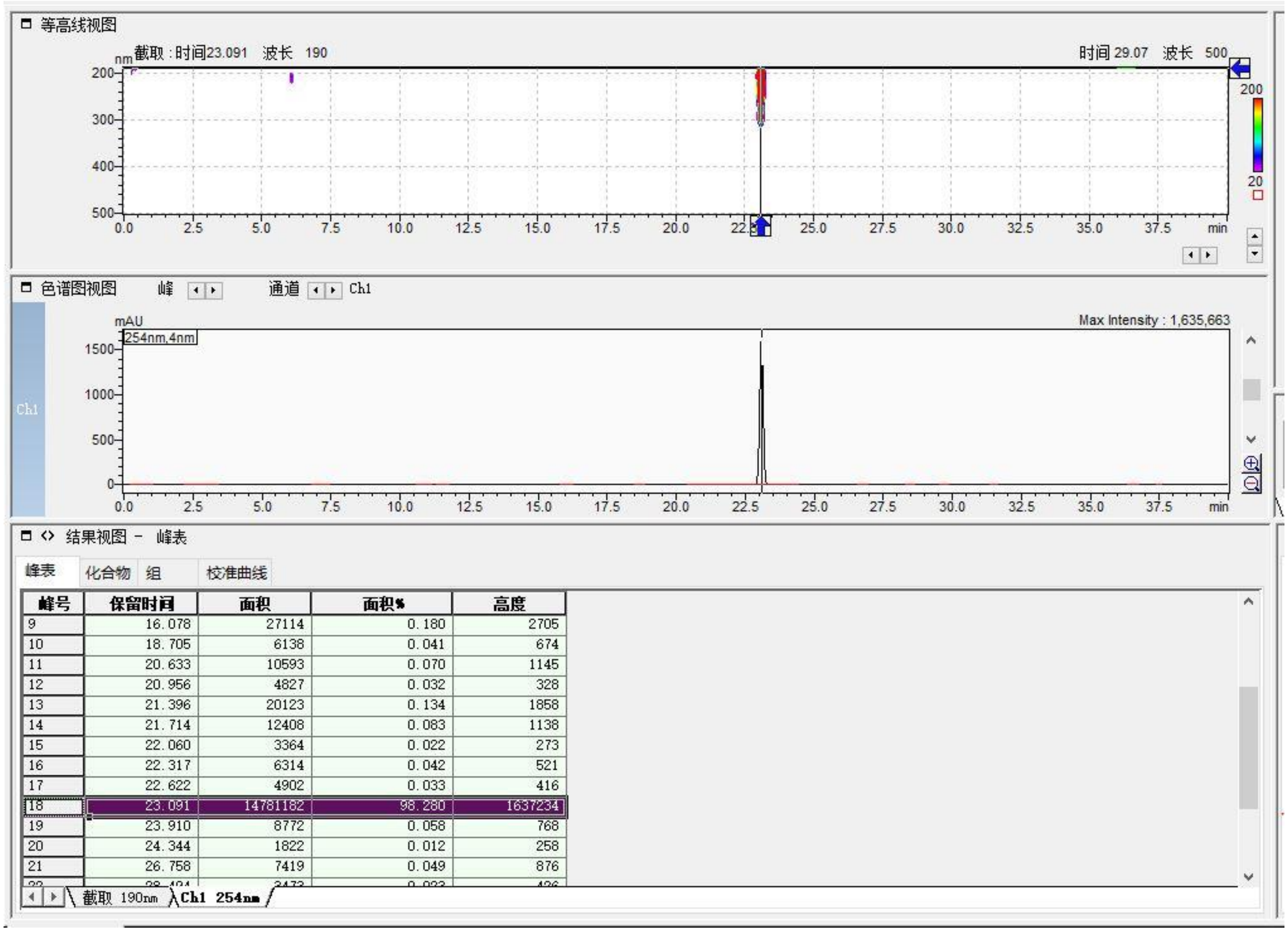


| 峰号 | 保留时间 | 面积 | 面积% | 高度 |
|---|---|---|---|---|
| 9 | 16.078 | 27114 | 0.180 | 2705 |
| 10 | 18.705 | 6138 | 0.041 | 674 |
| 11 | 20.633 | 10593 | 0.070 | 1145 |
| 12 | 20.956 | 4827 | 0.032 | 328 |
| 13 | 21.396 | 20123 | 0.134 | 1858 |
| 14 | 21.714 | 12408 | 0.083 | 1138 |
| 15 | 22.060 | 3364 | 0.022 | 273 |
| 16 | 22.317 | 6314 | 0.042 | 521 |
| 17 | 22.622 | 4902 | 0.033 | 416 |
| 18 | 23.091 | 14781182 | 98.280 | 1637234 |
| 19 | 23.910 | 8772 | 0.058 | 768 |
| 20 | 24.344 | 1822 | 0.012 | 258 |
| 21 | 26.758 | 7419 | 0.049 | 876 |
| [illegible] | [illegible] | [illegible] | [illegible] | [illegible] |

**Figure S9.** High performance liquid chromatography (HPLC) analysis of compound **1**

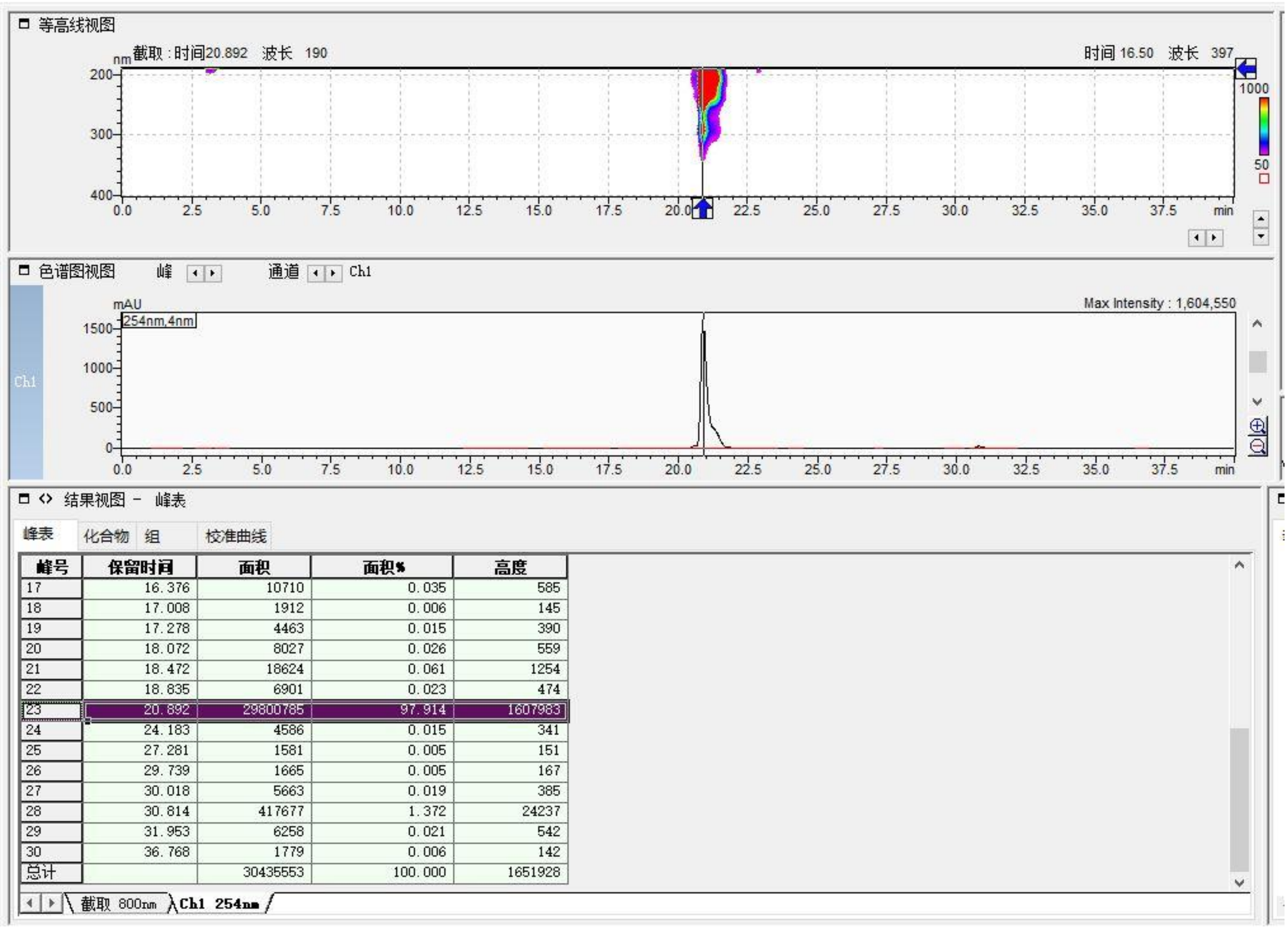


| 峰号 | 保留时间 | 面积 | 面积% | 高度 |
|---|---|---|---|---|
| 17 | 16.376 | 10710 | 0.035 | 585 |
| 18 | 17.008 | 1912 | 0.006 | 145 |
| 19 | 17.278 | 4463 | 0.015 | 390 |
| 20 | 18.072 | 8027 | 0.026 | 559 |
| 21 | 18.472 | 18624 | 0.061 | 1254 |
| 22 | 18.835 | 6901 | 0.023 | 474 |
| 23 | 20.892 | 29800785 | 97.914 | 1607983 |
| 24 | 24.183 | 4586 | 0.015 | 341 |
| 25 | 27.281 | 1581 | 0.005 | 151 |
| 26 | 29.739 | 1665 | 0.005 | 167 |
| 27 | 30.018 | 5663 | 0.019 | 385 |
| 28 | 30.814 | 417677 | 1.372 | 24237 |
| 29 | 31.953 | 6258 | 0.021 | 542 |
| 30 | 36.768 | 1779 | 0.006 | 142 |
| 总计 | | 30435553 | 100.000 | 1651928 |

**Figure S10.** High performance liquid chromatography (HPLC) analysis of compound **2**

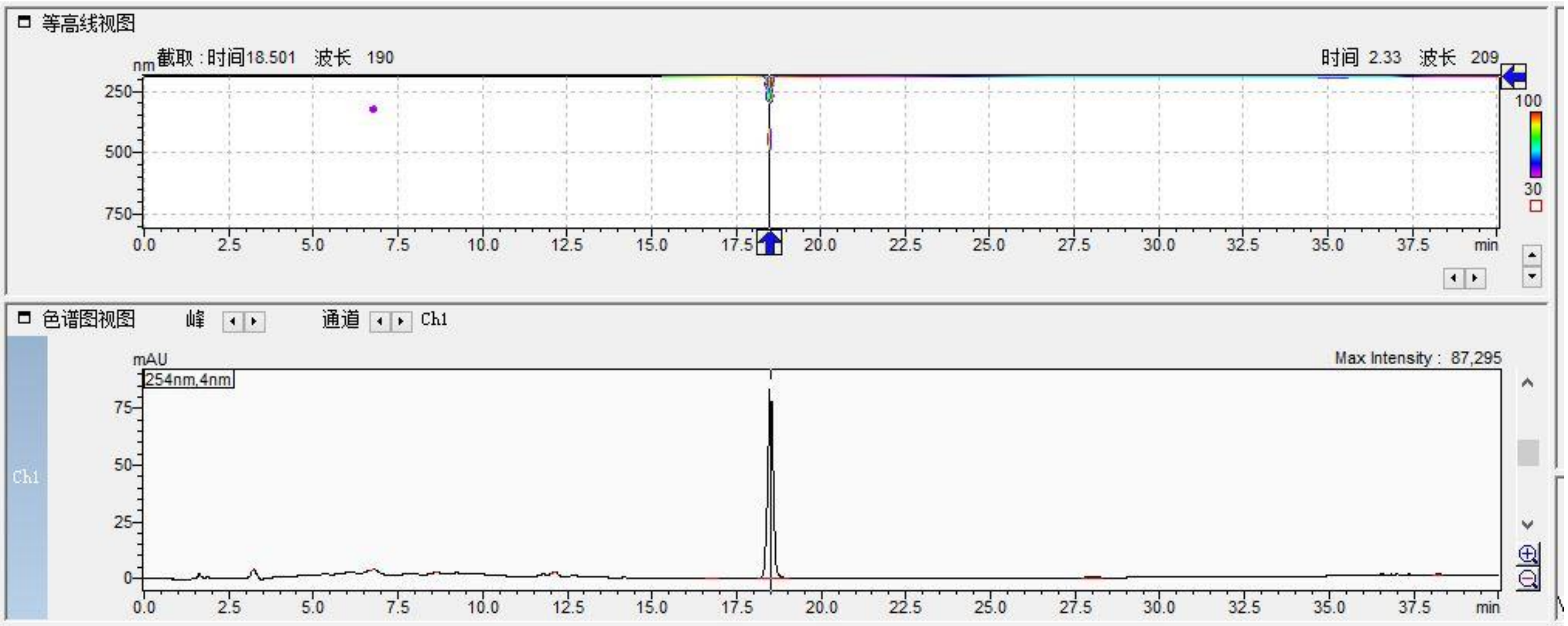


结果视图 - 峰表

峰表 | 化合物 | 组 | 校准曲线

| 峰号 | 保留时间 | 面积 | 面积% | 高度 |
|---|---|---|---|---|
| 1 | 3.258 | 1302 | 0.133 | 742 |
| 2 | 6.792 | 554 | 0.056 | 48 |
| 3 | 8.663 | 966 | 0.098 | 186 |
| 4 | 12.140 | 4810 | 0.490 | 715 |
| 5 | 16.771 | 1393 | 0.142 | 151 |
| 6 | 18.501 | 964452 | 98.238 | 87191 |
| 7 | 27.954 | 6630 | 0.675 | 360 |
| 8 | 38.241 | 1646 | 0.168 | 204 |
| 总计 | | 981752 | 100.000 | 89597 |

截取 190nm | Ch1 254nm

**Figure S11.** High performance liquid chromatography (HPLC) analysis of compound **3**

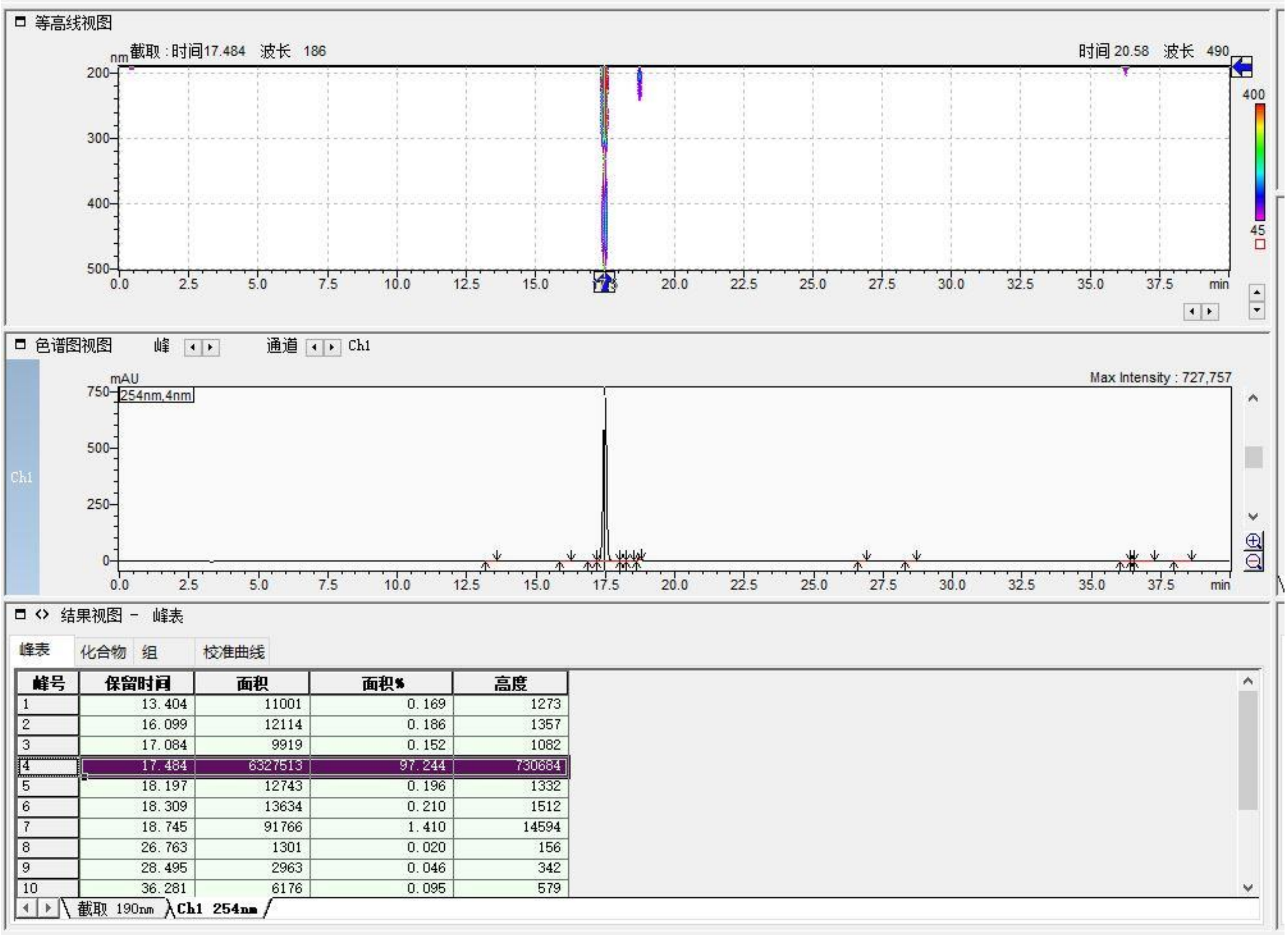


| 峰号 | 保留时间 | 面积 | 面积% | 高度 |
|---|---|---|---|---|
| 1 | 13.404 | 11001 | 0.169 | 1273 |
| 2 | 16.099 | 12114 | 0.186 | 1357 |
| 3 | 17.084 | 9919 | 0.152 | 1082 |
| 4 | 17.484 | 6327513 | 97.244 | 730684 |
| 5 | 18.197 | 12743 | 0.196 | 1332 |
| 6 | 18.309 | 13634 | 0.210 | 1512 |
| 7 | 18.745 | 91766 | 1.410 | 14594 |
| 8 | 26.763 | 1301 | 0.020 | 156 |
| 9 | 28.495 | 2963 | 0.046 | 342 |
| 10 | 36.281 | 6176 | 0.095 | 579 |

**Figure S12.** High performance liquid chromatography (HPLC) analysis of compound **4**

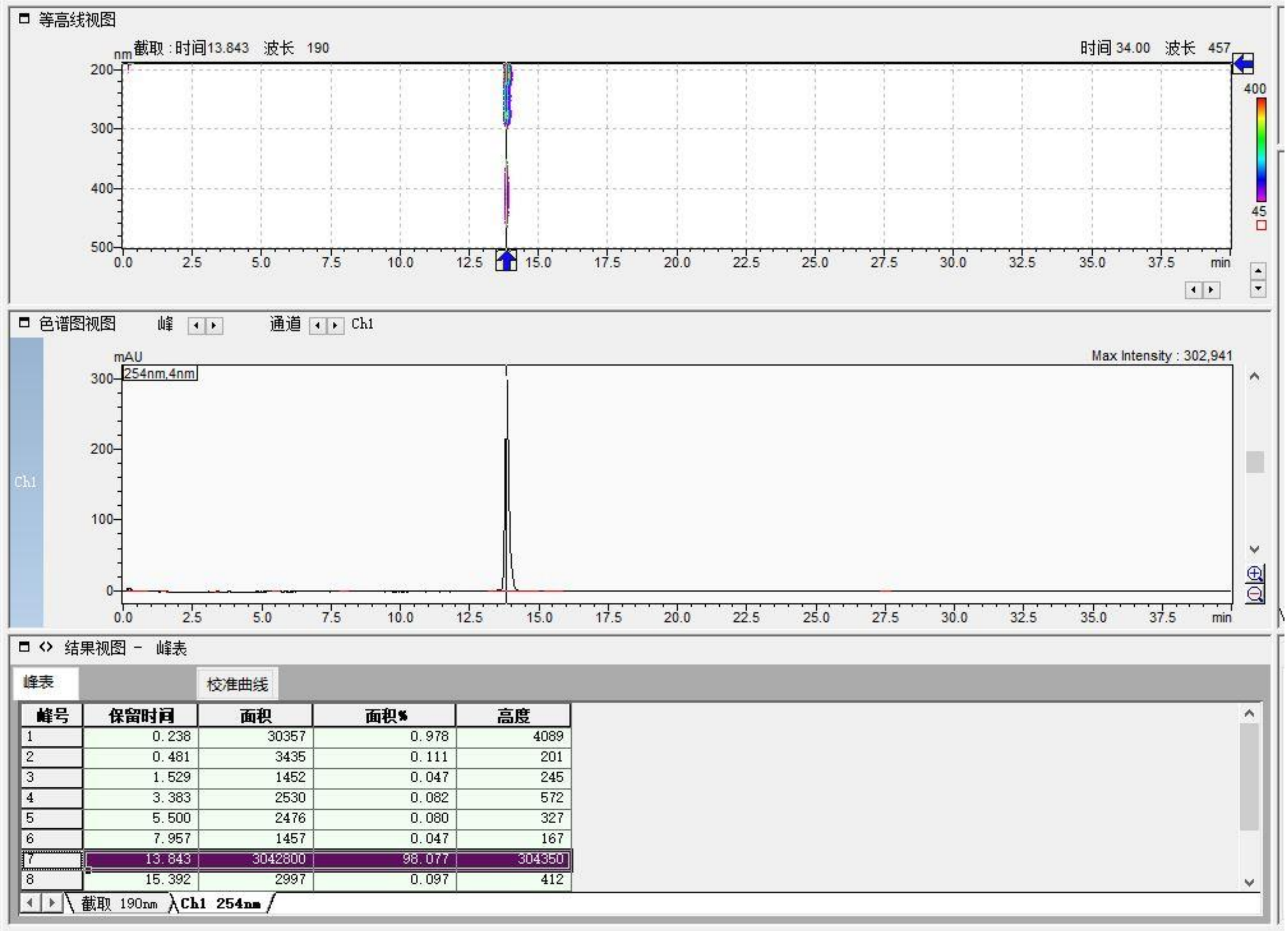


| 峰号 | 保留时间 | 面积 | 面积% | 高度 |
|---|---|---|---|---|
| 1 | 0.238 | 30357 | 0.978 | 4089 |
| 2 | 0.481 | 3435 | 0.111 | 201 |
| 3 | 1.529 | 1452 | 0.047 | 245 |
| 4 | 3.383 | 2530 | 0.082 | 572 |
| 5 | 5.500 | 2476 | 0.080 | 327 |
| 6 | 7.957 | 1457 | 0.047 | 167 |
| 7 | 13.843 | 3042800 | 98.077 | 304350 |
| 8 | 15.392 | 2997 | 0.097 | 412 |

**Figure S13.** High performance liquid chromatography (HPLC) analysis of compound **5**

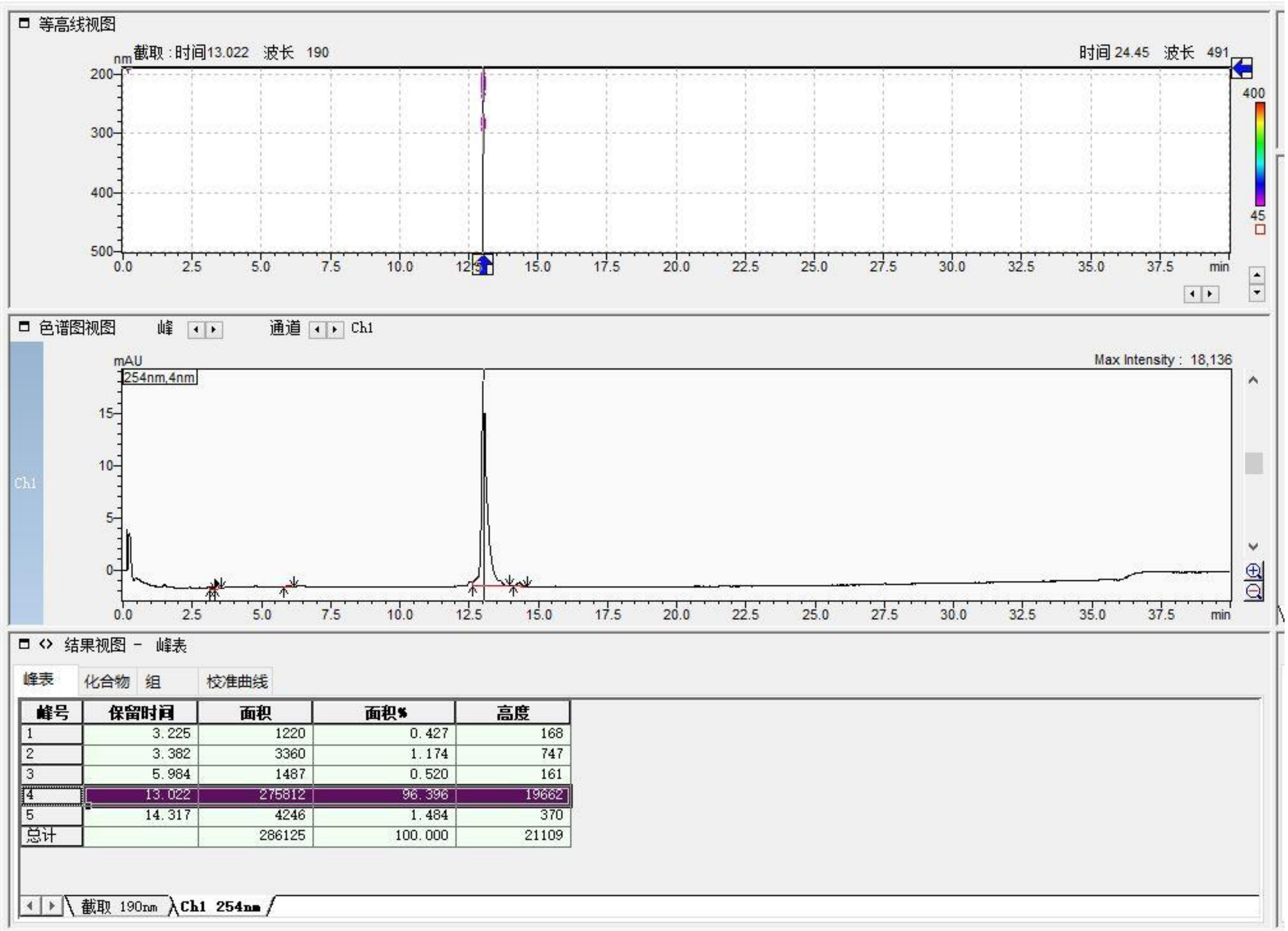


| 峰号 | 保留时间 | 面积 | 面积% | 高度 |
|---|---|---|---|---|
| 1 | 3.225 | 1220 | 0.427 | 168 |
| 2 | 3.382 | 3360 | 1.174 | 747 |
| 3 | 5.984 | 1487 | 0.520 | 161 |
| 4 | 13.022 | 275812 | 96.396 | 19662 |
| 5 | 14.317 | 4246 | 1.484 | 370 |
| 总计 | | 286125 | 100.000 | 21109 |

**Figure S14.** High performance liquid chromatography (HPLC) analysis of compound **6**

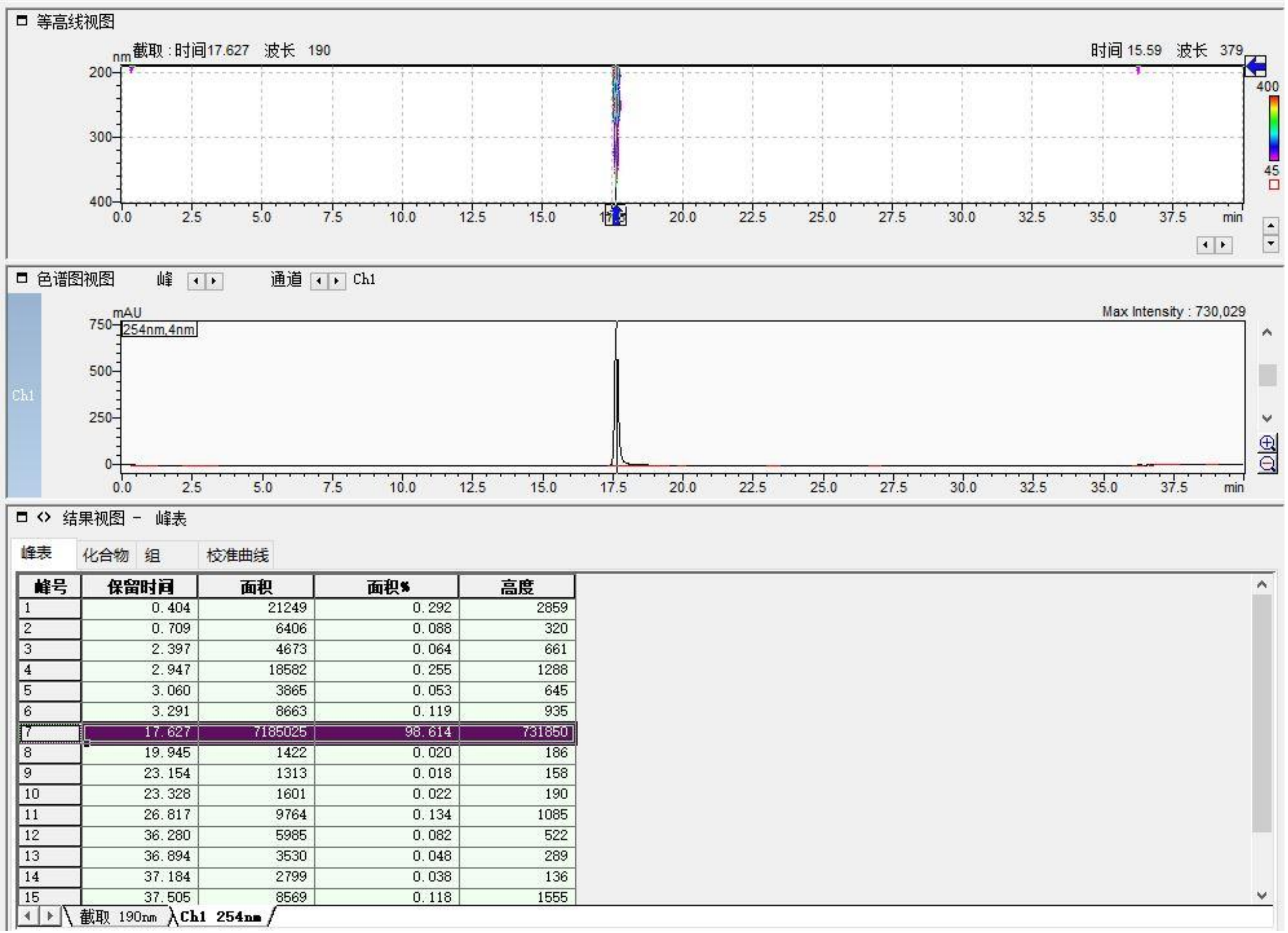


| 峰号 | 保留时间 | 面积 | 面积% | 高度 |
|---|---|---|---|---|
| 1 | 0.404 | 21249 | 0.292 | 2859 |
| 2 | 0.709 | 6406 | 0.088 | 320 |
| 3 | 2.397 | 4673 | 0.064 | 661 |
| 4 | 2.947 | 18582 | 0.255 | 1288 |
| 5 | 3.060 | 3865 | 0.053 | 645 |
| 6 | 3.291 | 8663 | 0.119 | 935 |
| 7 | 17.627 | 7185025 | 98.614 | 731850 |
| 8 | 19.945 | 1422 | 0.020 | 186 |
| 9 | 23.154 | 1313 | 0.018 | 158 |
| 10 | 23.328 | 1601 | 0.022 | 190 |
| 11 | 26.817 | 9764 | 0.134 | 1085 |
| 12 | 36.280 | 5985 | 0.082 | 522 |
| 13 | 36.894 | 3530 | 0.048 | 289 |
| 14 | 37.184 | 2799 | 0.038 | 136 |
| 15 | 37.505 | 8569 | 0.118 | 1555 |

**Figure S15.** High performance liquid chromatography (HPLC) analysis of compound **7**

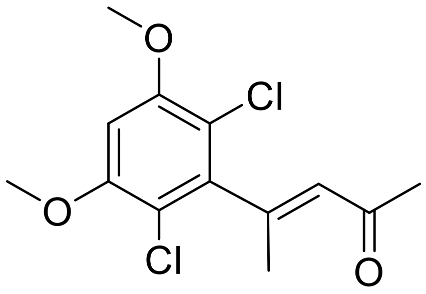


**T5**

**Figure S16.** The structure of T5 in the Chinese patent CN202010970367.1 (Dichlororesorcin compound, its preparation method and medical application)
